# Supplementary material for: Nivolumab plus ipilimumab induce hyper-progression in renal medullary carcinoma: results of a phase II trial and preclinical evidence
Source: Nat Commun. 2025 Nov 25;16:10474. doi: 10.1038/s41467-025-65462-z (PMC12647744; doi:10.1038/s41467-025-65462-z)
Supplement: Supplementary file 1 — Supplementary Information [file 41467_2025_65462_MOESM1_ESM.pdf]

Supplementary Information

Nivolumab plus ipilimumab induces hyperprogression in renal medullary carcinoma: results of a phase II trial and preclinical evidence

Melinda Soeung[1,†], Xinmiao Yan[1,†], Ciro Zanca[2], Jing Qian[3,4], Menuka Karki[3,4], Fei Duan[3,4], Hania Khan[3], Li Zhang[3], David H. Peng[2], Mariah Williams[2], Rong He[3,4], Ziheng Chen[1], Luigi Perelli[3,5], Jianfeng Chen[3,4], Rebecca S. Tidwell[6], Pankaj K. Chauhan[3,4], Courtney N. Le[3], Truong N.A. Lam[3], Nirjar Bhattacharya[1], Rutvi Shah[1,7], I-Lin Ho[1], Jason P. Gay[2], Caroline C. Carrillo[2], Ningping Feng[2], Kang Le[8], Guang Gao[2], Teresa L. Perry[8], Faika Mseeh[8], Yongying Jiang[8], Quanyun A. Xu[8], Niki Marie Zacharias[9], Rahul A. Sheth[10], Tharakeswara K. Bathala[11], Priya Rao[12], Najat C. Daw[13], Durga N. Tripathi[14], Cheryl L. Walker[14], Mohammad M. Mohammad[15], Jianhua Zhang[1], Guangchun Han[1], Yanshuo Chu[1], Ruiping Wang[1], Minghao Dang[1], Enyu Dai[1], Fuduan Peng[1], Yunhe Liu[1], Akshaya Jadhav[16], Wenhua Lang[16], Claudio A. Arrechedera[16], Leticia Campos Clemente[16], Edwin R. Parra[16], Hsinyi Lu[16], Cara L. Haymaker[16], Ignacio I. Wistuba[16], Andrew Futreal[1], Andrea Viale[1], , Michael J. Soth[8], Philip Jones[8], Joseph R. Marszalek[8],Timothy Heffernan[2], Giulio F. Draetta[2], Nizar M. Tannir[3], Jianjun Gao[3,4,‡,*], Linghua Wang[1,7,17,18,‡,*], Giannicola Genovese[1,2,3,4,‡,*], Pavlos Msaouel[3,4,7,16,‡,*]

[1]Department of Genomic Medicine, The University of Texas MD Anderson Cancer Center, Houston, TX 77038, USA.
[2]Translational Research to Advance Therapeutics and Innovation in Oncology (TRACTION), The University of Texas MD Anderson Cancer Center, Houston, TX 77030, USA.
[3]Department of Genitourinary Medical Oncology, The University of Texas MD Anderson Cancer Center, Houston, TX 77030, USA.
[4]David H. Koch Center for Applied Research of Genitourinary Cancers, The University of Texas, MD Anderson Cancer Center, Houston, TX 77030, USA.
[5]Department of Cancer Biology, The University of Texas, MD Anderson Cancer Center, Houston, TX 77030, USA.
[6]Department of Biostatistics, The University of Texas MD Anderson Cancer Center, Houston, TX 77030, USA.
[7]The University of Texas MD Anderson Cancer Center UTHealth Houston Graduate School of Biomedical Sciences, Houston, TX 77030, USA.
[8]Institute for Applied Cancer Science (IACS), Therapeutics Discovery Division, The University of Texas MD Anderson Cancer Center, Houston, Texas 77054, United States
[9]Department of Urology, The University of Texas MD Anderson Cancer Center, Houston, TX 77038, USA.
[10]Department of Interventional Radiology, The University of Texas MD Anderson Cancer Center, Houston, TX 77030, USA.
[11]Department of Diagnostic Imaging, The University of Texas MD Anderson Cancer Center, Houston, TX 77030, USA.
[12]Department of Pathology, The University of Texas MD Anderson Cancer Center, Houston, TX 77030, USA.
[13]Department of Pediatrics, The University of Texas MD Anderson Cancer Center, Houston, TX 77030, USA.
[14]Center for Precision Environmental Health, Baylor college of Medicine, Houston, TX 77030, USA.
[15]Institute for Personalized Cancer Therapy, The University of Texas MD Anderson Cancer Center, Houston, TX 77030, USA.
[16]Department of Translational Molecular Pathology, The University of Texas MD Anderson Cancer Center, Houston, TX 77030, USA.
[17]The James P. Allison Institute, The University of Texas MD Anderson Cancer Center, Houston, TX 77030, USA.
[18]Institute for Data Science in Oncology, The University of Texas MD Anderson Cancer Center, Houston, TX 77030, USA.
[†]These authors contributed equally to this work: Melinda Soeung, Xinmiao Yan
[‡]These authors jointly supervised this work: Pavlos Msaouel, Giannicola Genovese, Linghua Wang, Jianjun Gao
*Correspondence to: pmsaouel@mdanderson.org; ggenovese@mdanderson.org; lwang22@mdanderson.org; jgao1@mdanderson.org

Table of Contents

Supplementary Figures:

Supplementary Figure S1. Trial schema (NCT03274258). Page 4
Supplementary Figure S2. Single-cell clustering and annotation. Page 5
Supplementary Figure S3. Sub-clustering and annotation of T&NK cells. Page 6
Supplementary Figure S4. Inferred large-scale copy number variations Page 7
Supplementary Figure S5. Volcano plot showing the significant DEGs between baseline and PostNI tumor cells. Page 8
Supplementary Figure S6. UMAP of unsupervised clustering analysis of malignant cells derived only from the liver. Page 9
Supplementary Figure S7. Volcano plot of scRNA-seq differential gene expression using only tumor cells derived from the liver. Page 10
Supplementary Figure S8. Violin plots of scRNA-seq differential gene expression using only tumor cells derived from the liver. Page 11
Supplementary Figure S9. Workflow of clean-up analysis of CyTOF (mass cytometry) data. Page 12
Supplementary Figure S10. Gating strategy for flow CyTOF (mass cytometry) analysis. Page 13
Supplementary Figure S11. Heatmap showing major clusters identified from CyTOF (mass cytometry) analysis. Page 14
Supplementary Figure S12. t-SNE plot of major clusters identified from CyTOF analysis based on each patient sample. Page 15
Supplementary Figure S13. Results of CyTOF (mass cytometry) for peripheral blood samples. Page 16
Supplementary Figure S14. Results of multiplex immunofluorescence of patient samples. Page 17
Supplementary Figure S15. GSEA of bulk RNA sequencing of longitudinally collected tumor tissues. Page 18
Supplementary Figure S16. UMAP of unsupervised clustering analysis of all malignant and myeloid cells. Page 18
Supplementary Figure S17. UMAP of unsupervised clustering analysis of malignant and myeloid cells from liver biopsies. Page 19
Supplementary Figure S18. Euclidean and Manhattan distances of liver samples containing tumor cells. Page 19
Supplementary Figure S19. Differential cell-cell communications between PostNI and baseline in liver metastasis. Page 20
Supplementary Figure S20. Multiplex immunofluorescent analysis of CD68 in MSRT1 ex vivo tumors. Page 21
Supplementary Figure S21. Representative immunofluorescent images of F4/80. Page 21
Supplementary Figure S22. Immunofluorescent images and quantification of S100A9 in MSRT1 cells treated with IFNγ. Page 22
Supplementary Figure S23. Quantification of F4/80 in immunofluorescent study of MSRT1 cells treated with IFNγ. Page 22
Supplementary Figure S24. Upregulation of mouse myeloid lineage markers in following in MSRT1 treated with IFNγ. Page 23
Supplementary Figure S25. BromoMAX results against 32 bromodomain containing proteins. Page 24
Supplementary Figure S26. Western blot analysis of DOHH2 cells treated with IACS-16898. Page 24
Supplementary Figure S27. Inhibition of DOHH2 cell proliferation by IACS-16898. Page 25
Supplementary Figure S28. IACS-16898 inhibits tumor growth in the DOHH2 subcutaneous xenograft model. Page 25
Supplementary Figure S29. Body weights as measured throughout the treatment with vehicle or IACS-16898. Page 26
Supplementary Figure S30. Plasma PK correlation between IACS-16898 exposure and BCL6 mRNA target inhibition. Page 26
Supplementary Figure S31. Immunofluorescence of S100A9 in treated tumors. Page 27
Supplementary Figure S32. Quantification of tumor cells labeled with GFP co-expressing S100A9 in RMC mouse models. Page 28
Supplementary Figure S33. Uncropped scanned films of western blots presented in Figure 6a. Page 29

Supplementary Figure S34. Uncropped scanned films of western blots presented in Figure 6b. Page 30
Supplementary Figure S35. Uncropped scanned films of western blots presented in Figure 7c. Page 31
Supplementary Figure S36. Uncropped scanned films of western blots presented in Figure 8d. Page 32
Supplementary Figure S37. Uncropped scanned films of western blots presented in Supplementary Figure S26. Page 33

Supplementary Tables:

Supplementary Table S1. Treatment emergent adverse events at least possibly related to study therapy. Page 34
Supplementary Table S2. Treatment emergent adverse events regardless of attribution. Page 35
Supplementary Table S3. Patient samples used for single-cell RNA sequencing. Page 36
Supplementary Table S4. Number of tumor cells in scRNA-seq analysis. Page 36
Supplementary Table S5. Patient samples used for multiplex immunofluorescence. Page 36
Supplementary Table S6. Samples used for bulk RNA sequencing from patients enrolled in the clinical trial. Page 37
Supplementary Table S7. Demographics, clinical and prior treatment characteristics of the scRNA-seq patient cohort. Page 37
Supplementary Table S8. BromoKdELECT data of IACS-16898. Page 38
Supplementary Table S9. Selected bromoKdELECT data of IACS-16898. Page 38
Supplementary Table S10. *In vivo* pharmacokinetic exposure of IACS-16898 in mice. Page 39
Supplementary Table S11. *In vivo* pharmacokinetic properties of IACS-16898 in mouse, rat, dog and monkey. Page 39
Supplementary Table S12. Stopping criteria for excessive TOX or insufficient esponses. Page 40
Supplementary Table S13. Operating characteristics under varying toxicity and objective response rates. Page 40
Supplementary Table S14. Panel of anti-human antibodies for CyTOF (mass cytometry). Page 41
Supplementary Table S15. Antibodies used for immunohistochemistry, and Western blot protein analysis. Page 43
Supplementary Table S16. Panel antibody optimization by multiplex immunofluorescence for automated staining. Page 44
Supplementary Table S17. Panel antibody optimization by multiplex immunofluorescence for manual staining. Page 45

Supplementary Note. Clinical Trial Protocol. Page 46

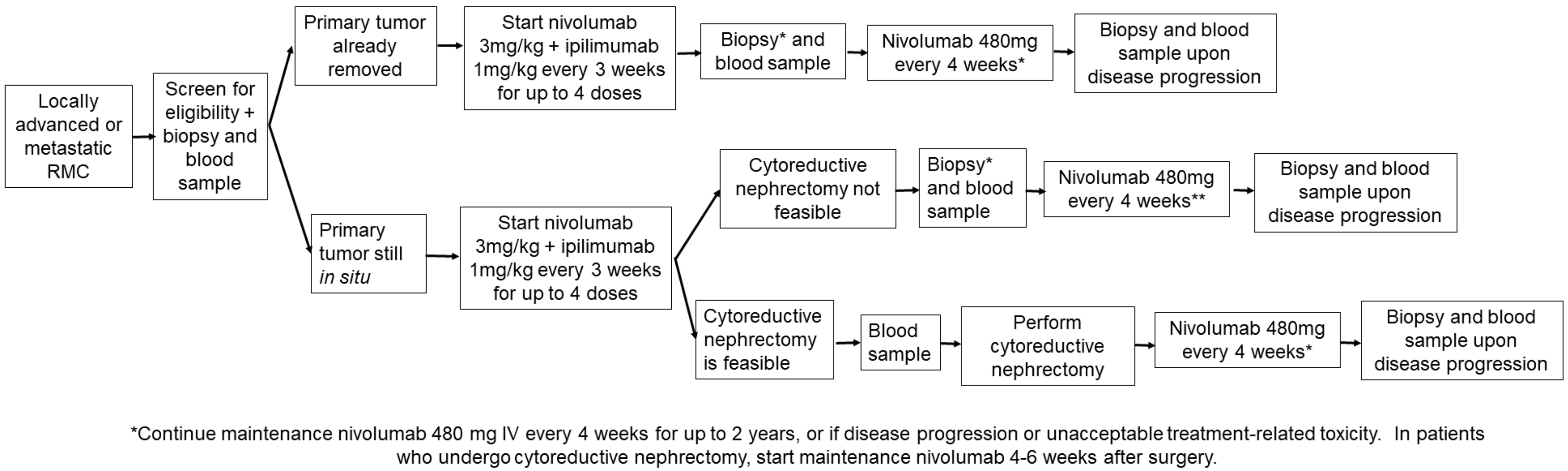

Supplementary Figure S1. Trial schema (NCT03274258). Peripheral blood and optional tumor biopsy samples were planned to be collected at baseline, and then at mid-study prior to initiation of maintenance nivolumab monotherapy, and finally at disease progression. If disease progression occurred prior to completion of combination nivolumab + ipilimumab then the mid-study collection would not occur, and peripheral blood and optional tumor biopsy samples would be collected only at baseline and at disease progression. Treatment continued until disease progression. Due to the aggressive progression following nivolumab plus ipilimumab in all patients on trial, the mid-study collection step was omitted.

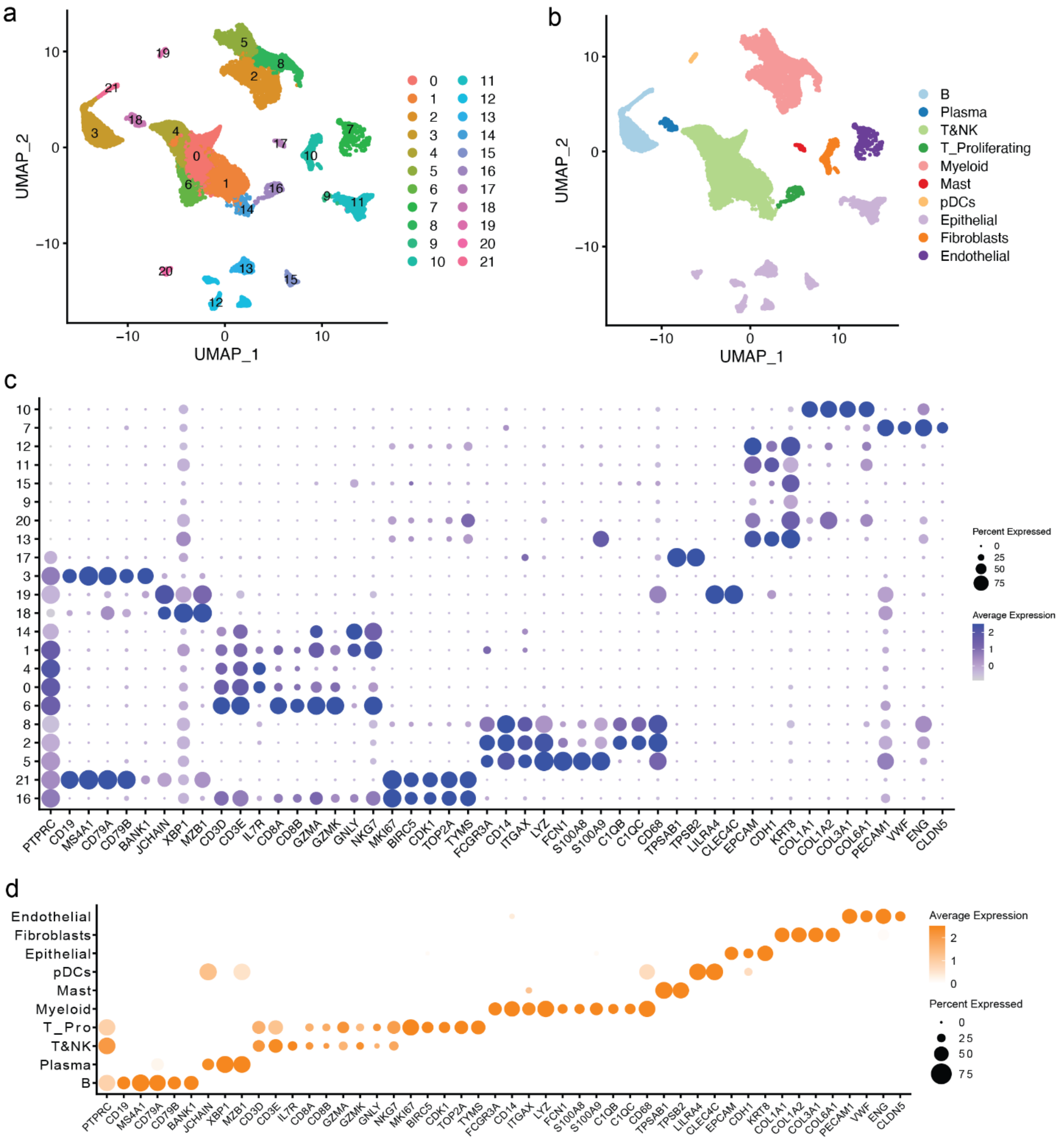

Supplementary Figure S2. Single-cell clustering and annotation for RMC patients treated with baseline (n = 5) and PostNI (n =2). (a) Umap plot shows an overview of 23,880 cells included for analyses, which formed 22 cell clusters. 22 cell clusters. **(b)** Umap plot shows the main cell types that have been annotated. **(c)** Dot plot shows the expression level of canonical cell markers across 22clusters. **(d)** Dot plot shows expression of lineage-specific marker genes across different cell types. Source data are provided as a Source Data file.

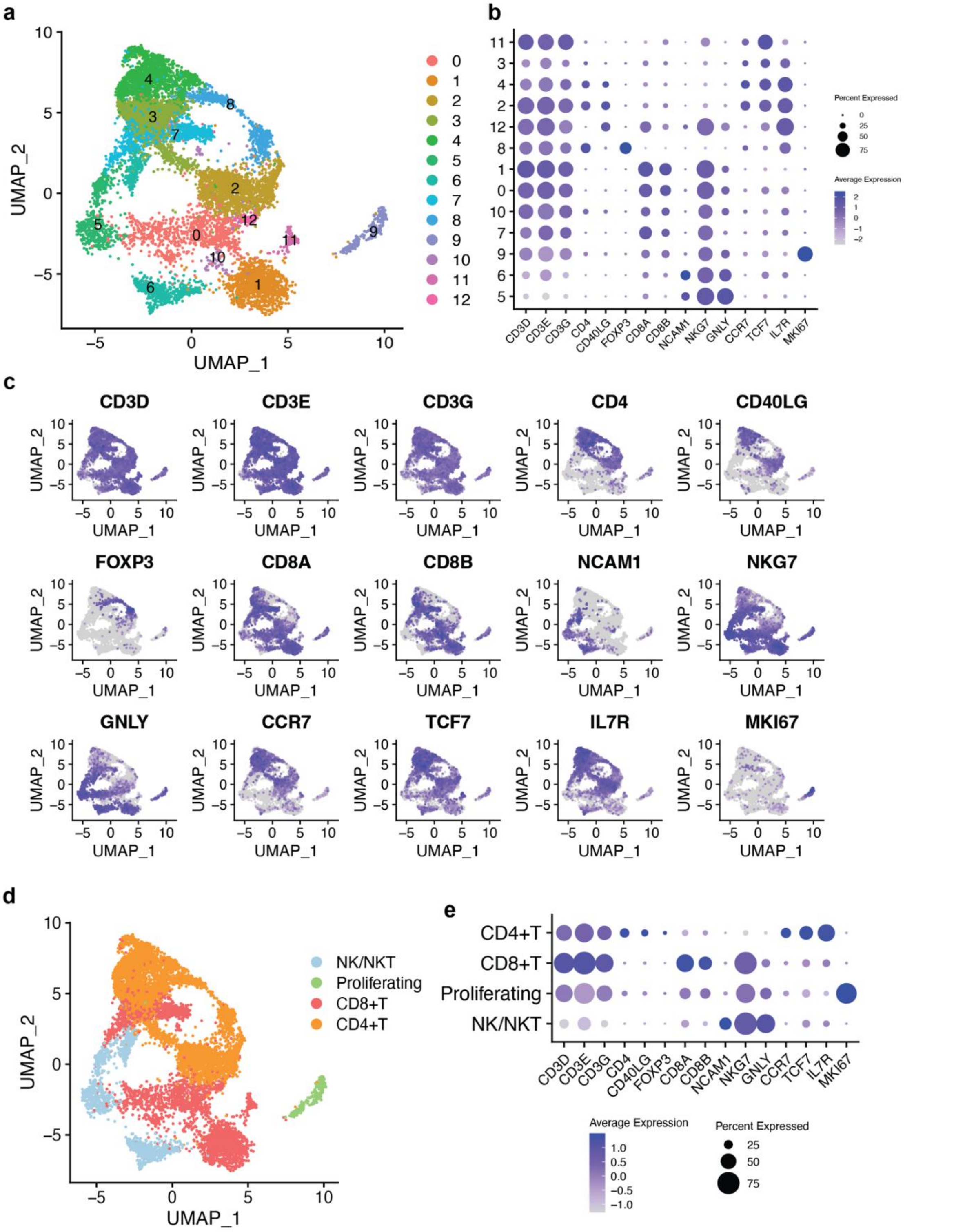

Supplementary Figure S3. Single-cell sub-clustering and annotation of T&NK cells for RMC patients treated with baseline (n = 5) and PostNI (n =2). (a) Umap plot shows an overview of 13 sub-clusters. **(b)** Dot plot shows the expression level of canonical cell markers across 13 sub-clusters. **(c)** Umap plot shows expression of canonical marker genes used for cell types assignment. **(d)** Umap plot shows the main cell types that have been annotated. **(e)** Dot plot shows the expression level of canonical cell markers across annotated cell types. Source data are provided as a Source Data file.

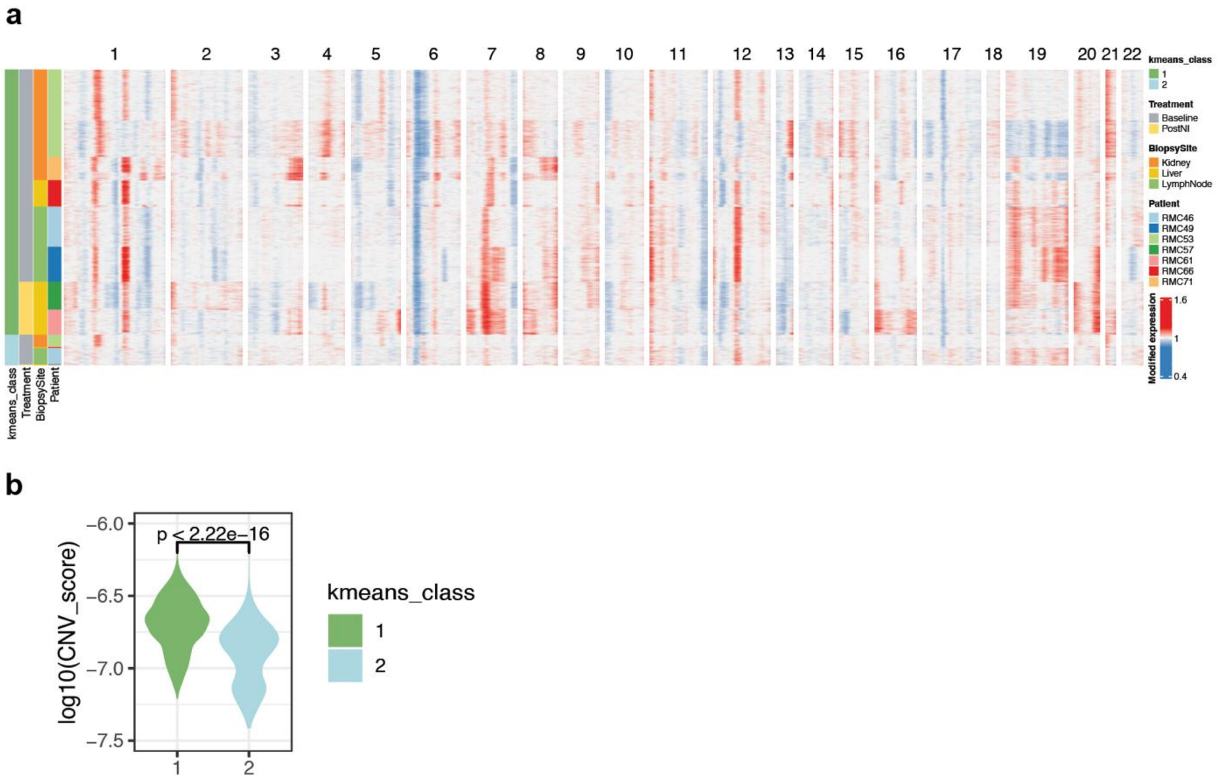

Supplementary Figure S4. Single-cell identification of malignant cells from epithelial cells for RMC patients at baseline (n = 5) and PostNI (n =2). (a) The landscape of inferred large-scale CNVs for all epithelial cells using T cells as the reference CNV cell state. Annotation tracks on the left indicate (from left to right) the corresponding hierarchical classification (1 or 2), Treatment (Baseline or following treatment with nivolumab plus ipilimumab [PostNI]), Biopsy site (kidney, liver or lymph node), and individual patient. Chromosome numbers are labeled at the top. **(b)** Violin plot showing the log10(CN_score) of kmeans class 1 and 2 cells. The cells in class1 were identified as the malignant tumor cells since they maintained a higher cnv score. Source data are provided as a Source Data file.

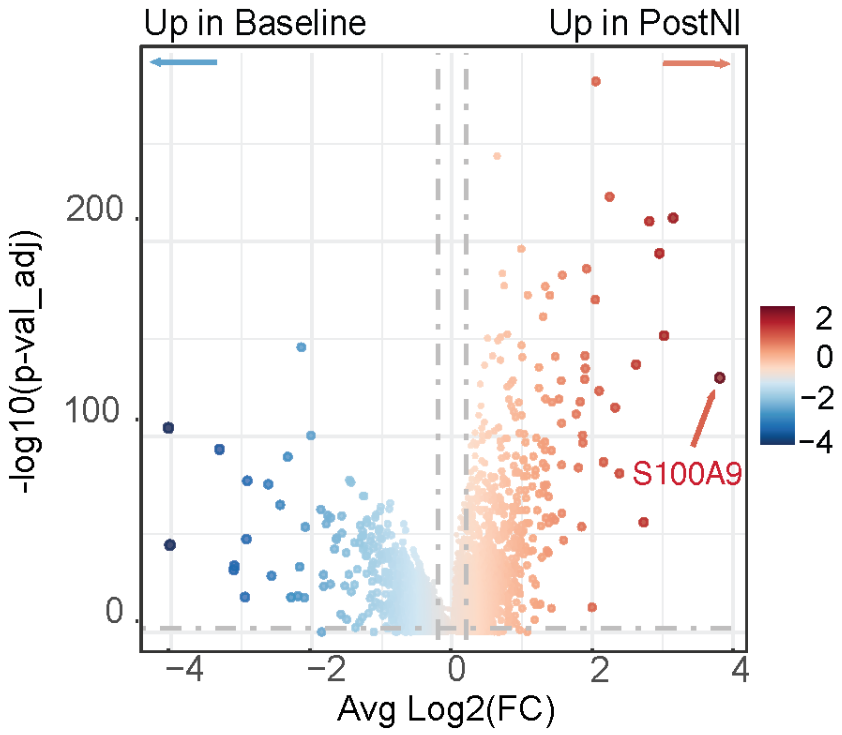

Supplementary Figure S5. Volcano plot showing the significant DEGs by single-cell RNA sequencing between baseline (n = 5) and PostNI (n = 2) tumor cells. S100 calcium-binding protein A9 (S100A9) is the top hit. Source data are provided as a Source Data file.

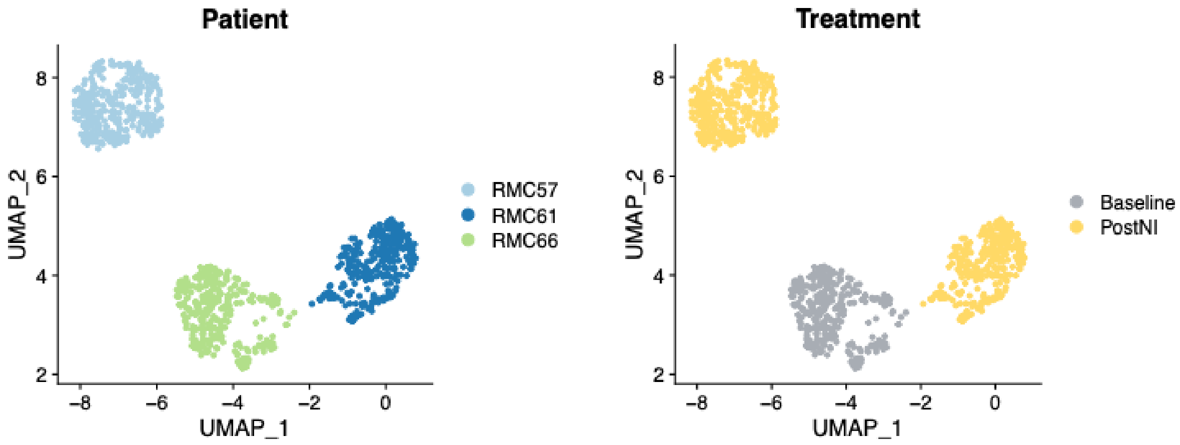

Supplementary Figure S6. UMAP of unsupervised clustering analysis from single-cell RNA sequencing of malignant cells derived only from the liver (Baseline n = 1 . PostNI n = 2). Cells are colored with patient id (left) and treatment condition (right). Source data are provided as a Source Data file.

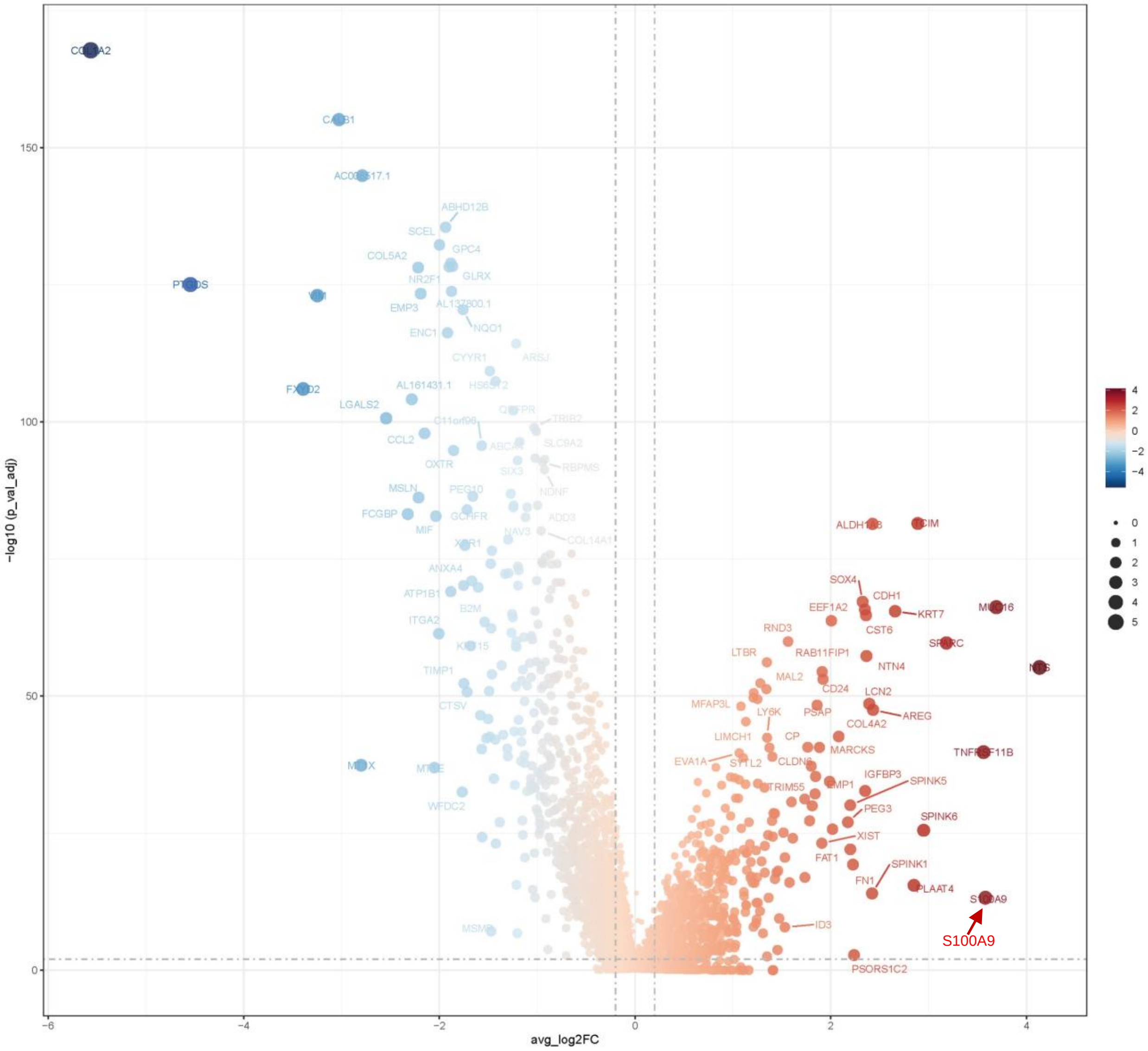

Supplementary Figure S7. Volcano plot of differential gene expression of scRNA-seq data using only tumor cells derived from the liver for a total of n=3 patients (n=1 baseline, n=2 postNI). S100 calcium-binding protein A9 (S100A9) is the third most upregulated gene. Source data are provided as a Source Data file.

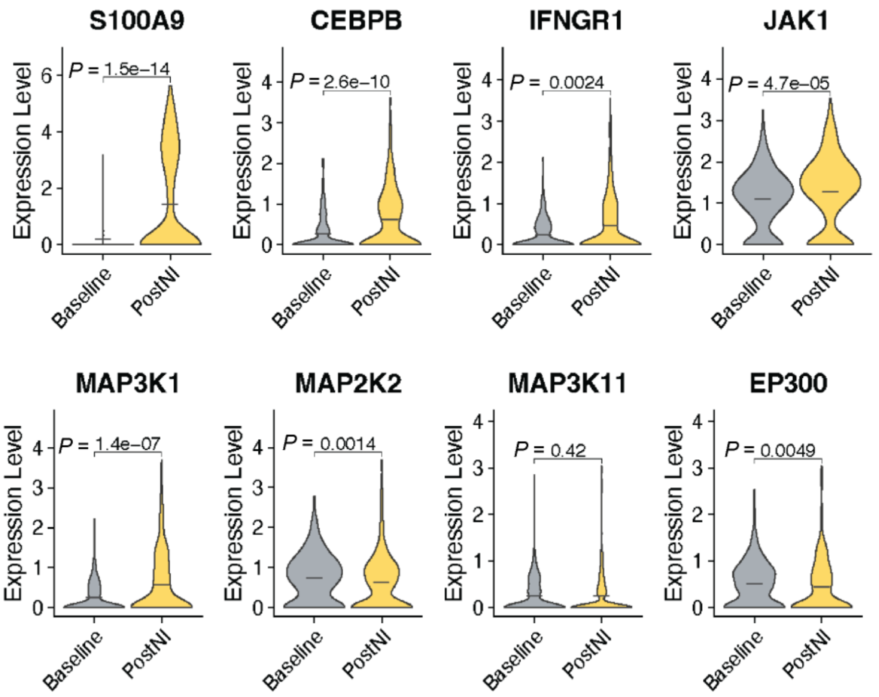

Supplementary Figure S8. Violin plots of differential gene expression of scRNA-seq data using only tumor cells derived from the liver for a total of n=3 patients (n=1 baseline, n=2 postNI). Source data are provided as a Source Data file.

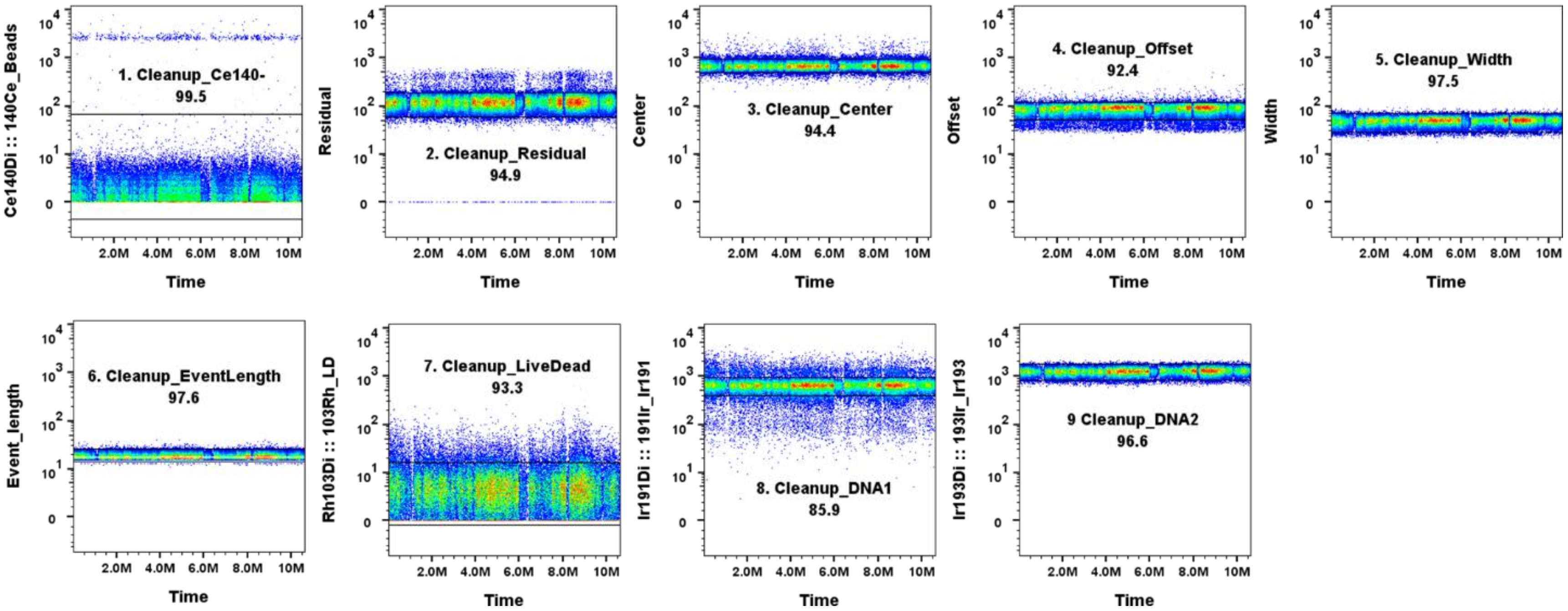

Supplementary Figure S9. **Workflow of clean-up analysis of CyTOF (mass cytometry) data.**

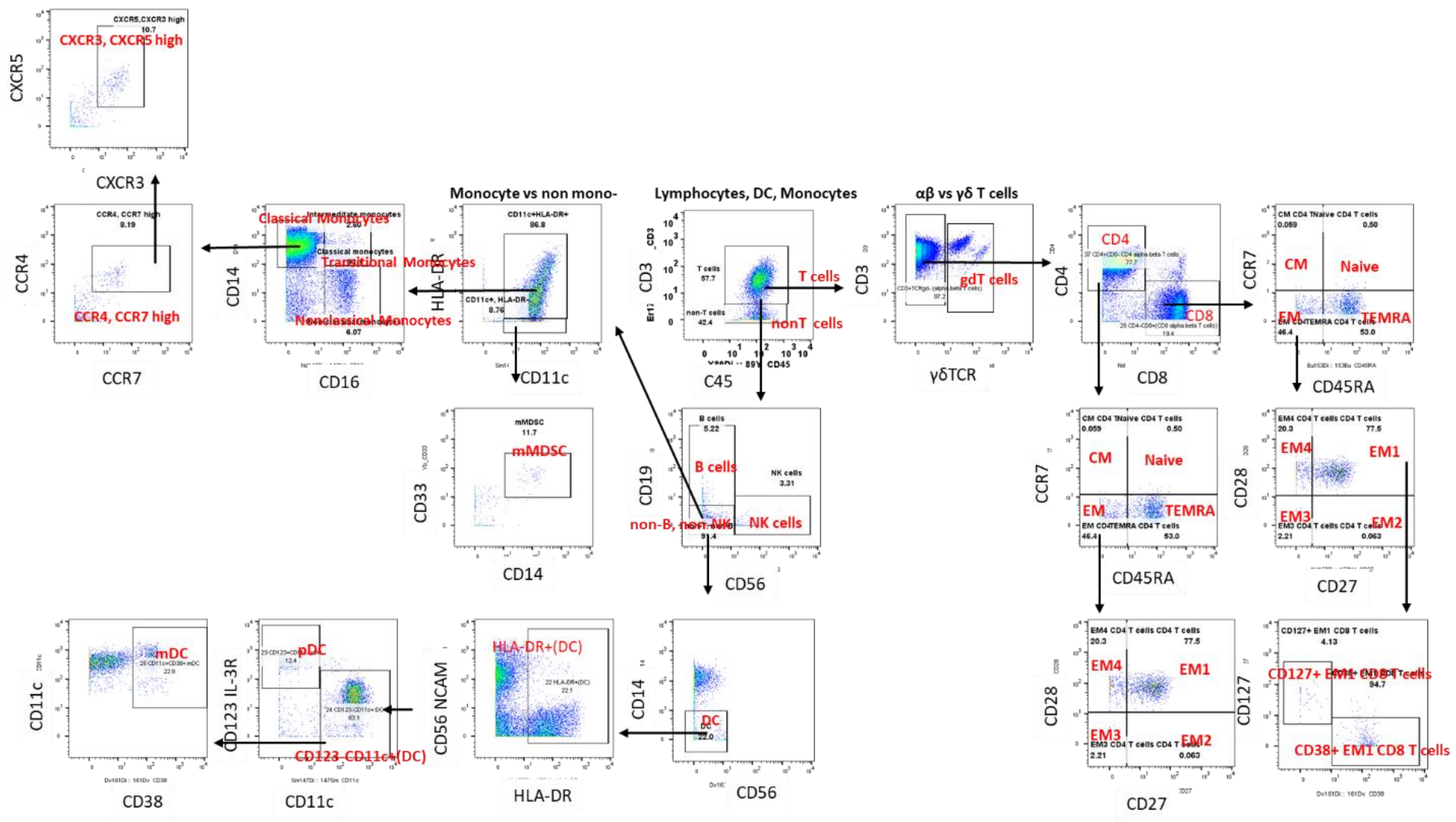

Supplementary Figure S10. Gating strategy for flow CyTOF (mass cytometry) analysis.

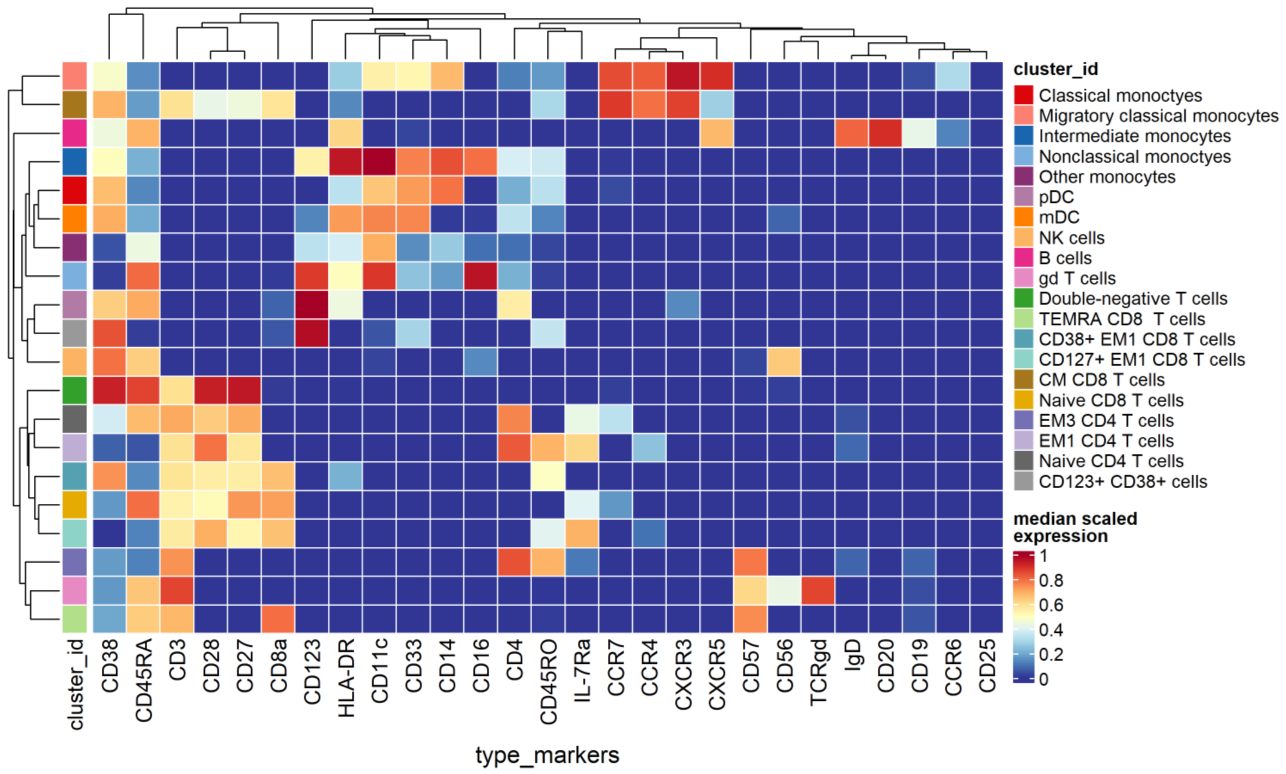

Supplementary Figure S11. Heatmap showing major clusters identified from CyTOF (mass cytometry) analysis. A total of 14 samples were included in this dataset, comprising 11 patient-derived samples (10 baseline and 3 post-immunotherapy timepoints) and one normal donor (ND) PBMC control.

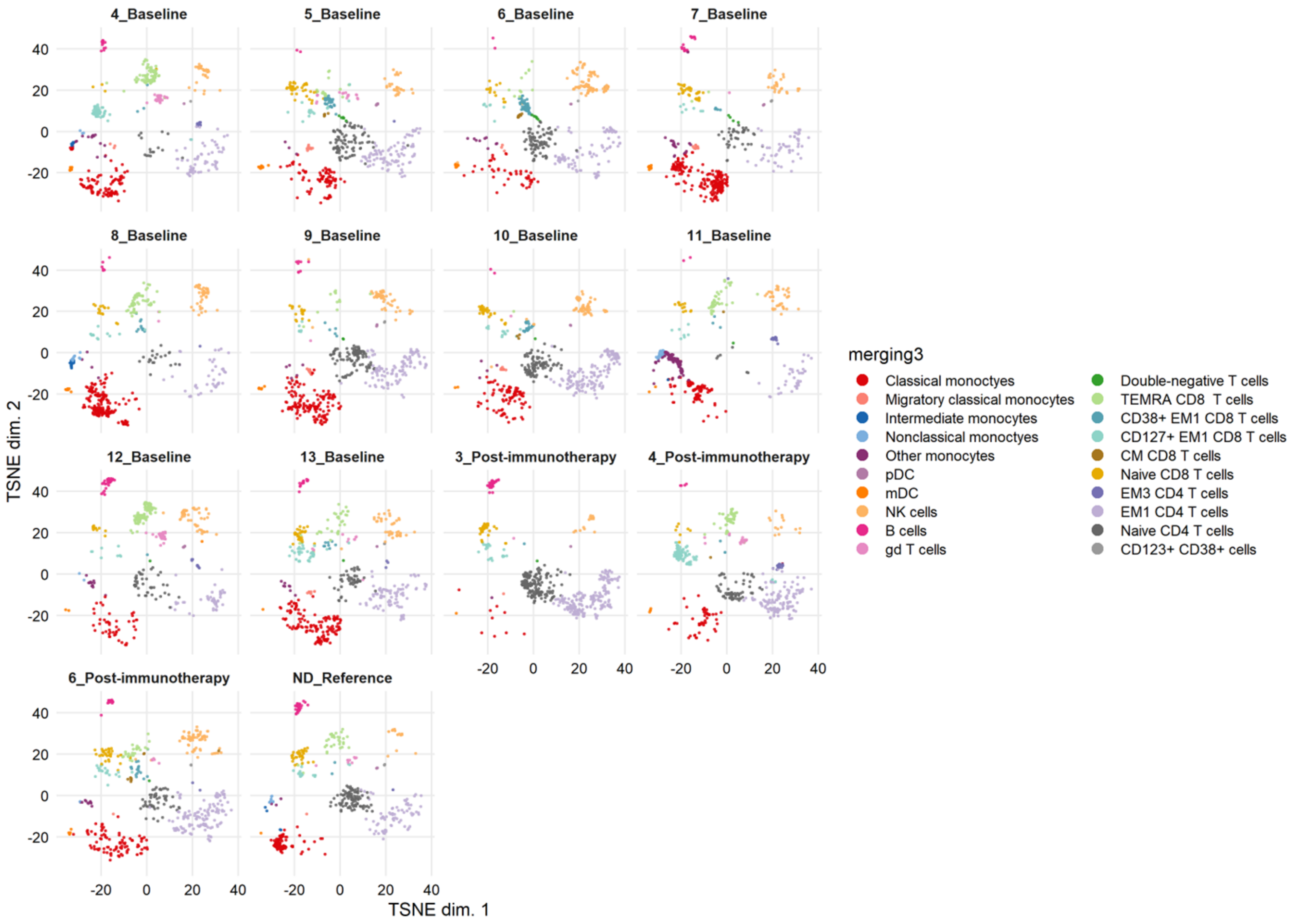

Supplementary Figure S12. t-SNE (T-distributed stochastic neighbor embedding) plot of major clusters identified from CyTOF (mass cytometry) analysis based on each patient sample. There are n = 10 patient samples from the baseline group and n = 3 patient samples from the post-immunotherapy (PostNI) group. A reference consisting of control normal donor blood that is run with each batch was used to normalize the results.

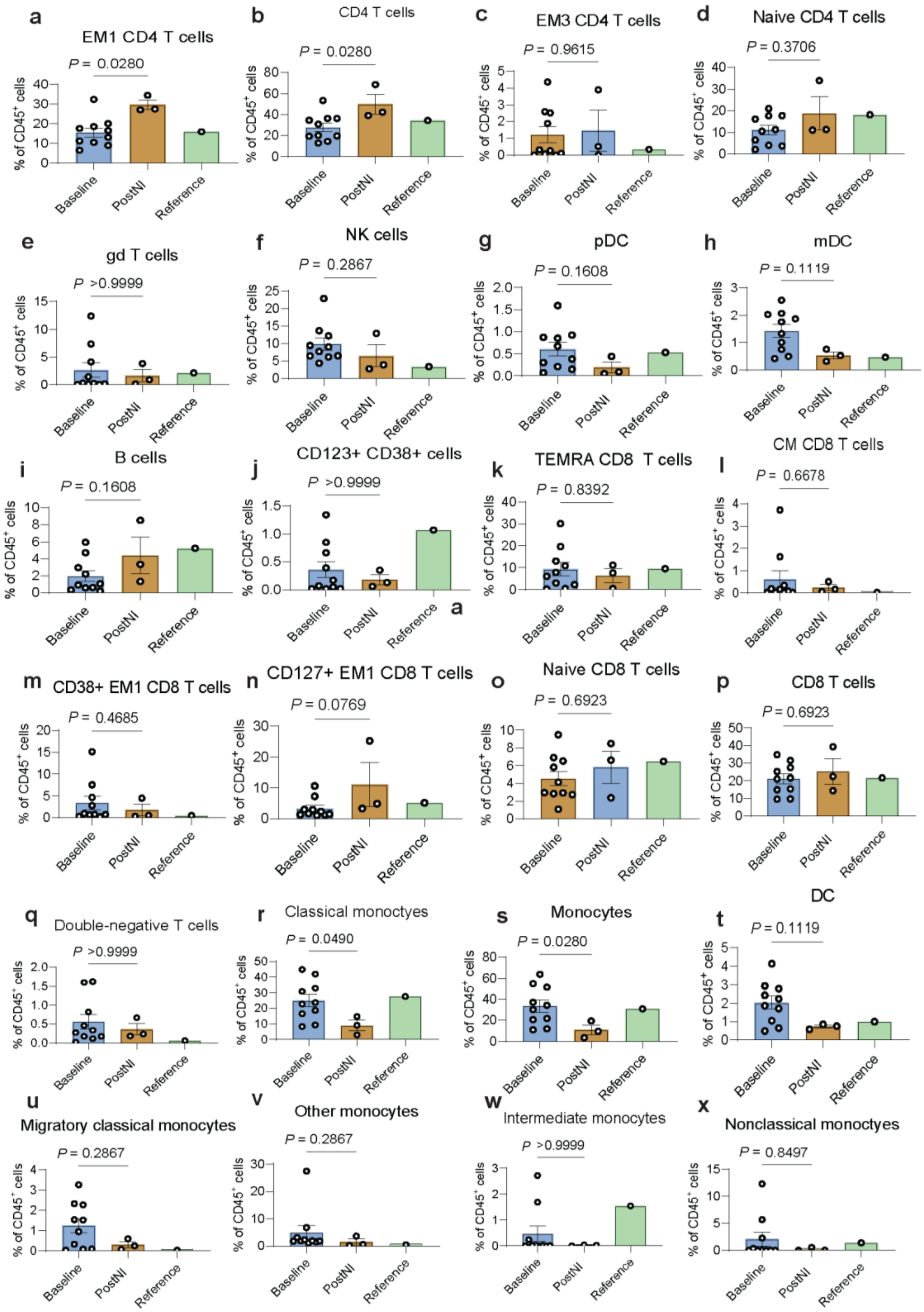

Supplementary Figure S13. **Results of CyTOF (mass cytometry) for peripheral blood samples from patients not treated with immune checkpoint therapy (baseline, n = 10), patients treated with nivolumab and ipilimumab (PostNI, n = 3), and control normal donor blood (reference, n = 1).** Data are expressed as mean value ± SD, with P value calculated by student's t test comparing only Baseline versus PostNI, and excluding the reference. Samples are biological replicates representing different patients. Source data are provided as a Source Data file.

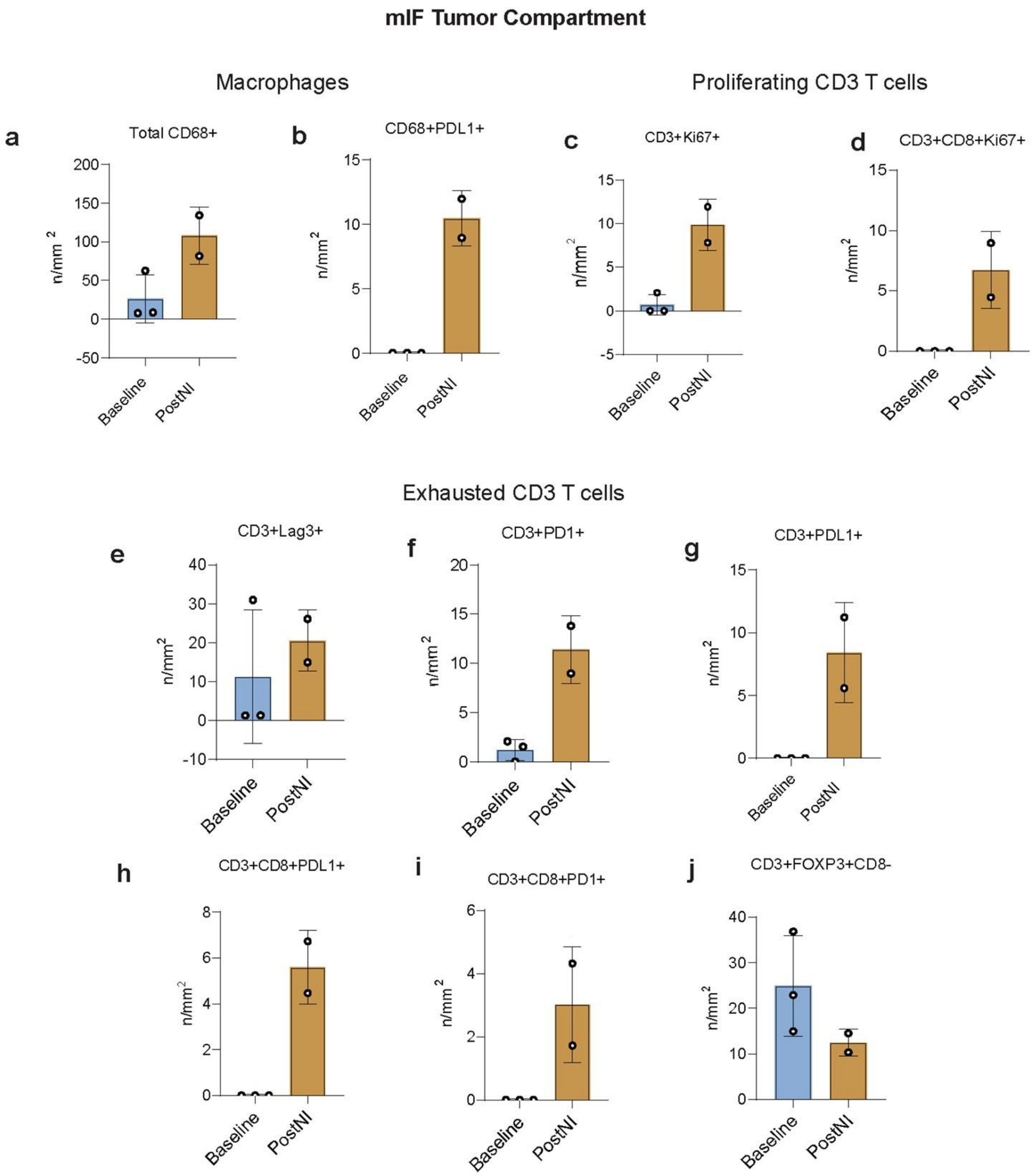

Supplementary Figure S14. **Results of multiplex immunofluorescence of patients not treated with immune checkpoint therapy (Baseline, n = 3) and patients treated with nivolumab and ipilimumab (PostNI, n = 2).** Data are expressed as mean value ± SD, and samples are biological replicates representing different patients. Source data are provided as a Source Data file.

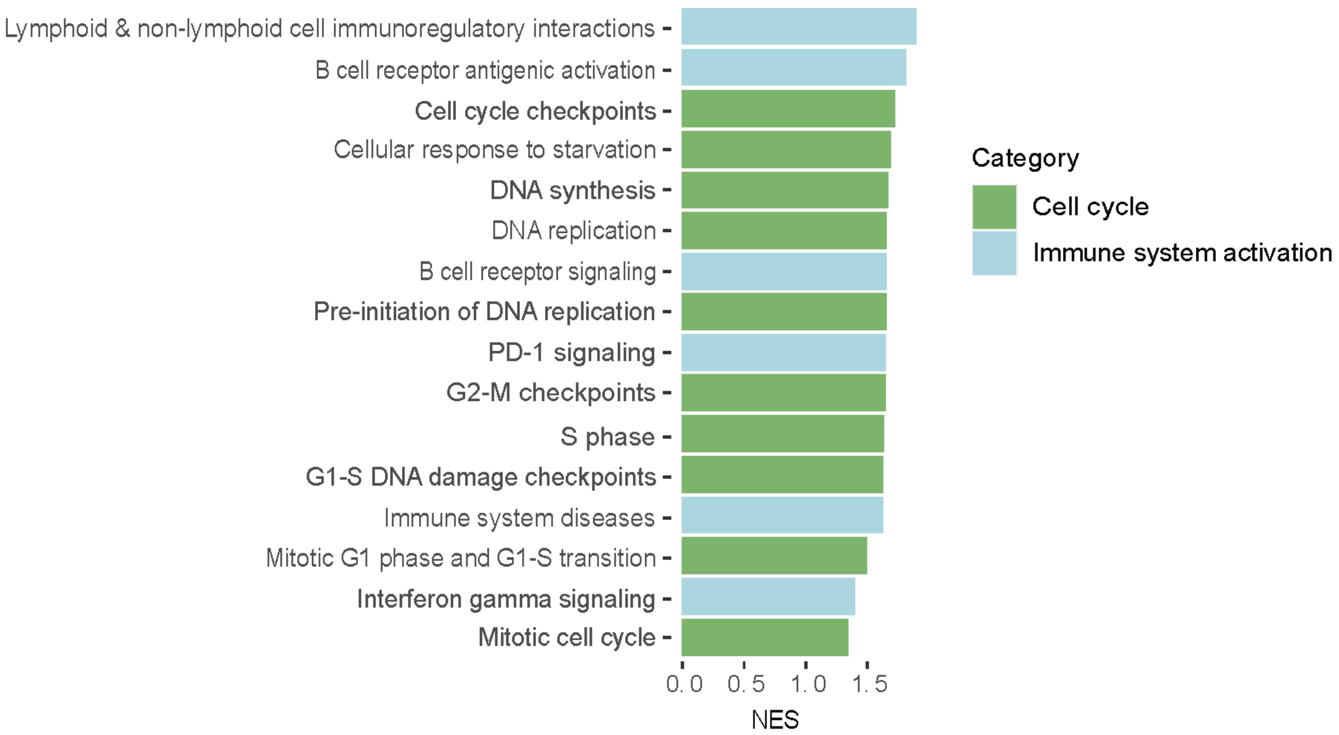

Supplementary Figure S15. Gene set enrichment analysis (GSEA) of bulk RNA sequencing of longitudinally collected tumor tissues from patients treated with nivolumab plus ipilimumab (n = 3) compared to treatment naïve patients (n = 3). Significant pathways for cell cycle and immune system activation were selected for visualization. Source data are provided as a Source Data file.

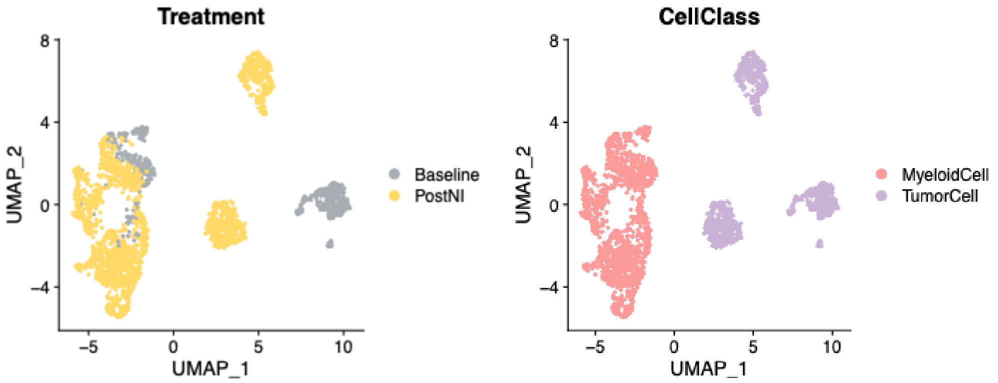

Supplementary Figure S16. UMAP of unsupervised clustering analysis of malignant and myeloid cells from liver biopsies (n=4). RMC patients treated with baseline (n = 1) and PostNI (n =2). Patient RMC56 was removed from the final analysis due to lack of tumor cells. Source data are provided as a Source Data file.

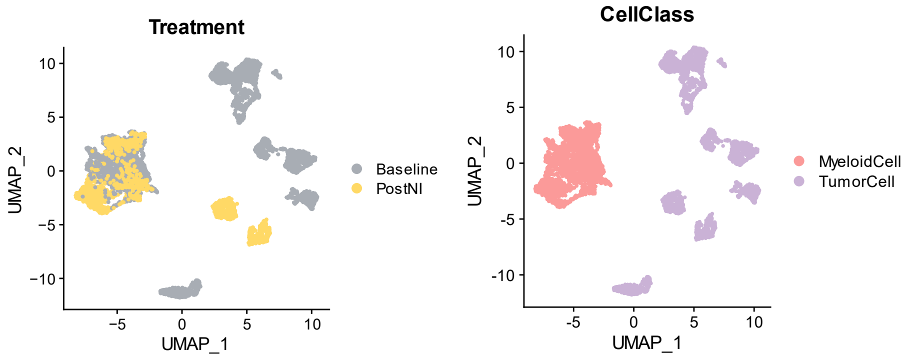

Supplementary Figure S17. UMAP of unsupervised clustering analysis of all malignant and myeloid cells for RMC patients treated with baseline (n = 5) and PostNI (n =2). Source data are provided as a Source Data file.

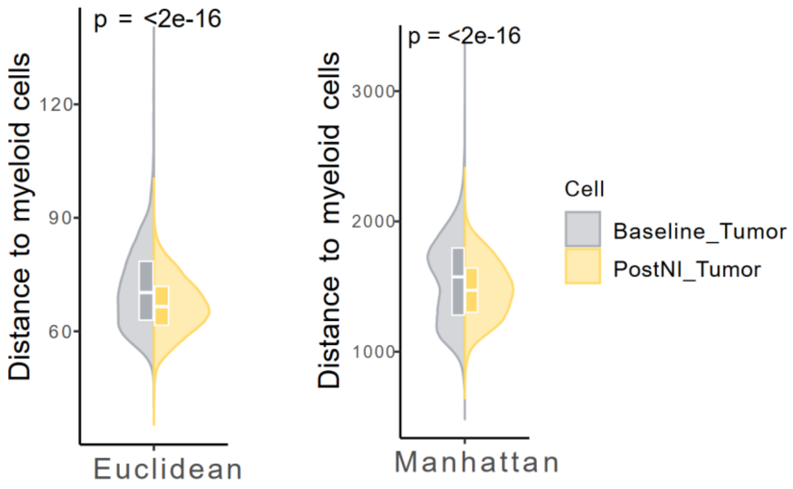

Supplementary Figure S18. Violin plot shows the Euclidean and Manhattan distances of all malignant tumor cells from all biopsy sites (n = 7) comparing the transcriptional differences between baseline (n = 5) tumors and tumors treated with combination ICT (nivolumab plus ipilimumab, PostNI, n = 2). Box-and-whisker plots show all values with range (whiskers), interquartile range (box) and mean (center line). The p-value was calculated using the two-sided Wilcoxon rank-sum test. Source data are provided as a Source Data file.

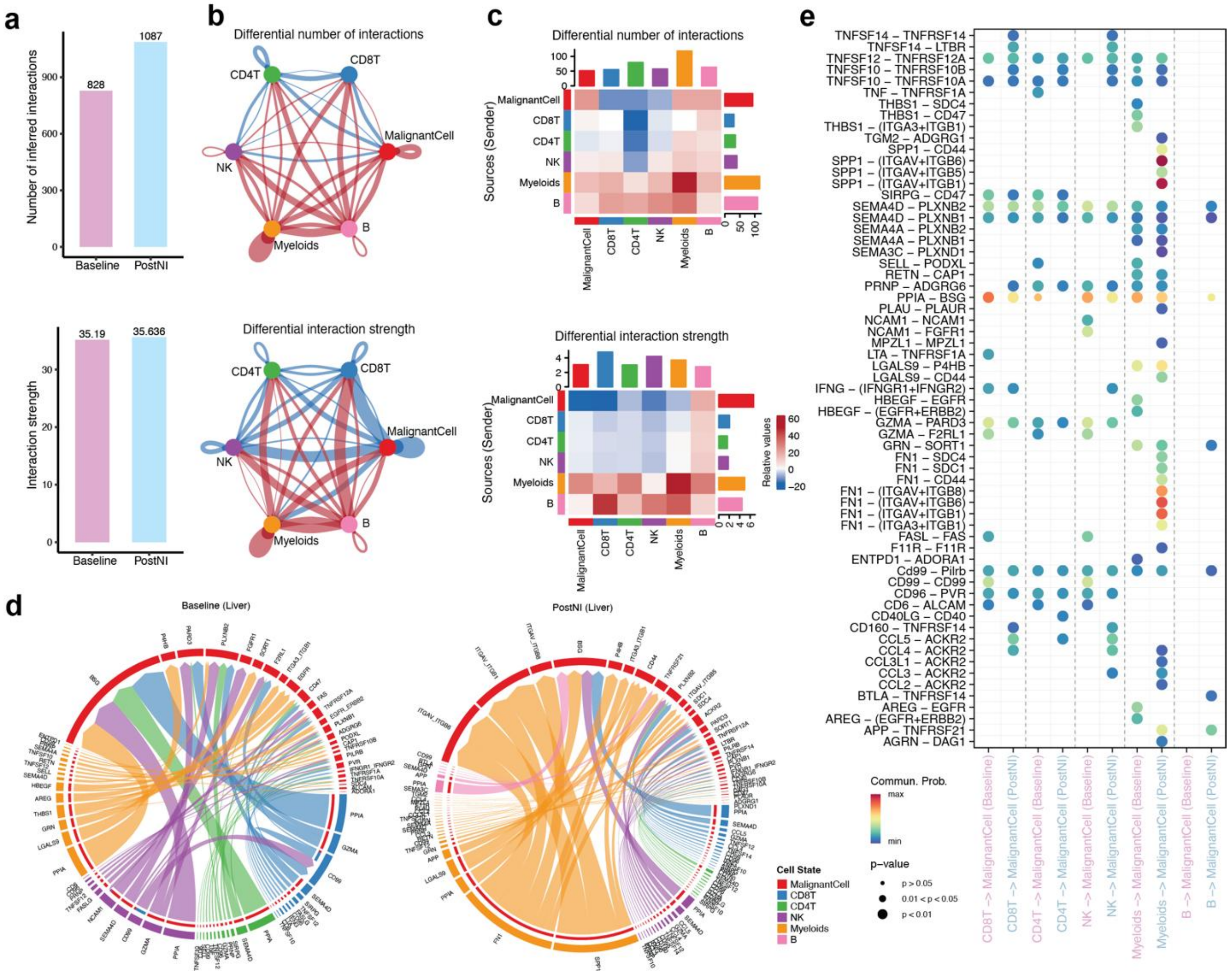

Supplementary Figure S19. Differential cell-cell communications between PostNI and baseline in liver metastasis (Baseline n = 1 and PostNI n = 2). (**a**) Bar plot shows the number of interactions and interaction strength of baseline and PostNI. **(b)** Circle plot shows the differential number of interactions and differential interaction strength across immune and tumor cells between PostNI and baseline. The blue line represents a decreased number or strength, and the red line represents an increased number or strength. **(c)** Heatmap shows the differential number of interactions and differential interaction strength from source cells to target cells between PostNI and baseline. **(d)** The chord plot shows the overall interactions between immune cells as source cells and malignant tumor cells as target cells. The color of the crosstalk represents the source cell color. **(e)** Dotplot shows the specific differential ligand-receptor of interactions of immune cell and malignant cell between PostNI and baseline. Source data are provided as a Source Data file.

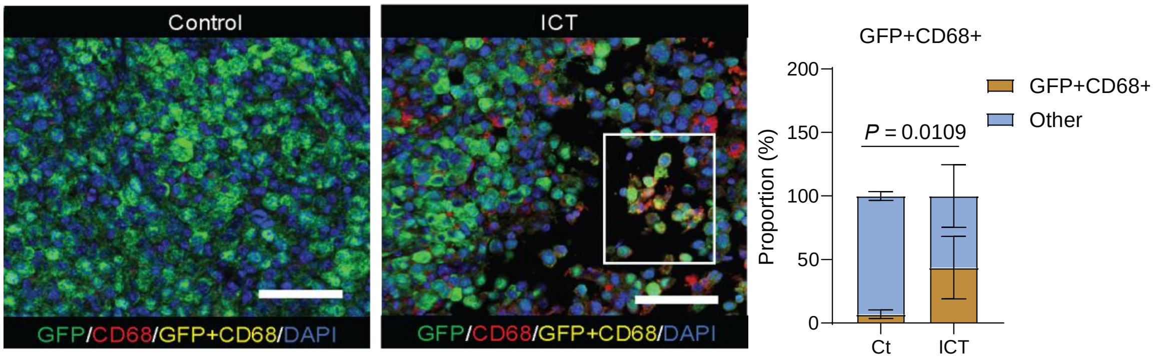

Supplementary Figure S20. Multiplex immunofluorescent analysis of CD68 in MSRT1 *ex vivo* tumors. (Left) Representative images of multiplex immunofluorescence analysis comparing RMC mouse models treated with IgG control (Ct, n = 6) compared to mice treated with anti-PD-1 plus anti-CTLA-4 (ICT, n = 6). Tumor cells are labeled with GFP. Scale bar is 100 µm. (Right) Quantification of the proportion of tumor cells labeled with GFP co-expressing CD68 in RMC mouse models treated with IgG control (Ct, n = 6 samples selected for analysis) compared to mice treated with anti-PD-1 plus anti-CTLA-4 (ICT, n = 6 samples selected for analysis). Data are expressed as mean value ± SD, with P value calculated by student's t test, and each replicate represents a biological replicate or mouse. Source data are provided as a Source Data file.

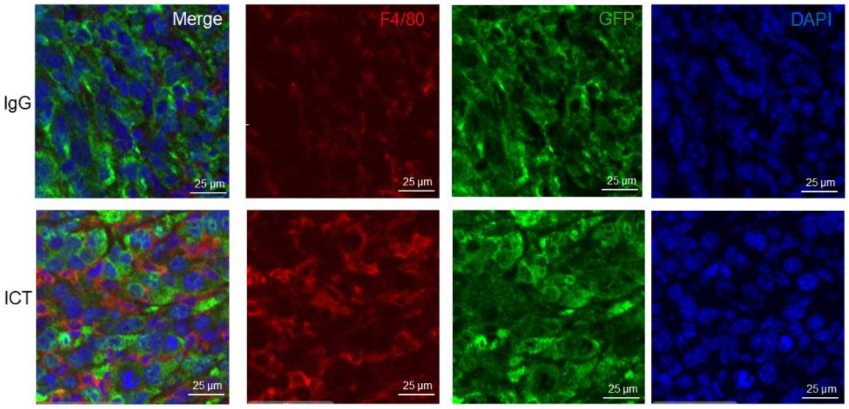

Supplementary Figure S21. Representative immunofluorescent images of F4/80, a mouse macrophage marker, co-expressed with GFP (tumor marker) in one representative *ex vivo* MSRT1 tumor from each cohort after 30 days of treatment with IgG or combination anti-PD1 and anti-CTLA-4 (ICT) *in vivo*. Scale bar is 25 µm. Images are 40X magnification.

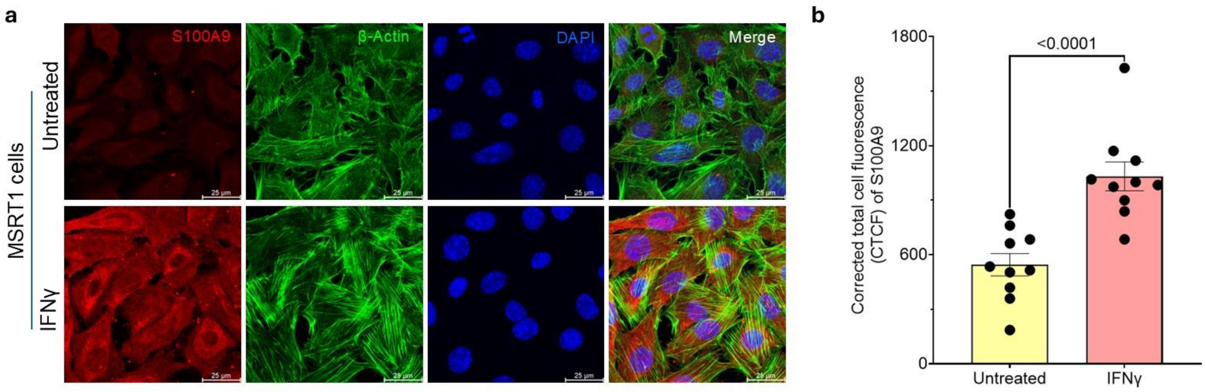

Supplementary Figure S22. S100A9 in MSRT1 cells. a. Representative immunofluorescent images of S100A9 in MSRT1 cells treated in vitro with interferon gamma (IFNγ) 100 ng/mL for 48 hours compared with untreated. Scale bar is 25 µm. Images are 40X magnification. **b.** Quantification of S100A9 in immunofluorescent study of MSRT1 cells treated with IFNγ 100 ng/mL for 48 hours compared with untreated. Each dot represents the number of fields of view of images taken (each field has approximately 50 cells). The experiments were replicated three times, and a representative experiment is shown. Data are expressed as mean value ± SD, with *P* value calculated by student's *t* test. Source data are provided as a Source Data file.

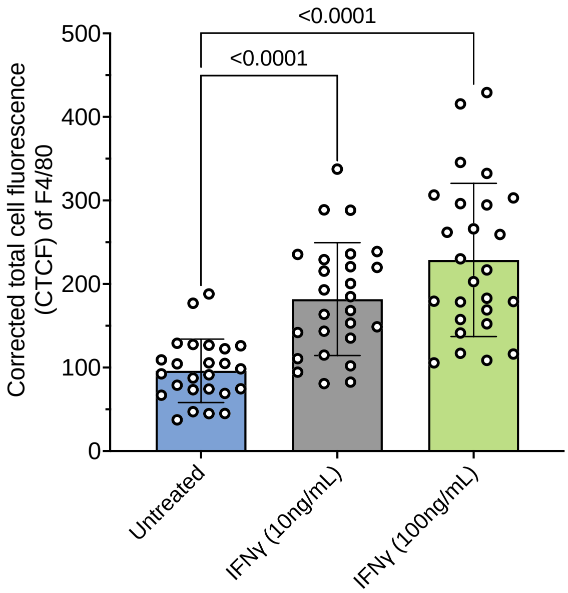

Supplementary Figure S23. Quantification of F4/80 in immunofluorescent study of MSRT1 cells treated with interferon gamma (IFNγ) and untreated for 48 hours. Increasing the concentration of IFNγ shows a linear increase in F4/80 protein expression based on immunofluorescence. Each dot represents the number of fields of view of images taken (each field has approximately 50 cells). The experiments were replicated three times, and a representative experiment is shown. Data are expressed as mean value ± SD, with *P* value calculated by student's *t* test. Source data are provided as a Source Data file.

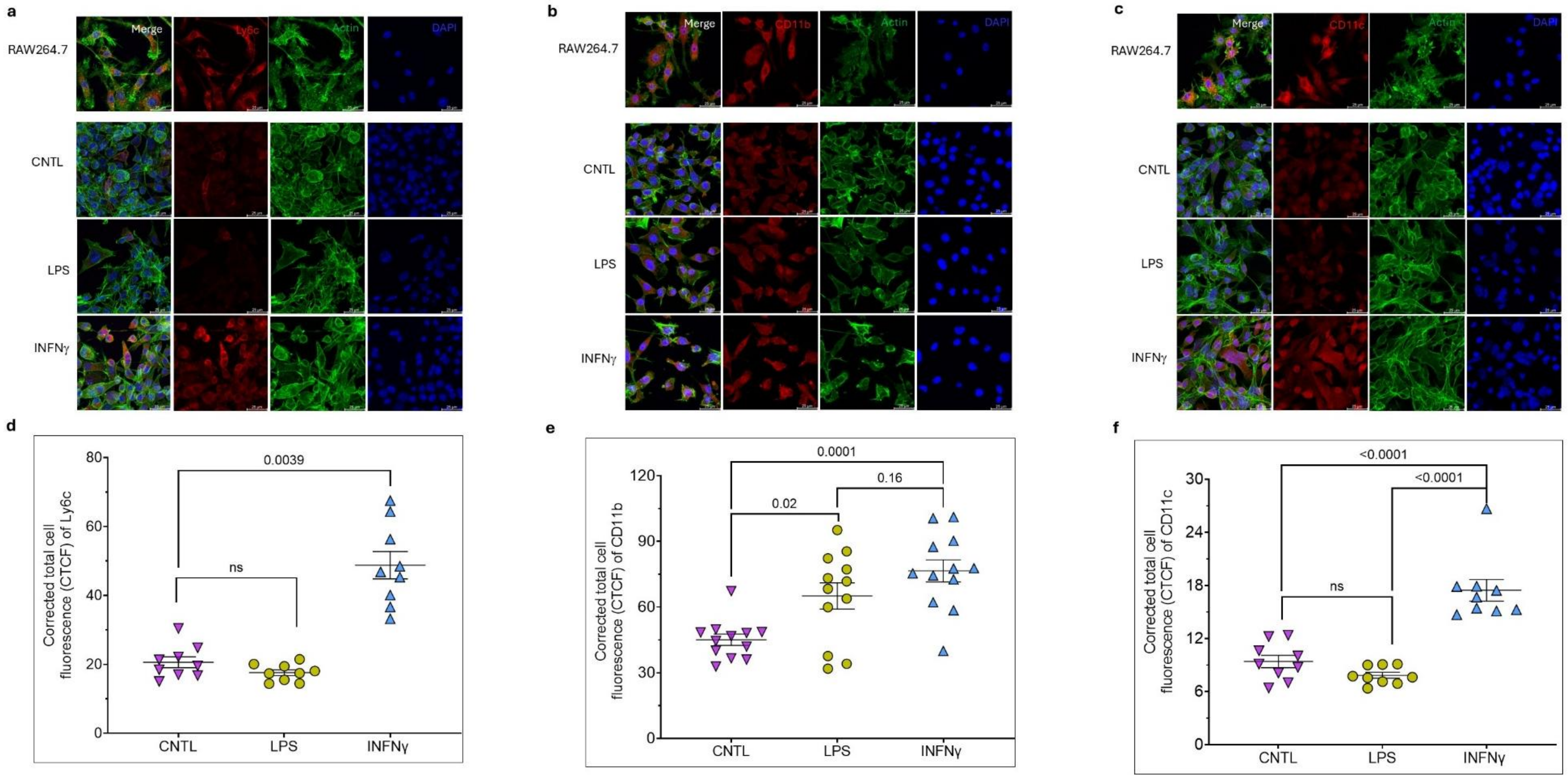

Supplementary Figure S24. Upregulation of mouse myeloid lineage markers in following in MSRT1 treated with IFNγ. (a-c) Representative immunofluorescent images of Ly6c **(a)**, CD11b **(b)**, and CD11c **(c)** in MSRT1 cells treated with interferon gamma (IFNγ) 100 ng/mL or lipopolysaccharides (LPS) 100 ng/mL *in vitro*. Untreated RAW264.7, a mouse macrophage-like cell line, was used as a positive control. Scale bars are 25 µm. **(d-e)** Quantification of Ly6c **(d)**, CD11b **(d)**, and CD11c **(e)** immunofluorescence in MSRT1 cells treated with IFNγ 100 ng/mL, lipopolysaccharides (LPS) 100 ng/mL, and untreated for 48 hours. The experiments were replicated three times, and a representative experiment is shown. Data are expressed as mean value ± SD, with *P* value calculated by student's *t* test. Source data are provided as a Source Data file.

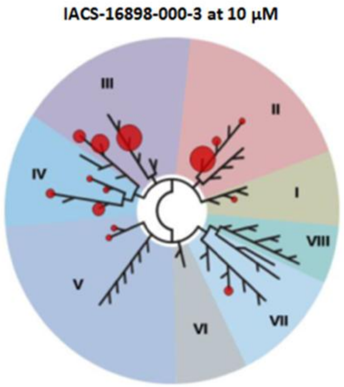

Supplementary Figure S25. BromoMAX results against 32 bromodomain containing proteins.

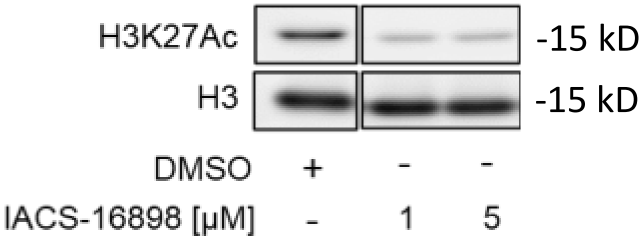

Supplementary Figure S26. Western blot analysis of two representative DOHH2 cell samples treated with IACS-16898 compared to one representative sample treated with DMSO as the control. Total histones were extracted, and total levels of histone H3 and global acetyl-H3K27 (K3K27Ac) were detected by western blot analysis. Cells were treated for 24 hours. The western blot membranes were scanned using the Li-Cor Odyssey instrument, producing digital scans without ladders. Molecular weight markers were, however, included on each membrane during electrophoresis and transfer, and were used to verify appropriate band migration and positioning prior to imaging.

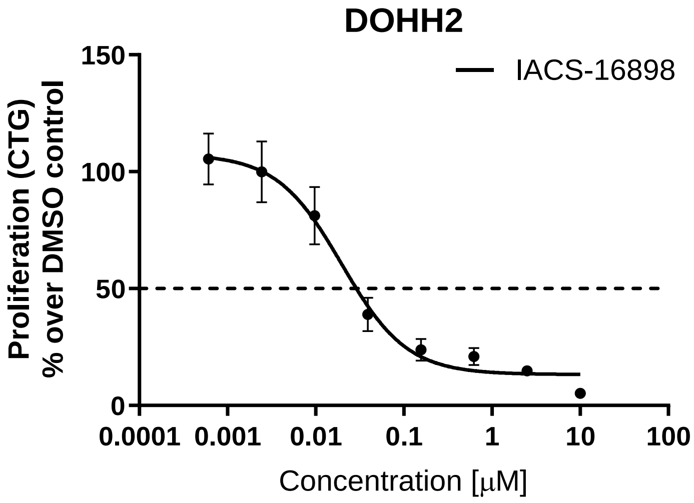

Supplementary Figure S27. Inhibition of DOHH2 cell proliferation by IACS-16898: Dose-response curve. Cell proliferation was analyzed by Cell Titer GLO assay. Results were normalized to vehicle control. Biological replicates of n = 6 were performed. Source data are provided as a Source Data file.

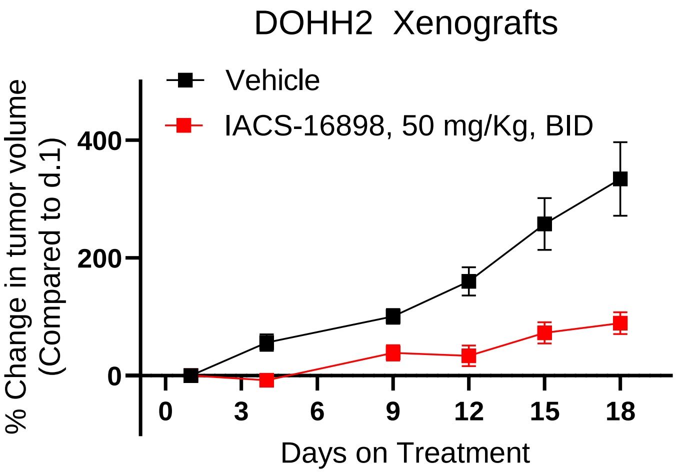

Supplementary Figure S28. IACS-16898 inhibits tumor growth in the DOHH2 subcutaneous xenograft model. Tumor burden was monitored by caliper measurements. ** p=0.002 as calculated by Student's t test compared to vehicle on day 18. Vehicle, n = 7 mice. IACS-16898, n = 7 mice. Source data are provided as a Source Data file.

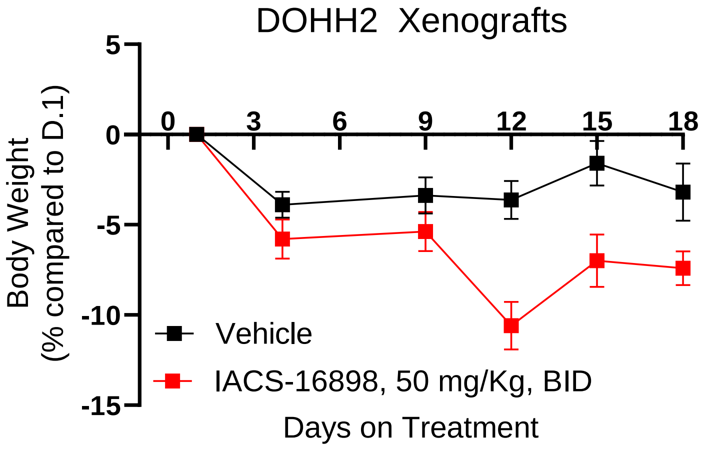

Supplementary Figure S29. Body weights as measured throughout the treatment with vehicle or IACS-16898. Vehicle, n = 7 mice. IACS-16898, n = 7 mice. Source data are provided as a Source Data file.

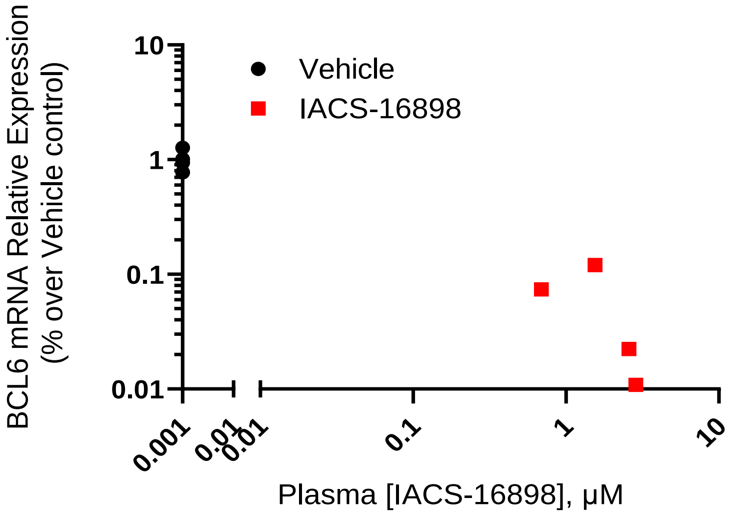

Supplementary Figure S30. Plasma PK was analyzed for correlation between exposure and BCL6 mRNA target inhibition measured by real-time PCR. N = 4 mice per treatment group. Statistical analysis was conducted by Student's t test ($p = 0.0001$). Source data are provided as a Source Data file.

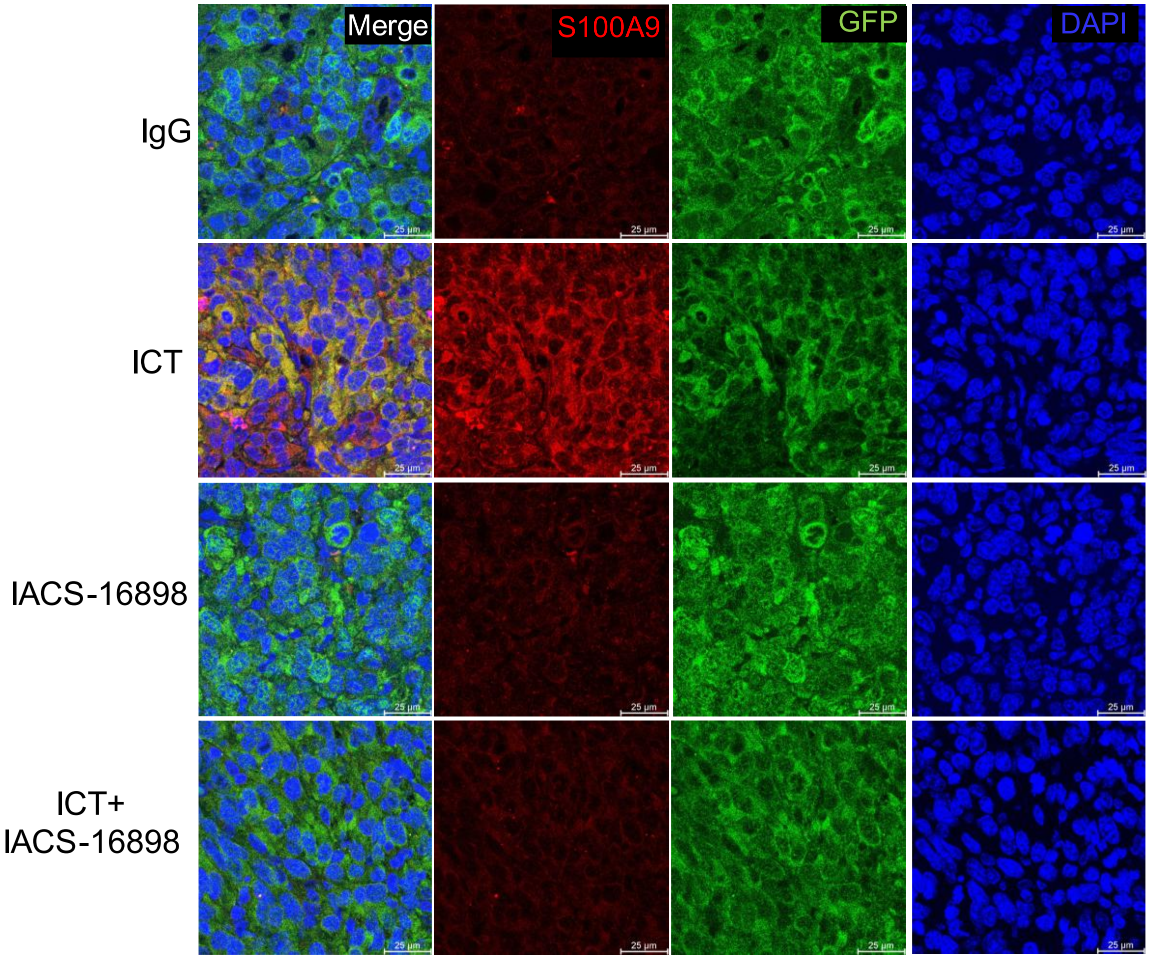

Supplementary Figure S31. Immunofluorescence of S100A9 in treated tumors. Representative images of immunofluorescence analysis comparing RMC mouse models treated with IgG (Control, n = 5) compared to mice treated with anti-PD-1 plus anti-CTLA-4 (ICT, n = 5), IACS-16898 (n = 5), and ICT plus IACS-16898 (n = 5). Tumor cells are labeled with GFP (green). S100A9 is shown in red. Yellow represents an overlap of S100A9 and GFP.

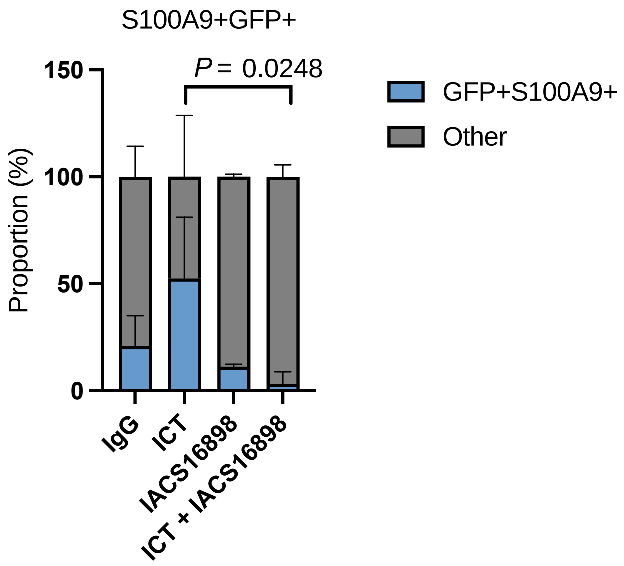

Supplementary Figure S32. Quantification of the proportion of tumor cells labeled with GFP co-expressing S100A9 in RMC mouse models treated with IgG control (n = 5) compared to mice treated with anti-PD-1 plus anti-CTLA-4 (ICT, n = 5), IACS16898 (n = 5), and ICT plus ICT (n = 5). Data are expressed as mean value ± SD, with *P* value calculated by student's *t* test, and graphing was done using Prism. Source data are provided as a Source Data file.

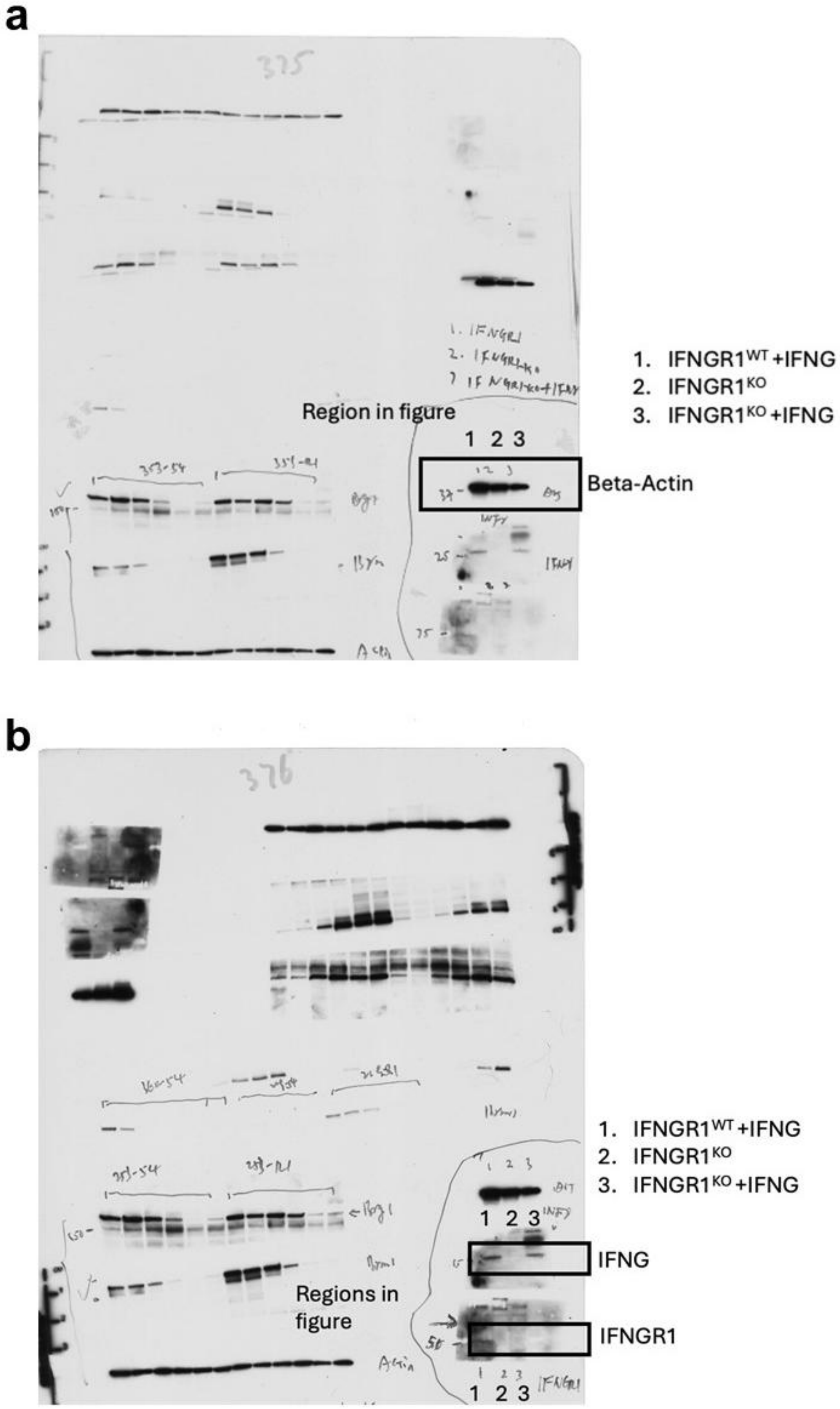

Supplementary Figure S33. Uncropped scanned films of western blots presented in Figure 6a. (a) Lower exposure film. **(b)** Higher exposure film.

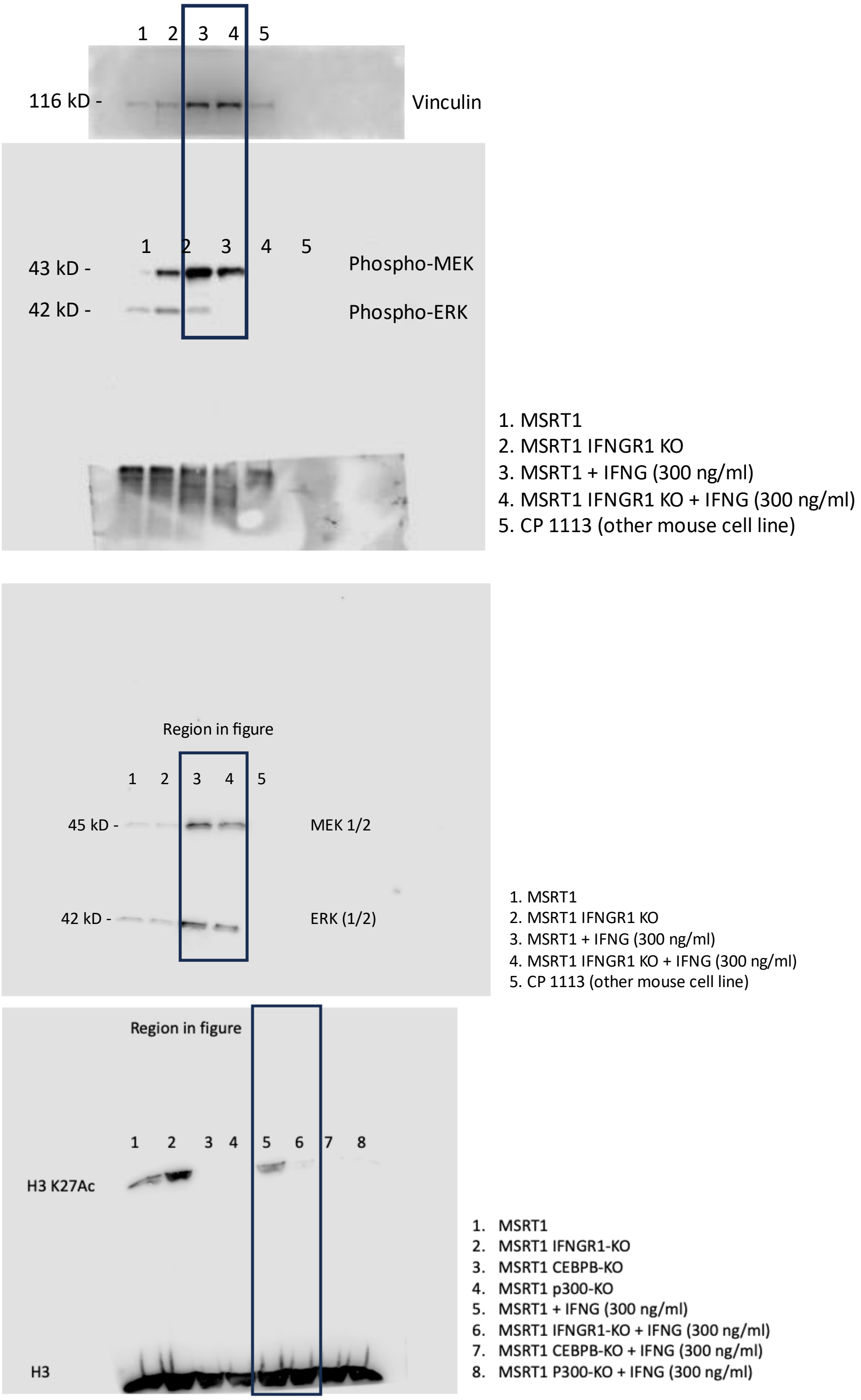

Supplementary Figure S34. Uncropped scanned films of western blots presented in Figure 6b. The western blot membranes were scanned using the Li-Cor Odyssey instrument, producing digital scans without ladders. Molecular weight markers were, however, included on each membrane during electrophoresis and transfer, and were used to verify appropriate band migration and positioning prior to imaging.

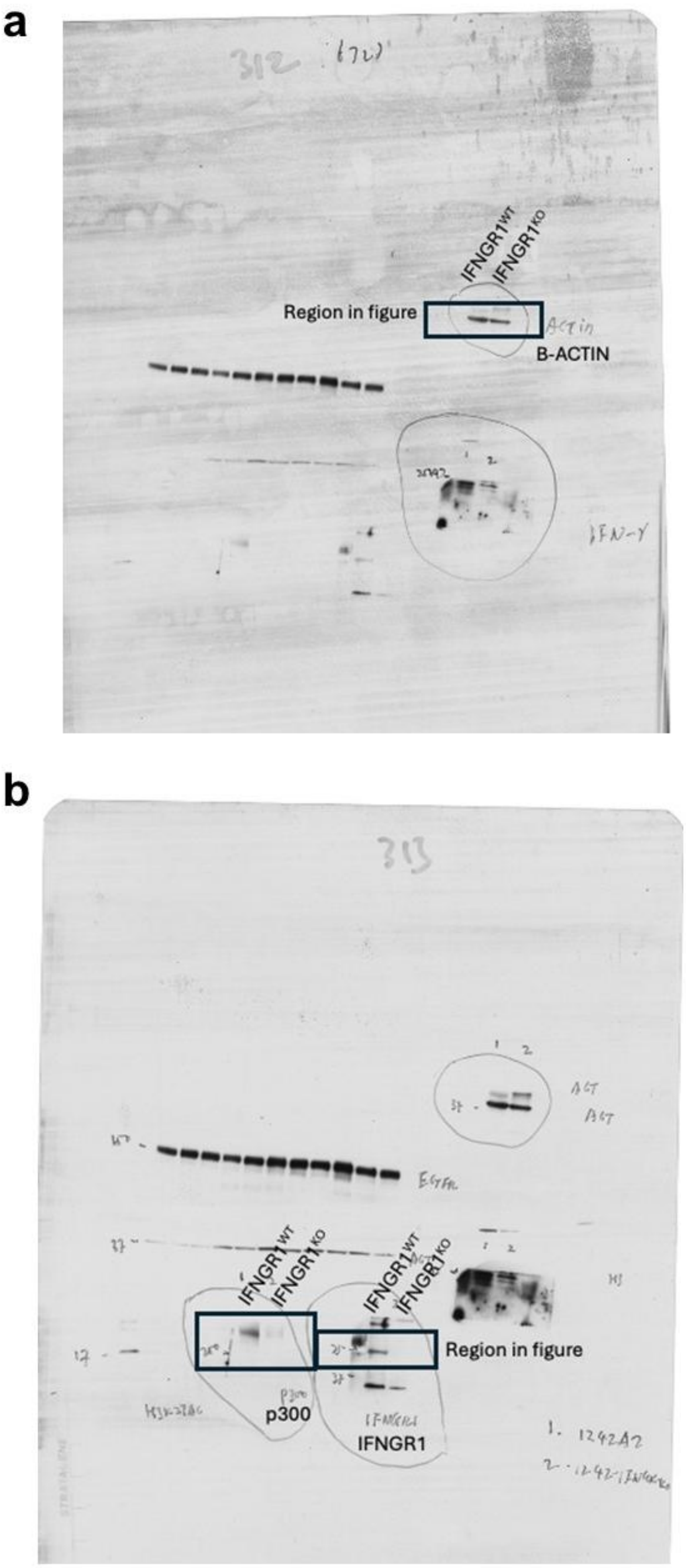

Supplementary Figure S35. Uncropped scanned films of western blots presented in Figure 7c. (a) Lower exposure film. **(b)** Higher exposure film.

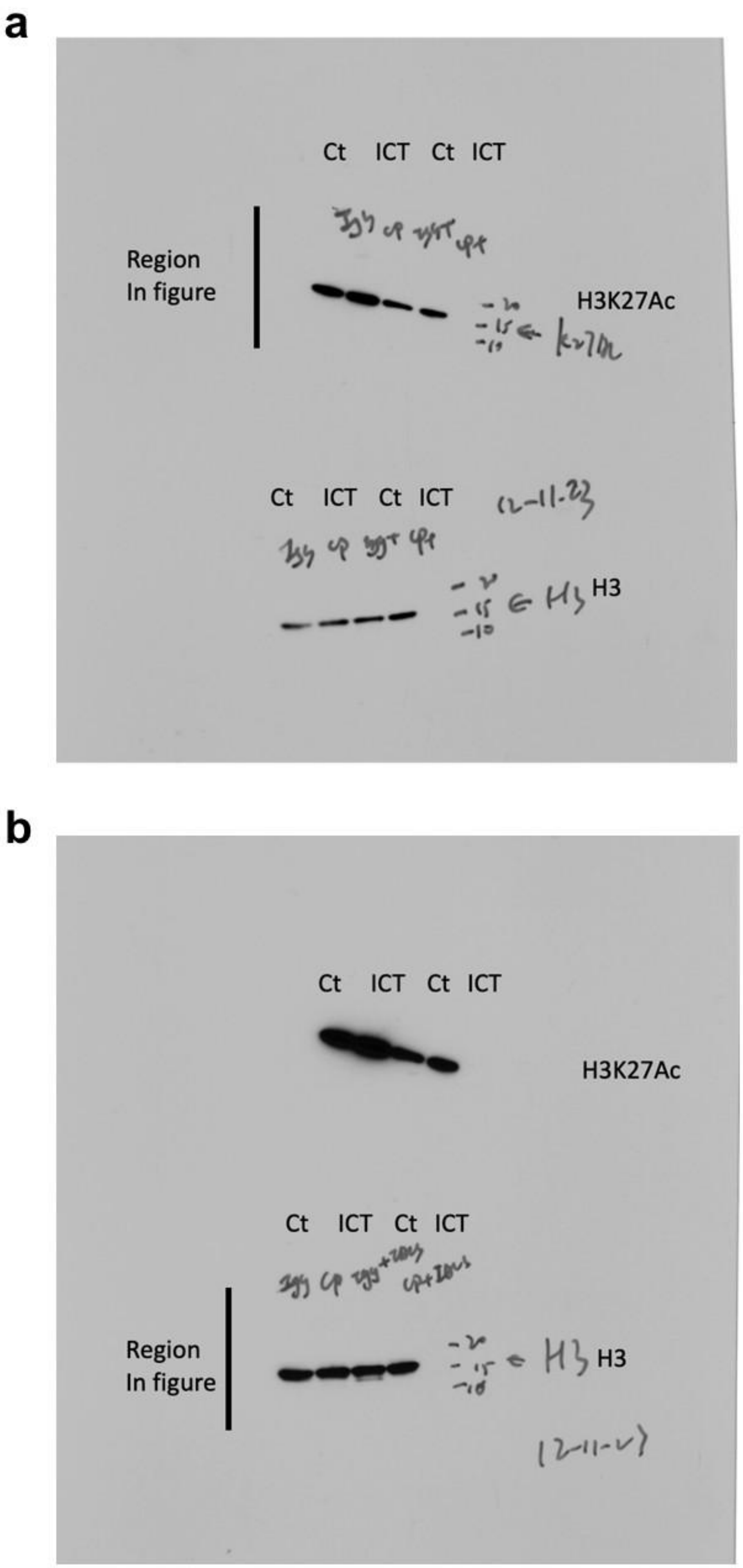

Supplementary Figure S36. Uncropped scanned films of western blots presented in Figure 8d. (a) Lower exposure film. **(b)** Higher exposure film.

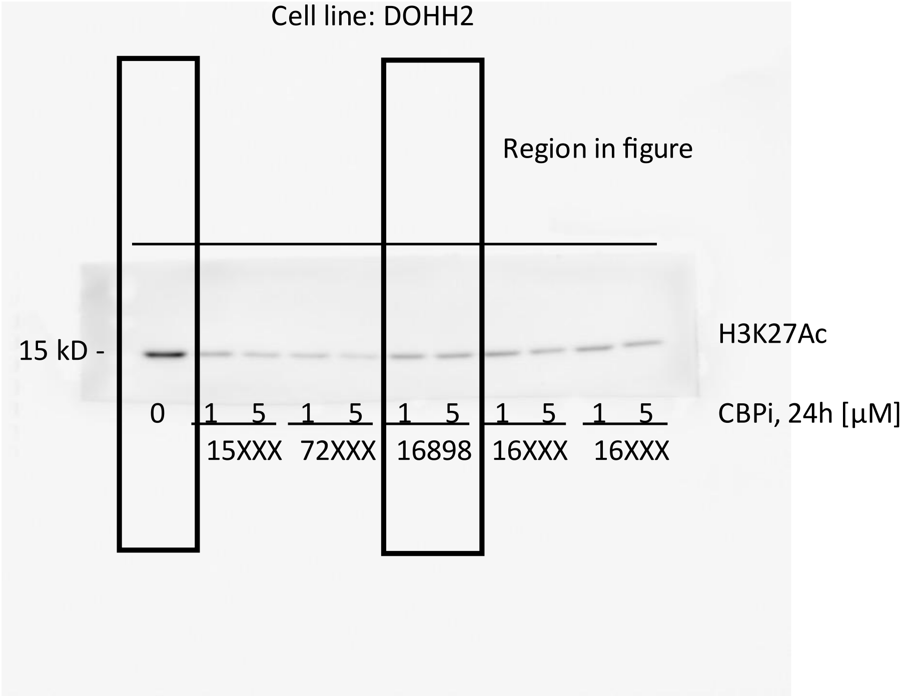

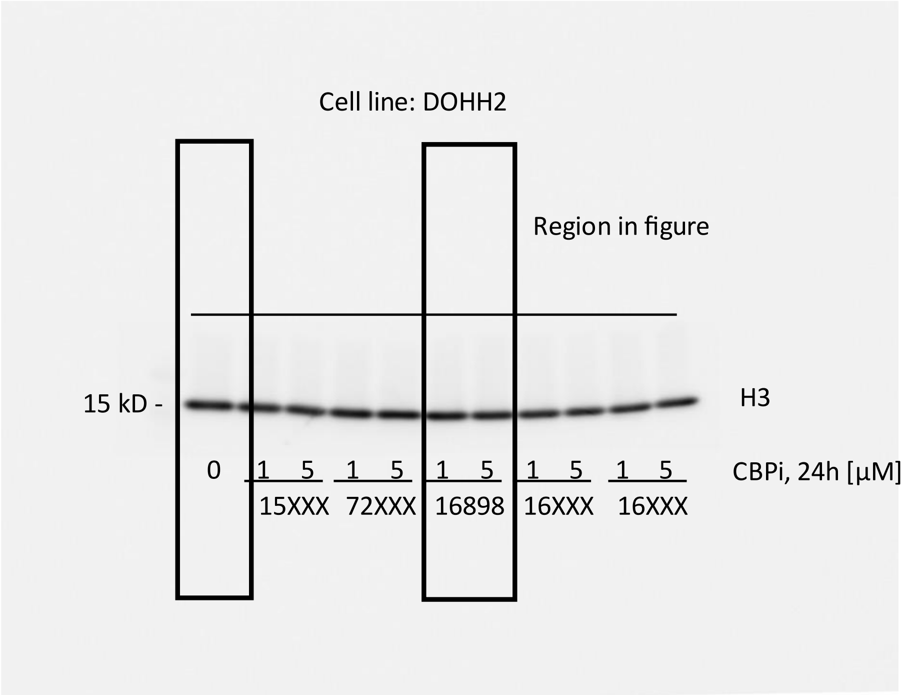

Supplementary Figure S37. Uncropped scanned films of western blots presented in Supplementary Figure S26. The western blot membranes were scanned using the Li-Cor Odyssey instrument, producing digital scans without ladders. Molecular weight markers were, however, included on each membrane during electrophoresis and transfer, and were used to verify appropriate band migration and positioning prior to imaging.Other compounds are de-identified with a series of “X’s” to protect intellectual property.

Supplementary Table S1. Treatment emergent adverse events at least possibly related to study therapy.

	Grade		
	1-2	≥3	Total
**Preferred AE Name**, n			
Alanine aminotransferase increased	1	1	2
Anemia	0	1	1
Hypotension	0	1	1
Pain	0	1	1
Neutrophil count decreased	3	0	3
Aspartate aminotransferase increased	2	0	2
Cortisol increased	2	0	2
Lipase increased	2	0	2
Serum amylase increased	2	0	2
White blood cell decreased	2	0	2
Constipation	1	0	1
Cortisol decreased	1	0	1
Cough	1	0	1
Fatigue	1	0	1
Lymphocyte count decreased	1	0	1
Nausea	1	0	1
Platelet count decreased	1	0	1
Proteinuria	1	0	1
T3 decreased	1	0	1
T4 increased	1	0	1
TSH decreased	1	0	1
Total protein increased	1	0	1

Supplementary Table S2. Treatment emergent adverse events regardless of attribution.

	Grade		
	1-2	≥3	Total
**Preferred AE Name**, n			
Pain	1	2	3
Abdominal pain	1	1	2
Alanine aminotransferase increased	1	1	2
Anemia	0	1	1
Hypotension	0	1	1
Pericardial tamponade	0	1	1
Back pain	4	0	4
Constipation	3	0	3
Dyspnea	3	0	3
Neutrophil count decreased	3	0	3
Proteinuria	3	0	3
Aspartate aminotransferase increased	2	0	2
Cortisol increased	2	0	2
Cough	2	0	2
Creatinine increased	2	0	2
Fatigue	2	0	2
Generalized muscle weakness	2	0	2
Hematuria	2	0	2
Lipase increased	2	0	2
Lymphocyte count decreased	2	0	2
Pleural effusion	2	0	2
Serum amylase increased	2	0	2
White blood cell decreased	2	0	2
Alkaline phosphatase increased	1	0	1
Allergic reaction	1	0	1
Allergic rhinitis	1	0	1
Anorexia	1	0	1
Blood LDH increased	1	0	1
Bronchial infection	1	0	1
Cortisol decreased	1	0	1
Fall	1	0	1
Gait disturbance	1	0	1
Hoarseness	1	0	1
Hypernatremia	1	0	1
Hypoalbuminemia	1	0	1
Insomnia	1	0	1
Nausea	1	0	1
Non-cardiac chest pain	1	0	1
Paresthesia	1	0	1
Platelet count decreased	1	0	1
T3 decreased	1	0	1
T4 increased	1	0	1
TSH decreased	1	0	1
Testosterone deficiency	1	0	1
Toe infection	1	0	1

Total protein increased	1	0	1
Weight loss	1	0	1

Supplementary Table S3. Patient samples used for single-cell RNA sequencing. Patients highlighted in red did not have tumor cells and were removed from the final analysis of scRNA-seq data.

Patient ID	Timepoint	Biopsy site	Treated on the clinical trial
RMC46	Baseline	Retroperitoneal lymph node	No
RMC49	Baseline	Retroperitoneal lymph node	No
RMC56	Baseline	Liver metastasis	No
RMC53	Baseline	Primary kidney tumor	Yes
RMC60	Baseline	Lung metastasis	Yes
RMC71	Baseline	Primary kidney tumor	No
RMC66	Baseline	Liver metastasis	No
RMC57	Post nivolumab + ipilimumab	Liver metastasis	No
RMC61	Post nivolumab + ipilimumab	Liver metastasis	Yes

Supplementary Table S4. Number of tumor cells in scRNA-seq analysis from the 7 patients with RMC listed in Supplementary Table S3.

	Kidney	Liver	Lymph Nodes	Total
**Baseline**	1333	319	910	2562
**PostNI**	0	634	0	634

Supplementary Table S5. Patient samples used for multiplex immunofluorescence.

Slide ID#	Sample ID	Treatment
1174154-1	Patient RMC25	Baseline
1182333-2	Patient RMC25	PostNI
1239492-1	Patient RMC29	Baseline
1269100-1	Patient RMC35	PostNI
1367225-1	Patient RMC47	Baseline
1395046-3	Patient RMC61	PostNI

Supplementary Table S6. Samples used for bulk RNA sequencing from patients enrolled in the clinical trial of nivolumab + ipilimumab.

Patient ID	Timepoint	Biopsy site
RMC25	Baseline	Lung metastasis
RMC35	Baseline	Lung metastasis
RMC47	Baseline	Abdominal soft tissue metastasis
RMC25	Post nivolumab + ipilimumab	Lung metastasis
RMC35	Post nivolumab + ipilimumab	Pleural metastasis
RMC44	Post nivolumab + ipilimumab	Retroperitoneal lymph node

Supplementary Table S7. Baseline demographics, clinical and prior treatment characteristics of the single-cell RNA sequencing patient cohort.

	Total<br>(N=7)
**Baseline Characteristics**	
**Age, Median (Range)**	36.0 (18.0, 57.0)
**Gender,** n (%)	
Female	2 (28.6%)
Male	5 (71.4%)
**Ethnicity,** n (%)	
Black or African American	6 (85.7%)
White or Caucasian	1 (14.3%)
**Sickle hemoglobinopathy,** n (%)	
Sickle cell trait	7 (100%)
**RMC laterality,** n (%)	
Right kidney	3 (42.9%)
Left kidney	4 (57.1%)
**ECOG performance Status,** n (%)	
0	2 (28.6%)
1	5 (71.4%)
**Stage at initial diagnosis of RMC,** n (%)	
III	1 (14.3%)
IV	6 (85.7%)
**Prior cytoreductive nephrectomy,** n (%)	
Yes	3 (42.9%)
No	4 (57.1%)
**Prior platinum-based chemotherapy,** n (%)	
Yes	6 (85.7%)
No	1 (14.3%)
**Number of prior systemic treatments, Median (Range)**	1 (0, 2)

Supplementary Table S8. BromoKdELECT data of IACS-16898. K_d values are expressed as nM.

**K_d (nM)**	**IACS-16898**
CREBBP	0.23
EP300	0.27
BRDT(1)	230
TAF1(2)	610
WDR9(2)	81
BAZ2A	3,300
BAZ2B	5,400
BRD1	410
BRD2(1)	2,600
BRD4(1)	980
BRD7	2,400
BRD9	1,500
BRPF1	730
FALZ	6,500

Supplementary Table S9. Selected BromoKdELECT data of IACS-16898. K_d values are expressed as nM.

**Target**	**IACS-16898 [nM]**
CBP K_d	0.23
BRD4 K_d	980

Supplementary Table S10. Pharmacokinetic exposure of IACS-16898. Mice (n = 3 mice per timepoint for a total of n = 18) were treated for 5 days, plasma was collected at indicated times and quantified (AUC_{last}: area under the curve from the time of dosing to the last measurable concentration; AUC_{INF_obs} extrapolated exposure to the infinite time; T_{max}: time point of maximum exposure; C_{max}: maximum concentration).

**Dose route**	**Dose (mg/ Kg)**	**Timepoint (Hr)**	**IACS-16898**
			**Mean Conc. (µM)**
PO	50	0	0.832
		0.5	171
		1	177
		2	118
		8	42.9
		16	0.667
**PK parameters**	**Unit**		
Tmax	Hr		1.00
Cmax	µM		177
AUClast	Hr * µM		802
AUCINF_obs	Hr * µM		804

Supplementary Table S11. *In vivo* pharmacokinetic properties of IACS-16898 in mouse, rat, dog and monkey**.** Clearance rate (Cl), distribution volume (Vdss), terminal half-life through IV dosing (T1/2), and bioavailability (F%) were calculated following standard formula. N=3 animals for rat, dog and monkey; mouse PK was performed with composite sampling with three timepoints collected per animal (total of 9 animals for study) IV doses were 1 mg/kg; PO doses were 10 mg/kg for mouse and 3 mg/kg for rat, dog and monkey. * IV and PO PK studies were performed separately.

	**Mouse**	**Rat**	**Dog**	**Monkey**
**Cl (mL/min/kg)**	5.0	8.9	6.7	12.3
**V_{dss}(L/kg)**	0.5	0.9	1.6	1.1
**$T_{1/2}$(hr)**	4.0	3.0	3.4	1.7
**%F**	>100	25	79*	29*

Supplementary Table S12. Stopping criteria for excessive TOX or insufficient responses.

Cohort Size	10	20	30*
Stop if there are this many patients (or fewer) with an objective response:	1	3	6
Stop if there are this many (or more) patients with TOX :	6	10	14

*Always stop with 30 patients, but if 6 or fewer patients respond or 14 or more have TOX, then this combination is not interesting for further investigation in these patients.

Supplementary Table S13. Operating characteristics under varying toxicity and objectiverResponse rates.

True Overall Toxicity Rate	True Objective Response Rate	Probability of Stopping Early	Probability of continuing after 10 patients	Median (25th %ile, 75th %ile)
0.10	0.10	0.90	0.26	10 (10, 20)
	0.20	0.51	0.62	20 (10, 30)
	0.30	0.19	0.85	30 (30, 30)
	0.40	0.05	0.95	30 (30, 30)
	0.50	0.01	0.99	30 (30, 30)
0.20	0.10	0.90	0.26	10 (10, 20)
	0.20	0.51	0.62	20 (10, 30)
	0.30	0.20	0.85	30 (30, 30)
	0.40	0.06	0.95	30 (30, 30)
	0.50	0.01	0.98	30 (30, 30)
**0.30**	0.10	0.91	0.25	10 (10, 20)
	0.20	0.55	0.59	20 (10, 30)
	**0.30**	**0.25**	**0.81**	**30 (20, 30)**
	0.40	0.12	0.91	30 (30, 30)
	0.50	0.09	0.94	30 (30, 30)
0.40	0.10	0.93	0.22	10 (10, 10)
	0.20	0.66	0.52	20 (10, 30)
	0.30	0.43	0.71	30 (10, 30)
	0.40	0.33	0.80	30 (20, 30)
	0.50	0.30	0.82	30 (20, 30)
0.50	0.10	0.96	0.16	10 (10, 10)
	0.20	0.82	0.39	10 (10, 20)
	0.30	0.70	0.53	20 (10, 30)
	0.40	0.65	0.59	20 (10, 30)
	0.50	0.63	0.62	20 (10, 30)

Supplementary Table S14. Panel of anti-human antibodies for CyTOF (mass cytometry).

Metal Tag	Marker	Clone	Staining step	Company	Catalog number	Conjugation	Working dilution
89Y	CD45	HI30	Surface	Standard BioTools	3089003B		1:100
141Pr	CD196 (CCR6)	G034E3	Surface	Standard BioTools	3141003A		1:800
142Nd	CD134(OX40)	ACT35	Surface	Standard BioTools	3142018B		1:100
143Nd	CD123 (IL-3R)	6H6	Surface	Standard BioTools	3143014B		1:200
144Nd	CD8a	RPA-T8	Surface	Biolegend	301002	Customized	1:10000
145Nd	CD4	RPA-T4	Surface	Standard BioTools	3145001B		1:200
146Nd	IgD	IA6-2	Surface	Standard BioTools	3146005B		1:800
147Sm	CD11c	Bu15	Surface	Standard BioTools	3147008B		1:400
148Nd	Ki67	Ki67	ICS	Biolegend	350523	Customized	1:400
149Sm	CD45RO	UCHL1	Surface	Standard BioTools	3149001B		1:100
150Nd	CD27	LG.3A10	Surface	Standard BioTools	3150017B		1:200
151Eu	TOX	6E6D03	ICS	Biolegend	682602	Customized	1:100
152Sm	TCRgd	11F2	Surface	Standard BioTools	3152008B		1:100
153Eu	CD45RA	HI100	Surface	Standard BioTools	3155011B		1:100
154Sm	CD185 (CXCR5)	J252D4	Surface	Biolegend	356902	Customized	1:100
155Gd	CD25	24212	Surface	Novus Biologicals	MAB1020	Customized	1:1600
156Gd	CD183 (CXCR3)	G025H7	Surface	Standard BioTools	3156004B		1:200
158Gd	CD194/CCR4	L291H4	Surface	Standard BioTools	3158032A		1:100
159Tb	TIGIT	MBSA43	Surface	Standard BioTools	3159038B		1:100
160Gd	CD28	CD28.2	Surface	Standard BioTools	3160003B		1:200
161Dy	CD38	HB-7	Surface	Biolegend	356602	Customized	1:3200
162Dy	CD69	FN50	Surface	Standard BioTools	3165042B		1:400
163Dy	CD56 (NCAM)	NCAM16.2	Surface	Standard BioTools	3163007B		1:100
164Dy	TCF1	1D2	ICS	Novus Biologicals	H00006932-M01	Customized	1:400
165Ho	CD349(PD1)	EH12.2H7	Surface	Standard BioTools	3165042B		1:100
166Er	CD14	63D3	Surface	Biolegend	367102	Customized	1:800

167Er	CD197 (CCR7)	G043H7	Surface	Standard BioTools	3167009A		1:200
168Er	Lag3	11C3C65	Surface	Biolegend	369302	Customized	1:800
169Tm	CD366(TIM3)	F38-2E8)	Surface	Standard BioTools	3169028B		1:100
170Er	CD3	UCHT1	Surface	Standard BioTools	3170001B		1:400
171Yb	CD20	2H7	Surface	Standard BioTools	3171012B		1:400
172Yb	CD33	WM53	Surface	Biolegend	303402	Customized	1:1600
173Yb	HLA-DR	L243	Surface	Standard BioTools	3173005B		1:200
174Yb	CD16	3G8	Surface	Biolegend	302051	Customized	1:1600
175Lu	CD278(ICOS)	C398.4A)	Surface	Standard BioTools	3175039B		1:100
176Yb	CD127 (IL-7Ra)	A019D5	Surface	Standard BioTools	3176004B		1:100
195Pt	CD19	HIB19	Surface	Biolegend	302202	Customized	1:100
196Pt	CD57	NK1	Surface	Biolegend	359602	Customized	1:10000
198Pt	Live/Dead			Standard BioTools	201198		2.5 µM
209Bi	CD137(4-1BB)	4B4-1	Surface	Standard BioTools	3209015B		1:100

Supplementary Table S15. Antibodies used for immunohistochemistry (IHC), and Western blot protein analysis (WB).

Antibody	Company	Catalog number	Host species	Dilution	Use	Tissue type in study
Acetyl-Histone H3 (Lys27) (D5E4) XP®	Cell Signaling Technology	8173	Rabbit	1:1000 (WB)	WB	mouse
CD3	Cell Signaling Technology	99940S	Rabbit monoclonal	1:100	mIF	mouse
CD68	Abcam	ab125212	Rabbit polyclonal	1:100	mIF	mouse
CD68	ThermoFisher	14-0688-82	Mouse monoclonal	1:500	mIF	mouse
GFP (D5.1)	Cell Signaling Technology	2956	Rabbit	1:1000(WB); 1:200 (IHC)	IHC, WB	mouse
Histone H3	Cell Signaling Technology	9715	Rabbit	1:1000 (WB)	WB	mouse
IFNGR1	Proteintech	10808-1-AP	Rabbit polyclonal	1:200 (mIF), 1:1000 (WB)	mIF, WB	human
IFNGR1	R&D systems	MAB-10261	Hamster monoclonal	1:300-1:400	mIF	mouse
Phospho-MEK1/2 (Ser217/221)	Cell Signaling Technology	9121	Rabbit	1:1000(WB); 1:200 (IHC)	IHC, WB	mouse
Phospho-p44/42 MAPK (Erk1/2) (Thr202/Tyr204)	Cell Signaling Technology	9101	Rabbit	1:1000(WB); 1:200 (IHC)	IHC, WB	mouse

Supplementary Table S16. Panel antibody optimization by multiplex immunofluorescence using Opal fluorophores (Akoya Biosciences) for automated staining using Leica Bond RX.

Panel	Antibody (Ab)	Clone	Vendor	AR	Ab. Dilution	F	F. Dilution
1	panCK	AE1/AE3	Dako	pH6	1:100	650	1:150
	CD3	D7A6E(AM)	Cell Signaling	pH6	1:100	780D	1:100
	CD8	C8/144B	Thermo	pH6	1:25	520	1:100
	PD-1	EPR4877(2)	Abcam	pH9	1:250	620	1:100
	PD-L1	E1L3N	Cell Signaling	pH6	1:1500	690	1:150
	FOXP3	D2W8E	Cell Signaling	pH6	1:50	570	1:100
	KI67	MIB-1	Dako	pH9	1:100	480	1:100
	CD68	PG-M1	Dako	pH6	1:50	540	1:100
2	panCK	AE1/AE3	Dako	pH6	1:100	650	1:150
	CD3	D7A6E(AM)	Cell Signaling	pH9	1:100	480	1:100
	CD8	C8/144B	Thermo	pH6	1:25	520	1:100
	PD-1	EPR4877(2)	Abcam	pH9	1:250	620	1:100
	PD-L1	E1L3N	Cell Signaling	pH6	1:1500	690	1:150
	FOXP3	D2W8E	Cell Signaling	pH6	1:50	570	1:100
	CD20	L26	Dako	pH6	1:50	780D	1:100
	CD68	PG-M1	Dako	pH6	1:50	540	1:100
3	panCK	AE1/AE3	Dako	pH6	1:50	690	1:150
	CD3	Polyclonal	Dako	pH6	1:200	690	1:100
	ICOS	D1K2T	Cell signaling	pH6	1:200	520	1:150
	LAG3	D2G40	Cell signaling	pH6	1:200	650	1:200
	TIM3	D5D5R	Cell signaling	pH6	1:100	540	1:100
	VISTA	D1L2G	Cell signaling	pH9	1:400	620	1:100
	OX40	ACT 35	e-biosystem	pH6	1:10	570	1:100
4	panCK	AE1/AE3	Dako	pH6	1:25	540	1:100
	CD68	PG-M1	Dako	pH9	1:25	520	1:100
	MRP8-14	S100A8/A9	OriGene	pH6	1:50	780D	1:100
	CD86	E2G8P	Cell Signaling	pH9	1:100	620	1:100
	CD206	Polyclonal	Invitrogen	pH6	1:100	570	1:100
	CD163	10D6	Leica	pH9	1:100	690	1:100
	Arginase-1	D4E3M	Cell Signaling	pH9	1:200	650	1:100
	PD-L1	E1L3N	Cell Signaling	pH6	1:100	480	1:100

AR, antigen retrieval; panCK, pancytokeratin; Ab, antibody dilution; F, fluorophore.

Supplementary Table S17. Panel antibody optimization by multiplex immunofluorescence using Opal fluorophores (Akoya Biosciences) for manual staining.

Panel	Antibody (Ab)	Clone	Vendor	AR	Ab. Dilution	F	F. Dilution
1	IFNGR1	10808-1-AP	Proteintech	pH9	1:200	520	1:200
	S100A9	ab63818	Abcam	pH6	1:100	570	1:200
	Keratin 19	10712-1-AP	Proteintech	pH9	1:400	690	1:200
	IFNG	15356-1-AP	Proteintech	pH6	1:1000	620	1:200
2	GFP	D5.1	Cell Signaling	pH6	1:200	780	1:200
	S100A9	PA1-46489	Invitrogen	pH6	1:100	620	1:200
3	GFP	D5.1	Cell Signaling	pH6	1:200	780	1:200
	CD68	Ab125212	Abcam	pH6	1:100	620	1:200

AR, antigen retrieval; Ab, antibody dilution; F, fluorophore.

Supplementary Note

Clinical Trial Protocol

The University of Texas

M.D. Anderson Cancer Center Division of Cancer Medicine

Phase II Trial of Nivolumab plus Ipilimumab in Patients with Renal Medullary Carcinoma

Lead Institution: The University of Texas M. D. Anderson Cancer Center

Principal Investigator:
Pavlos Msaouel MD, PhD
1155 Pressler Street, Unit 1374
Houston, TX 77030
Telephone: (713) 792-0067
Fax: (713) 745-1625
E-mail: pmsaouel@mdanderson.org

Co-Principal Investigator:
Nizar M. Tannir MD, FACP
1155 Pressler Street, Unit 1374
Houston, TX 77030
Telephone: (713) 792-2830
Fax: (713) 745-1625
E-mail: ntannir@mdanderson.org

Biostatistician:
Rebecca Slack Tidwell, MS
1400 Pressler Street, Unit 1411
Houston, TX 77030
Email: rsslack@mdanderson.org

Protocol version	Header date
V00 All documents CRC submission	04/17/2017
V01 IRB submission	05/15/2017
V02	06/16/2017
V03	09/11/2017
V04	09/11/2017
V05	09/11/2017
V06	10/13/2017
V07	12/06/2017
V08	05/24/2018
V09	08/01/2018

Table of Contents

Table of Contents 2

1.0 RESEARCH HYPOTHESIS 5

2.0 OBJECTIVES 5

3.0 BACKGROUND AND RATIONALE 5

4.0 ELIGIBILITY CRITERIA 15

5.0 TREATMENT PLAN 19

6.0 STUDY MEDICATIONS 23

7.0 CORRELATIVE STUDIES/TUMOR TISSUE COLLECTION 30

8.0 DISCONTINUATION OF THERAPY 30

9.0 PRE-TREATMENT EVALUATION 32

10.0 EVALUATION DURING TREATMENT 33

11.0 STUDY CALENDAR 36

12.0 CRITERIA FOR RESPONSE OR PROGRESSION 39

13.0 CRITERIA FOR REMOVAL FROM PROTOCOL TREATMENT 42

14.0 SAFETY ASSESSMENTS AND REPORTING REQUIREMENTS 43

15.0 STATISTICAL CONSIDERATIONS / DATA ANALYSIS 466

16.0 DATA AND PROTOCOL MANAGEMENT 49

17.0 REFERENCES 51

Appendix 1: Tests to Be Performed on MD Anderson Companion Trial 2014-0938 54

Table 1. LIST OF ABBREVIATIONS

Abbreviation	Term
AEs	Adverse Events
APC	Antigen-Presenting Cell
BMS	Bristol-Myers Squibb
BTLA	B- and T-cell Lymphocyte Attenuator
ccRCC	Clear-Cell Renal Cell Carcinoma
CI	Confidence Intervals
CrCl	Creatinine Clearance
CRF	Case Report Form
CTCAE	Common Terminology Criteria for Adverse Events
CTLA-4	Cytotoxic T Lymphocyte Antigen-4
ECOG	Eastern Cooperative Oncology Group
FDA	United States Food and Drug Administration
FFPE	Formalin-Fixed Paraffin-Embedded
HBV	Hepatitis B Virus
HBVsAG	Hepatitis B Virus Surface Antigen
HCV	Hepatitis C Virus
ICF	Informed Consent Form
irAE	Immune-Related Adverse Event
irPD	Immune-Related Progressive Disease
irRECIST	Immune-Related Response Evaluation Criteria In Solid Tumors
IV	Intravenous
LAG-3	Lymphocyte Activation Gene-3
mAb	Monoclonal Antibody
mTOR	Mechanistic Target of Rapamycin
NSCLC	Non–Small Cell Lung Cancer
ORA	Office of Research Administration
ORR	Objective Response Rate
OS	Overall Survival
PD	Progressive Disease
PD-1	Programmed Death Receptor-1
PD-L1	Programmed Death-ligand 1
PD-L2	Programmed Death-ligand 2
PFS	Progression-Free Survival
PK	Pharmacokinetic
RCC	Renal Cell Carcinoma
RECIST	Response Evaluation Criteria In Solid Tumors
RMC	Renal Medullary Carcinoma
RNA	Ribonucleic Acid
SAE	Serious Adverse Event

SCT	Sickle Cell Trait
SD	Stable Disease
TIM-3	T Cell Immunoglobulin and Mucin Protein-3
Tmax	Time of Maximum concentration observed
TMTB	Total Measured Tumor Burden
ULN	Upper Limit of Normal
VISTA	V-domain Ig Suppressor of T-cell Activation
WOCBP	Women of Childbearing Potential

1.0 RESEARCH HYPOTHESIS

Combined immune checkpoint therapy using nivolumab plus ipilimumab will produce a potent and durable antitumor response that will improve the objective response rate (ORR) and survival outcomes of patients with locally advanced or metastatic renal medullary carcinoma (RMC).

2.0 OBJECTIVES

Primary objective:

- To determine the objective response rate (ORR) of patients with locally advanced or metastatic RMC treated with combination of nivolumab plus ipilimumab. ORR is defined as the proportion of patients with a best response of complete response (CR) or partial response (PR) by the Response Evaluation Criteria. In Solid Tumors (RECIST 1.1) criteria recorded between Day 1 of the study and the date of objectively documented progression per RECIST 1.1 or the date of subsequent anti-cancer therapy, whichever occurs first. Our goal is to significantly improve the ORR compared with the historical ORR of 29% achieved in our institution using conventional cytotoxic chemotherapies.

Secondary objectives:

- To determine the efficacy and safety of the combination of nivolumab plus ipilimumab in patients with RMC. Efficacy will be measured by overall survival (OS), progression-free survival (PFS), time to ORR, duration of response, and the disease control rate (DCR).
- To evaluate potential biomarkers for patient stratification and treatment response, as well as tumor antigen-specific immune responses, such as antibody and T cell responses, as surrogates for anti-tumor activity.

3.0 BACKGROUND AND RATIONALE

3.1 Introduction

Originally described in 1995,[1] renal medullary carcinoma (RMC) predominantly afflicts young adults and adolescents with sickle cell trait (SCT), and is one of the most aggressive renal cell carcinomas.[2,3] In the original series by Davis et al,[1] the median OS of patients with RMC was only 4 months, and this has only improved to 13 months despite therapy in the most recent series of cases.[2] RMC is a rare tumor that comprises <0.5% of all renal cell carcinomas[4], but its incidence is likely underestimated as it is a challenging diagnosis that can often be mistaken for collecting duct carcinoma or other aggressive kidney malignancies.[5]

Similarly to other renal malignancies such as clear cell renal cell carcinoma and collecting duct carcinoma,[6-8] men are twice as likely to be affected by RMC than women.[2,9] Afflicted patients have a median age of 28 years (range 9-48 years) and most patients (~67%) will present with metastatic disease, primarily to the lymph nodes (85% of cases), lungs (46%), liver (15%), and bone (15%).[2] Metastases to the central nervous system are extremely rare (<1% of cases),[2,9]

suggesting a low predilection of the disease to the brain parenchyma. Approximately 27% of patients with metastatic disease will have 1-2 metastatic sites, whereas 73% of patients will have more than 2 sites of metastatic involvement.[2]

For reasons that remain to be elucidated, the vast majority of patients with RMC have SCT,[2,9] while only a handful of cases have been documented in patients with homozygous sickle cell disease,[9-11] hemoglobin SC disease,[9,10] or sickle beta thalassemia.[2,9] Approximately 1 in 14 African-Americans have SCT,[12] and between 1/20,000 to 1/39,000 will develop RMC.[9] Furthermore, SCT is found in 300 million individuals worldwide,[13] with prevalence rates varying from ~7% among African-Americans,[12] 7.5% in Greeks,14 4-5% in Turkey,[15] up to 13% in some Indian populations,16 20% in the eastern province of Saudi Arabia,[17] and between 10%-40% across equatorial Africa, reaching up to 45% in certain regions of Uganda.[18]

Renal medullary carcinoma is characterized by complete loss of expression of the SMARCB1 protein, also known as INI1, hSNF5, or BAF47.[19,20] Histologically, RMC presents as a high-grade, poorly differentiated adenocarcinoma containing focal anastomosing tubules and cords with a reticular and cribriform appearance, as well as a myxoid highly desmoplastic stroma with neutrophil infiltrates and microabscess-like foci.[1,5] Sickle red blood cells in the tumor specimen confirm the diagnosis. Immunohistochemistry demonstrates loss of SMARCB1, and, in many cases, expression of the stem cell marker OCT3/4.[21] Immunohistochemical evidence of SMARCB1 expression excludes the diagnosis of RMC. Computed tomography (CT) imaging at presentation will demonstrate an ill-defined heterogeneous mass, arising from the renal medulla, more frequently in the right kidney, with intratumoral necrosis, an average size of 6-7 centimeters,[2] and lower contrast enhancement than the renal cortex and medulla during all phases.[22]

3.1.1 Current management of RMC

Localized RMC is preferably treated with nephrectomy and retroperitoneal lymph node dissection followed by close surveillance.[2] Radical nephrectomy is favored over partial nephrectomy even in very early stage tumors due to the medullary epicenter and infiltrative nature of RMC. In patients with metastatic disease, retrospective data suggest that cytoreductive nephrectomy, when feasible, results in improved overall survival (16.4 months vs 7.0 months) compared with systemic chemotherapy alone regardless of Eastern Cooperative Oncology Group (ECOG) performance status (0-1 or 2-3) or whether systemic chemotherapy is first administered pre-operatively or after nephrectomy.[2] Based on these results, as well as expert opinion, it is currently recommended that patients with metastatic RMC and ECOG performance status of 0-1 undergo upfront cytoreductive nephrectomy with retroperitoneal lymph node dissection, particularly if this will remove most of the tumor burden, followed by systemic chemotherapy. Upfront systemic therapy is preferred for patients who present with ECOG performance status of 2-3, and/or heavy metastatic disease burden outside the primary tumor. Cytoreductive nephrectomy with retroperitoneal lymph node dissection can be performed later, if there is a good response to systemic chemotherapy. Distant metastasectomy is generally not recommended.

RMC is refractory to targeted anti-angiogenic therapies, such as sorafenib, sunitinib, pazopanib, axitinib, and bevacizumab, or mechanistic target of rapamycin (mTOR) inhibitors such as everolimus and temsirolimus that are used against other renal cell carcinomas.[2] Cytotoxic combination chemotherapy is the only systemic treatment approach that has consistently shown to produce partial or complete responses in approximately 29% of cases.[2] Thus, outside of clinical

trials, cytotoxic combination chemotherapy remains the mainstay of systemic treatment for RMC. Unfortunately, responses are not durable in most cases and very few patients will survive for >24 months.[2] Novel therapeutic strategies are therefore urgently needed.

3.2 Background on Nivolumab (Opdivo)

3.2.1 Mechanism of Action

Immune checkpoints are regulatory signals that can affect immune activation and self-tolerance. Immune checkpoint signaling is crucial for preventing autoimmunity and for protecting host tissues from immune-mediated collateral damage. Checkpoint inhibitory molecules including cytotoxic T lymphocyte antigen-4 (CTLA-4), programmed death-1 (PD-1), lymphocyte activation gene-3 (LAG-3), T cell immunoglobulin and mucin protein-3 (TIM-3), B7-H3 (also designated as CD276), B- and T-cell lymphocyte attenuator (BTLA), and the V-domain Ig suppressor of T-cell activation (VISTA) are frequently expressed on tumor-infiltrating T cells.[23] Although the exact signaling pathways associated with these molecules remain to be fully elucidated, it is clear that they regulate immune activity by different mechanisms and at different levels.

PD-1 is the first immune checkpoint receptor to be targeted in clinical practice against metastatic clear-cell renal cell carcinoma (ccRCC).[24] In contrast to CTLA-4, which is near exclusively expressed on T cells, PD-1 is more broadly expressed and can limit the activity of both T- and B-lymphocytes, NK cells, and certain myeloid cells when bound to either of its two known ligands, PD-L1 and PD-L2.[23] PD-L1 and PD-L2 have distinct expression profiles.[25] PD-L1 is expressed not only on antigen-presenting cells (APCs), but also on non-hematopoietic cells, including tumor cells. Expression of PD-L2 is largely restricted to APCs including macrophages and myeloid dendritic cells, as well as mast cells. PD-L1 can be aberrantly produced in cancer tissues resulting in tumor-induced immune dampening via the PD-1 signaling pathway.[26] Therefore, whereas CTLA-4 regulation occurs mainly in lymphoid tissues, PD-1 is predominantly activated within the tumor microenvironment.

The role of PD-1 as a negative regulator of T cells was demonstrated in PD-1 deficient mice which developed significant autoimmunity with elevated titers of autoantibodies.[23,27] In addition, blocking antibodies against PD-1 activated immune responses that reduced tumor metastasis and tumor growth in a number of experimental tumor models.[28,29] Consistent with the immunosuppressive role of PD-1/PD-L1/2 signaling, forced expression of PD-L1 in murine tumor cell lines allows increased tumor growth *in vivo*, previously kept in check by T cells. This inciting effect of PD-L1 on tumor growth is reversed by blocking anti-PD-L1 antibodies.[30]

Nivolumab, a fully humanized IgG4 (kappa) isotype anti-PD-1 monoclonal antibody (mAb), was the first anti-PD-1 agent to be approved for clinical use. It has received accelerated approval by the United States Food and Drug Administration (FDA) for use in metastatic or unresectable melanoma, metastatic non-small cell lung cancer, and metastatic ccRCC based on its proven clinical efficacy in these settings.[24,31-34] Immune reactivation by nivolumab produces long-term responses that persist even after treatment cessation.[35]

3.2.2 Nivolumab Pharmacokinetics

The pharmacokinetics (PK) of single-dose nivolumab was assessed in patients with multiple tumor

types in the CA209001 trial, using a dose range of 0.3 to 10 mg/kg. The time of maximum concentration observed (T_{max}) across single dose levels ranged from 1.6-3.0 hours with individual values ranging from 0.9 to 7.0 hours. Nivolumab has a linear PK in the range of 0.3 to 10 mg/kg with dose- proportional increase in the maximum concentration observed and in the area under the curve from zero to infinity, with low to moderate inter-subject variability observed at each dose level, i.e., a coefficient of variation ranging from 7%-45%. The geometric mean clearance after a single IV dose of nivolumab ranges from 0.13-0.19 mL/h/kg, whereas the mean volume of distribution ranges from 83-113 mL/kg across doses. The mean terminal half-life of nivolumab is 17-25 days, which is in accordance with the half-life of endogenous IgG4 antibodies, indicating that they share a similar elimination mechanism. The elimination and distribution of nivolumab is dose-independent in the dose ranges studied.

3.2.3 Safety of Nivolumab in Renal Cell Carcinoma

Patients enrolled in clinical trial CA209001 (n=39) received a single dose of nivolumab with potential retreatment in 3 months. The most common adverse events (AEs) were fatigue (56%), nausea (44%), proteinuria (38%), constipation (33%), back pain (33%), dry mouth (28%), vomiting (28%), rash (26%) and dyspnea (26%). No correlation was identified between nivolumab dose levels (0.3, 1, 3, or 10 mg/kg IV) and AE incidence or severity. All patients had at least one AE, and 32 patients (82%) had Grade 3 or 4 AEs. The three treatment-related severe AEs reported were Grade 2 hypothyroidism, Grade 2 anemia, and Grade 3 colitis. No drug-related deaths were noted.

Nivolumab-related AEs of any grade occurred in 75.2% of patients in the CA209003 trial (n = 306).[36] The most common drug-related AEs occurring in ≥ 5% of patients included fatigue (28.1%), rash (14.7%), diarrhea (13.4%), pruritus (10.5%), nausea (9%), decreased appetite (9%), and fever (6%). The majority of AEs were low grade, and only 14% of patients developed Grade 3/4 drug-related AEs. The most common Grade 3/4 drug-related AEs occurring in ≥ 1% of patients were fatigue (2%), pneumonitis (1%), diarrhea (1%), and AST/ALT increase (0.3% each). Drug-related serious adverse events (SAEs) occurred in 17% of patients. Grade 3/4 drug-related SAEs occurring in ≥ 1% of patients were pneumonitis (1.3%), and diarrhea (1%). The spectrum, incidence, and severity of nivolumab-related AEs was overall similar across the different dose levels used. Less common drug-related AEs included vitiligo, hepatitis, hypophysitis, and thyroiditis.

Treatment interruption and administration of corticosteroids were used to manage hepatic or gastrointestinal AEs, which overall were fully reversible. Hormone replacement therapy was used to manage endocrine AEs. Several patients with these AEs successfully restarted nivolumab therapy. Drug-related pneumonitis was noted in 3% of patients. Grade ≥ 3 pneumonitis developed in only 3 patients (1%). There was no clear relationship between the occurrence of pneumonitis and dose level, dose number, or tumor type. Low-grade pneumonitis generally resolved with discontinuation of treatment and administration of corticosteroids. Infliximab and/or mycophenolate were used as additional immunosuppressants in 3 patients, but it remains uncertain whether this provided additional benefit. Three drug-related deaths (1%) due to pneumonitis were noted. In two of these cases, early and aggressive intervention such as systemic corticosteroid therapy was not initiated, and this likely significantly contributed to the fatal event, whereas in the

3rd patient, concomitant administration of other anti-cancer agents (erlotinib and vinorelbine) may have aggravated toxicity.

In the CheckMate 025 study using a nivolumab dose of 3 mg/kg every 2 weeks, treatment-related AEs of any grade occurred in 319/406 patients (79%) following a median treatment duration of 5.5 months (range, <0.1 to 29.6). The most frequent treatment-related AEs were fatigue (33%), nausea (14%), and pruritus (14%). Grade 3/4 treatment-related AEs occurred in 19% of patients, with the most common Grade 3/4 AE being fatigue (2%). Treatment-related AEs leading to nivolumab discontinuation were noted in 8% of patients. There were no nivolumab-related deaths.[24]

Based on simulations of population pharmacokinetics and dose/exposure-response analyses showing that 240 mg of flat dose nivolumab monotherapy every 2 weeks is similar (with less than 6% difference) to the previously approved dose of 3 mg/kg every 2 weeks, the FDA modified on September 13, 2016 the recommended dosage regimen for nivolumab when combined with ipilimumab for unresectable or metastatic melanoma, nivolumab is administered at a dose of 1 mg/kg followed by ipilimumab 3 mg/kg every 3 weeks for four doses, followed by nivolumab monotherapy 240 mg flat dose every 2 weeks until disease progression or intolerable toxicity.

3.3 Background on Ipilimumab (Yervoy)

Ipilimumab is a fully humanized anti-CTLA-4 IgG1 monoclonal antibody that has been approved for the therapy of metastatic melanoma and has demonstrated improved overall survival as monotherapy (compared with peptide vaccine gp100), in combination with dacarbazine (compared with dacarbazine alone), and in combination with nivolumab (compared with either therapy alone).[31,37,38]

The toxicity profile of ipilimumab includes side effects associated with an immune mechanism of action (immune-related adverse events, irAEs). At 3 mg/kg given every 3 weeks as monotherapy,[37] the following AEs were observed in at least 10% of subjects: fatigue (42%), diarrhea (33%), nausea (35%), decreased appetite (27%), vomiting (24%), pruritus (24%), constipation (21%), rash (19%), cough (16%), abdominal pain (15%), headache (15%) and pyrexia (12%). Additional irAEs occurring in < 10% of subjects but with clinical relevance include: colitis (8%); endocrinopathies (8%) including hypothyroidism, hypopituitarism, hypophysitis, adrenal insufficiency, increased serum thyrotropin and decreased corticotropin; hepatotoxicity (4%) including hepatitis and elevations in AST/ALT; and vitiligo (2%). Other reported irAEs include nephritis, pneumonitis, meningitis, pericarditis, uveitis, iritis and hemolytic anemia. Overall irAEs occur in approximately 60% of subjects, of which 13% are Grade 3-4, and 0.8% were Grade 5.[37,39] Seven deaths were associated with an irAE: GI perforation (4 patients), colitis (1), liver failure (1), and Guillain-Barré Syndrome (1). In addition, 1 subject in the ipilimumab plus gp100 group of MDX010-20 clinical trial had a Grade 4 skin irAE (Stevens Johnson syndrome/toxic epidermal necrolysis/Lyell's syndrome), but died due to a treatment-related acute respiratory distress syndrome. The irAEs are related to T-cell activation and can be serious or life threatening. Specific management guidelines were addressed by Weber et al.[40]

3.3.1 Ipilimumab in Renal Cell Carcinoma

Although ipilimumab has not been used before in patients with RMC, it has been studied as monotherapy for the treatment of metastatic renal cell carcinomas (RCCs) in the Phase 2 clinical trial MDX010-11.[41] Two sequential cohorts were studied, each with a loading dose of 3mg/kg followed by 3 doses of either 1 mg/kg (group 3-1; n=21) or 3 mg/kg (group 3-3; n=40). Subjects with stable disease or partial or complete response were allowed additional treatment. The major toxicities were colitis (all Grade 3 & 4; 14% in group 3-1, 33% in group3-3) and hypophysitis (1 grade 3/4, 1 grade 1/2 in group 3-3; none in group 3-1). Most reported AEs were Grade 1/2 (57% in group 3-1, 35% in group3-3) or Grade 3 (38% in group 3-1, 48 % in group 3-3). There were 6 subjects (15%) with Grade 4 AEs in group 3-3. The most common treatment related AEs in group 3-1 (total 81%) and group 3-3 (total 93%) were diarrhea (38% & 40% respectively) and fatigue (33% and 38% respectively). Most AEs were manageable with appropriate treatment, including high dose corticosteroids and hormone replacement.

In group 3-1 (n=21), one subject had a PR of 18 months duration.[42] In group 3-3 (n=40), there were 5 partial responses of 7, 8, 12, 17 and 21 months duration, with an overall response rate of 12.5 %. Tumor response had a highly significant association with irAEs in these patients (response rate = 30% with irAE, 0% without irAE; P=0.009). Though not conclusive, the study indicates that a subset of metastatic RCC patients may have prolonged response to ipilimumab treatment and toxicities are manageable.

.

3.3.2 Nivolumab in Combination with Ipilimumab in Malignancies Other than RMC

Because anti-PD1 and anti-CTLA-4 antibodies use distinct mechanisms for immune activation, a phase I study was conducted testing the combination of nivolumab with ipilimumab in patients with advanced melanoma.[43] In this trial, patients were treated with intravenous doses of nivolumab and ipilimumab every 3 weeks for 4 doses, followed by nivolumab alone every 3 weeks for 4 doses (concurrent regimen). The combined treatment was subsequently administered every 12 weeks for up to 8 doses. In a sequenced regimen, patients previously treated with ipilimumab received nivolumab every 2 weeks for up to 48 doses.

A total of 53 patients received concurrent therapy with nivolumab and ipilimumab, whereas 33 received sequenced treatment. The objective-response rate (according to modified WHO criteria) for all patients in the concurrent-regimen group was 40%. Evidence of clinical activity (conventional, unconfirmed, or immune-related response or stable disease for ≥24 weeks) was observed in 65% of patients. At the maximum doses that were associated with an acceptable level of AEs (nivolumab at a dose of 1 mg per kilogram of body weight and ipilimumab at a dose of 3 mg per kilogram), 53% of patients had an objective response, all with tumor reduction of ≥80% or more (**Figure 1**). Grade 3 or 4 adverse events related to therapy occurred in 53% of patients in the concurrent-regimen group but were qualitatively similar to previous experience with monotherapy and were generally reversible. Among patients in the sequenced-regimen group, 18% had grade 3 or 4 adverse events related to therapy and the objective-response rate was 20%.

The CheckMate 067 trial was a randomized, double-blind, phase III study of nivolumab plus ipilimumab versus either therapy alone.[31] A total of 945 previously untreated patients with

unresectable stage III or IV melanoma were randomized in a 1:1:1 ratio to each of the three treatment groups: nivolumab 3 mg/kg every 2 weeks vs ipilimumab 3 mg/kg every 3 weeks vs nivolumab 1 mg/kg every 3 weeks plus ipilimumab 3 mg/kg every 3 weeks x4 doses followed by nivolumab 3 mg/kg every 2 weeks. The median PFS was 11.5 months (95% confidence intervals [CI] 8.9-16.7) for the combination therapy, compared with 2.9 months (95% CI 2.8-3.4) for ipilimumab, and 6.9 months with nivolumab (95% CI 4.3-9.5). Grade 3 or 4 AEs were more frequent in the combination group (55% of patients), and least frequent in the nivolumab monotherapy group (16.3% of patients). No drug-related deaths were noted in the combination group, and AEs were all manageable with established guidelines.[31] The OS data have not yet been reported as the specified number of events remains to be reached. The combination of ipilimumab with nivolumab is also being studied in patients with advanced non–small cell lung cancer (NSCLC) in the ongoing phase III CheckMate 227 trial, following encouraging results from the phase I CheckMate 012 study which demonstrated a median PFS of 3.6 vs 8.0 months for nivolumab monotherapy vs combination therapy respectively.[44]

In metastatic renal cell carcinoma, the combination of nivolumab plus ipilimumab was first tested in the phase I CheckMate 016 study, which did not include patients with RMC. CheckMate 016 found that a dose schedule of nivolumab 1 mg/kg + ipilimumab 3 mg/kg produced considerably higher toxicity compared with nivolumab 3 mg/kg + ipilimumab 1 mg/kg (38.3% vs 61.7% grade 3-4 treatment-related AEs) without a significant benefit in efficacy (ORR 40.4% for both arms).[45] Subsequently, the randomized phase III CheckMate 214 clinical trial in patients with metastatic renal cell carcinoma (Clinicaltrials.gov NCT02231749) adopted the regimen of nivolumab 3 mg/kg + ipilimumab 1 mg/kg every 3 weeks x4 doses followed by maintenance nivolumab 3 mg/kg every 2 weeks.

Based upon these data, it will be very important to determine whether combination therapy with nivolumab and ipilimumab can offer a similar clinical benefit to patients with RMC.

Figure 1. Clinical Activity in Patients with Advanced Melanoma Who Received the Concurrent Regimen of Nivolumab and Ipilimumab.

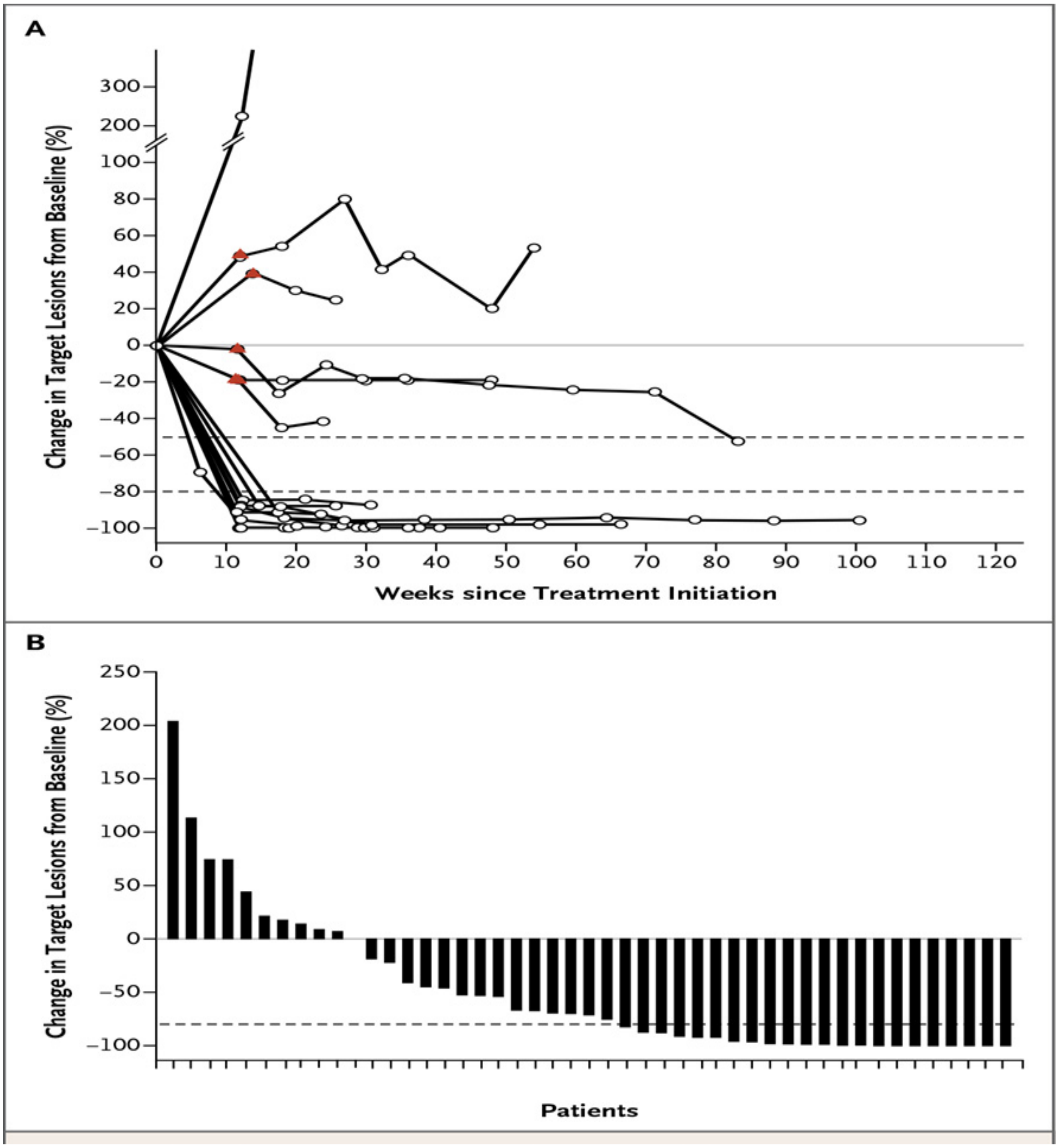

Panel A show changes from baseline in the tumor burden.
Panel B shows a representative waterfall plot of the maximum percentage change in target lesions, as compared with baseline measurements.

3.5 Rationale for Combining Nivolumab with Ipilimumab in RMC

RMC is a rare and highly aggressive tumor with close to universal fatality despite therapy.[46,47] Our group recently published the largest multi-institutional experience to date with this disease.[2] None of the patients in this study demonstrated an objective response to targeted therapies. Additionally, the median duration of targeted therapy was only 8 weeks. While 29% of the RMC patients had an objective response to cytotoxic chemotherapy, responses to cytotoxic chemotherapy were typically brief, with a median OS of 13 months. Furthermore, only 13% of patients survived longer than 2

years.[2] Novel therapeutic strategies are therefore needed for this deadly disease. Of note, RMC afflicts predominantly young patients with a median age of 28 years (range 9-48 years), who in the vast majority of cases are otherwise healthy and suffer from no additional co-morbidities other than sickle-cell trait.[2] Therefore, this patient population is more likely to tolerate the higher toxicity burden and derive more benefit from combination treatment regimens that can produce more potent and durable antitumor responses.

There are currently no *in vivo* immunocompetent animal models of RMC to study the efficacy of immune checkpoints therapies. To characterize the tumor immune microenvironment in RMC we evaluated Formalin-fixed paraffin-embedded (FFPE) specimens from 5 tumor tissues and 3 adjacent normal kidney samples. H-scores were generated by multiplying immunohistochemistry staining intensity scores by percentage positivity. RMC tissues showed significantly higher infiltration by CD3+ T lymphocytes (median H-score 31 vs 6, p=0.024), and CD4+FoxP3+ T regulatory cells (median H-score 5 vs 1, p=0.020) compared with normal kidney tissues. Tumor cell PD-L1 expression was found to be positive (>5%) in 3/5 cases (Figure 2), with one case showing strong PD-L1 expression in 78% of tumor cells. These results indicate that anti-PD-1 therapy may be a rational option for RMC therapy. Indeed, a recent case report demonstrated a gratifying clinical response in a patient with RMC treated with anti-PD-1 therapy (nivolumab).[48] Analysis of this patient's tumor tissue prior to initiating nivolumab treatment revealed a robust immune infiltrate with high percentage of CD4+ and CD8+ T lymphocytes as well as robust levels of PD-L1 and PD-1 expression.[48] The combination of anti-PD1 with anti-CTLA4 immune checkpoint therapy may lead to immunological changes that will augment this antitumor response, or produce clinical responses in patients without such pre-existing immune recognition. Recent data from our department has shown that loss of the IFN-γ signaling pathway on tumor cells can induce primary resistance to anti-CTLA-4 therapy.[49] To determine whether such defects are present in RMC tissues, we performed RNA sequencing of 11 RMC tissues derived from 9 patients, using uninvolved kidney tissue as control. Ingenuity pathway analysis (Ingenuity Systems Inc. Redwood CA) identified significant upregulation of genes associated with the IFN-γ pathway (overlap p-value < 0.001) indicating that RMC may be sensitive to anti-CTLA-4 therapy. **Therefore, we hypothesize that combined immune checkpoint therapy using nivolumab plus ipilimumab will produce a potent and durable antitumor response that will improve the ORR of patients with RMC.** To test this hypothesis, we propose to conduct a single-arm phase II trial combining ipilimumab and nivolumab in patients with RMC. We anticipate that this combination will significantly improve the ORR compared with our historical control of 29% achieved with cytotoxic chemotherapy, and will lead to durable remissions and improved survival, which currently is dismal with median OS of only 13 months as measured from initial diagnosis.

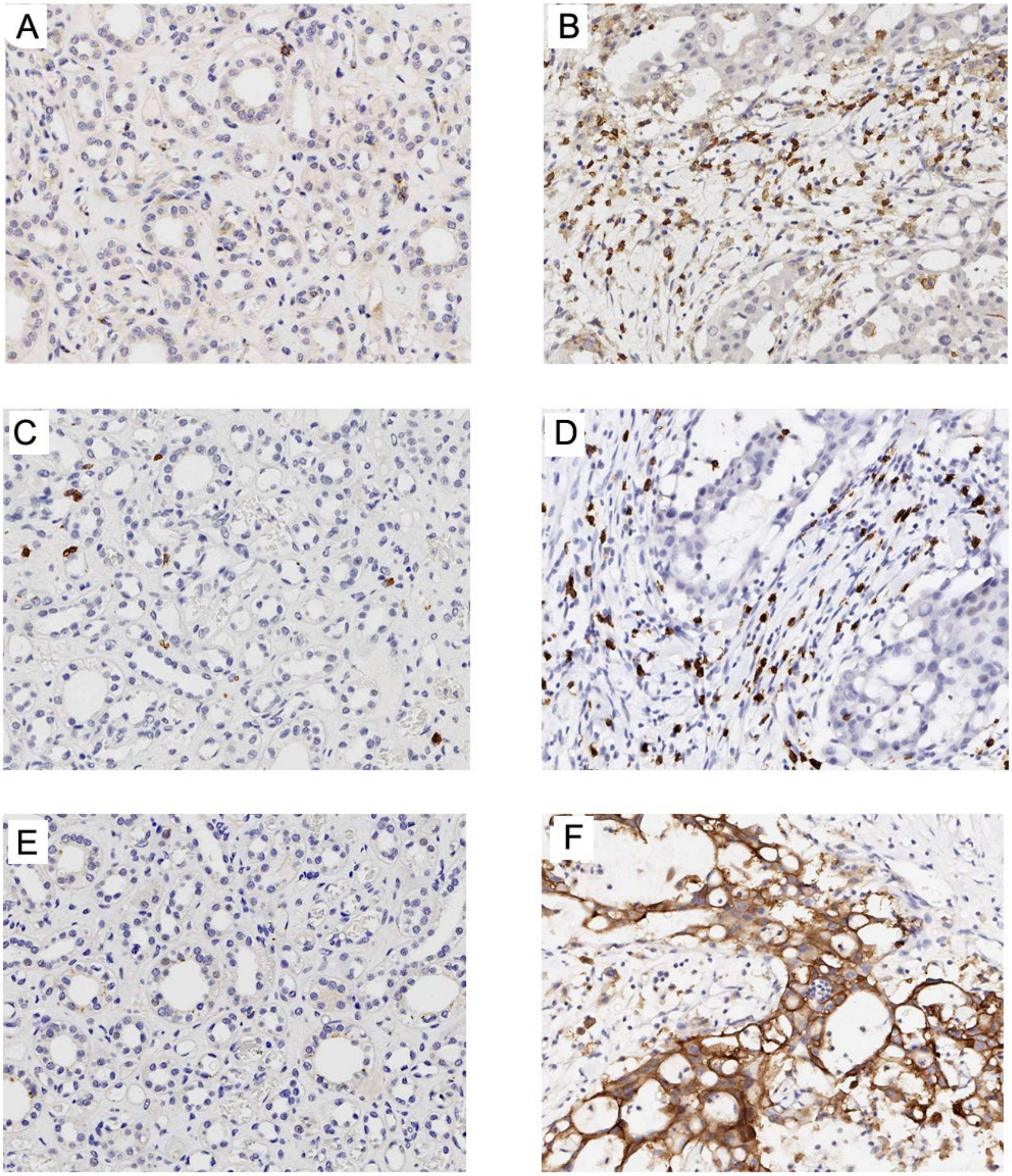

Figure 2. Representative immunohistochemistry staining for CD4 (A and B), CD8 (C and D), and PD-L1 (E and F) expression in RMC tissues (B, D, F) versus control uninvolved kidney (A, C, E).

3.5.1 Dose Justification

We will use the combination dosing scheme that was shown to have the best balance between efficacy and toxicity in patients with metastatic RCC in the phase I CheckMate 016 study and was subsequently adopted in the phase III CheckMate 214 trial in the same patient population (Clinicaltrials.gov NCT02231749): nivolumab will be administered at a dose of 3 mg/kg every 3 weeks in combination with ipilimumab 1 mg/kg every 3 weeks for up to 4 doses, followed by maintenance with single-agent nivolumab 480 mg flat dose every 4 weeks until disease progression or intolerable toxicity. The maintenance nivolumab flat dose of 480 mg every 4 weeks has been shown to produce similar exposure/response outcomes compared with every 2 week dosing schedules across multiple tumor types based on quantitative clinical pharmacology analyses and safety assessments,[50] and has thus been incorporated into clinical trials (Clinicaltrials.gov NCT02713867 and NCT02714218).

4.0 ELIGIBILITY CRITERIA

4.1 Signed Written Informed Consent:

1. Patients must give written informed consent prior to initiation of therapy, in keeping with the policies of the institution. Patients with a history of major psychiatric illness must be judged able to fully understand the investigational nature of the study and the risks associated with the therapy.

4.2 Inclusion Criteria:

1. Patients with locally advanced or metastatic RMC histologically confirmed by expert pathology review and loss of SMARCB1 staining by immunohistochemistry. Patients with advanced or metastatic unclassified renal cell carcinoma with medullary phenotype (a rare SMARCB1 negative RMC variant occurring in individuals without sickle hemoglobinopathies) are also eligible. The Principal Investigator (PI) is the final arbiter in questions related to eligibility.

2. Patients will be eligible regardless of whether they have had prior nephrectomy or still have their primary tumor *in-situ*.

3. Patients must have at least one measurable site of disease, defined as a lesion that can be accurately measured in at least one dimension (longest diameter to be recorded) and measures ≥ 15 mm with conventional techniques or ≥ 10 mm with more sensitive techniques such as MRI or spiral CT scan. If the patient has had previous radiation to the marker lesion(s), there must be evidence of progression since the radiation.

4. Patients should be willing to provide a newly obtained fresh core biopsy of a tumor lesion. Not required if there is a recently obtained fresh specimen on an IRB approved correlated trial up to 6 weeks (42 days) prior to initiation of treatment on Day 1.

5. Patients can be either naïve for any previous systemic treatment or have had any number of prior systemic therapies. However, patients must not have received prior anticancer therapy with anti-PD1, anti-PD-L1, or anti-CTLA-4 immune checkpoint inhibitors.

6. There must be evidence of progression on or after last treatment regimen received.

7. ECOG performance status 0-2
   - NOTE: If subject is unable to walk due to paralysis, but is mobile in a wheelchair, subject is considered to be ambulatory for the purpose of assessing their performance status.

8. Age (at the time of consent/assent): ≥ 18 years

9. Consent to MD Anderson companion laboratory protocol 2014-0938

10. Within 14 days of the first dose of the study drugs, patients must have adequate organ and marrow function prior to study entry as defined below:

• Hemoglobin[a]	≥9 g/dl (treatment allowed)
• Absolute neutrophil count[b]	≥1,000/µL
• Platelets	≥75,000/µL
• total bilirubin	≤ 1.5 mg/dl
• AST(SGOT) or ALT (SGPT)	≤ 2.5 X institutional ULN, except in known hepatic metastasis, wherein may be ≤ 5 x ULN
• Serum Creatinine[c]	≤ 1.5 x ULN by gender (as long as patient does not require dialysis)

[a] May receive transfusion
[b]Without growth factor support (filgrastim or pegfilgrastim) for at least 14 days
[c]If creatinine is not <1.5×ULN, then calculate by Cockcroft-Gault methods or local institutional standard and CrCl must be >30 mL/kg/1.73 m^2

11 INR and PTT ≤ 1.5 x ULN prior to study entry. Therapeutic anticoagulation with warfarin is allowed if target INR ≤ 3 on a stable dose of warfarin or on a stable dose of low molecular weight (LMW) heparin for > 2 weeks (14 days) at the time of enrollment.

12 Patients with controlled brain metastases are allowed on protocol if they had solitary brain metastases that was surgically resected or treated with radiosurgery or Gamma knife, without recurrence or edema for 1 month (4 weeks).

13 Women of childbearing potential (WOCBP) must have a negative serum or urine pregnancy test (minimum sensitivity 25 IU/L or equivalent units of HCG) within 24 hours prior to the start of the study drug.

14 Women must not be breastfeeding.

15 WOCBP must agree to follow instructions for method(s) of contraception from the time of enrollment for the duration of treatment with study drug (s) plus 5 half-lives of study drug (s) plus 30 days (duration of ovulatory cycle) for a total of 5 months post treatment completion.

16 Men who are sexually active with WOCBP must agree to follow instructions for method(s) of contraception for the duration of treatment with study drug (s) plus 5 half-lives of study drug (s) plus 90 days duration of sperm turnover) for a total of 5 months post-treatment completion.

17 Azoospermic males and WOCBP who are continuously not heterosexually active are exempt from contraceptive requirements. However WOCBP must still undergo pregnancy testing as described in these sections.

Investigators shall counsel WOCBP and male subjects who are sexually active with WOCBP on the importance of pregnancy prevention and the implications of an unexpected pregnancy Investigators shall advise WOCBP and male subjects who are sexually active with WOCBP on the use of highly effective methods of contraception. Highly effective methods of contraception have a failure rate of < 1% per year when used consistently and correctly.

At a minimum, subjects must agree to the use one highly effective method of contraception as listed below:

4.2.1 Highly Effective Methods of Contraception

- Hormonal methods of contraception including combined oral contraceptive pills, vaginal ring, injectables, implants, and intrauterine devices (IUDs) such as Mirena by WOCBP subject or male subject's WOCBP partner.
- Nonhormonal IUDs, such as ParaGard
- Tubal ligation
- Vasectomy
- Complete Abstinence*

*Complete abstinence is defined as complete avoidance of heterosexual intercourse and is an acceptable form of contraception for all study drugs. Abstinence is only acceptable when this is in line with the preferred and usual lifestyle of the subject. Periodic abstinence (eg, calendar, ovulation, symptothermal, profession of abstinence for entry into a clinical trial, post-ovulation methods) and withdrawal are not acceptable methods of contraception. Subjects who choose complete abstinence are not required to use a second method of contraception, but female subjects must continue to have pregnancy tests. Acceptable alternate methods of highly effective contraception must be discussed in the event that the subject chooses to forego complete abstinence.

4.3 Exclusion Criteria:

1. Patients must not have any other malignancies within the past 2 years except for *in situ* carcinoma of any site, or adequately treated (without recurrence post-resection or post-radiotherapy) carcinoma of the cervix or basal or squamous cell carcinomas of the skin.

2. Patients currently receiving anticancer therapies or who have received anticancer therapies (including chemotherapy and targeted therapy) within 2 weeks (14 days) prior to study Day are excluded. Patients who have completed palliative radiation therapy more than 14 days prior to the first dose of the combination ipilimumab plus nivolumab are eligible.

3. Patients, who have had a major surgery or significant traumatic injury (injury requiring > 4 weeks (28 days) to heal) within 4 weeks (28 days) of start of study drug, patients who have not recovered from the side effects of any major surgery (defined as requiring general anesthesia) or patients that are expected to require major surgery, other than cytoreductive nephrectomy ± retroperitoneal lymph node dissection, during the course of the study.

4. Patients who have organ allografts.

5. Known or suspected autoimmune disease. Patients with a history of inflammatory bowel disease (including Crohn's disease and ulcerative colitis) and autoimmune disorders such as rheumatoid arthritis, systemic progressive sclerosis [scleroderma], Systemic Lupus Erythematosus or autoimmune vasculitis [e.g., Wegener's Granulomatosis] are excluded from this study. Patients with a history of Hashimoto's thyroiditis only requiring hormone replacement, Type I diabetes, or psoriasis not requiring systemic treatment, or conditions not expected to recur in the absence of an external trigger are allowed to participate.

6. Known history of testing positive for human immunodeficiency virus (HIV) or known acquired immunodeficiency syndrome (AIDS).

7. Positive test for hepatitis B virus (HBV) using HBV surface antigen (HBVsAg) test or positive test for hepatitis C virus (HCV) using HCV ribonucleic acid (RNA) or HCV antibody test indicating acute or chronic infection. If hepatitis C antibody test is positive then active infection has to be confirmed by hepatitis C RNA testing for the patient to be excluded.

8. Any underlying medical condition, which in the opinion of the Investigator, will make the administration of study drug hazardous or obscure the interpretation of adverse events, such as a condition associated with frequent diarrhea, uncontrolled nausea or vomiting

9. Patients must not have received prior anticancer therapy with anti-PD1, anti-PD-L1, or anti-CTLA-4 immune checkpoint inhibitors.

10. Patients receiving any concomitant systemic therapy for renal cell cancer are excluded.

11. Patients must not be scheduled to receive another experimental drug while on this study.

12. Patients who are on high dose steroid (e.g., > 10mg prednisone daily or equivalent) or other more potent immune suppression medications (e.g., infliximab). Topical, inhaled, intra-articular, ocular, or intranasal corticosteroids (with minimal systemic absorption) are allowed. A brief course (<48 hours) of systemic corticosteroids for prophylaxis (eg, from contrast dye allergy) is permitted. Physiological corticosteroid replacement therapy for adrenal insufficiency is also permitted.

13. Patients who have any severe and/or uncontrolled medical conditions or other conditions that could affect their participation in the study such as:
    a. Symptomatic congestive heart failure of New York heart Association Class III or IV
    b. Unstable angina pectoris, symptomatic congestive heart failure, myocardial infarction within 6 months of start of study drug, serious uncontrolled cardiac arrhythmia or any other clinically significant cardiac disease
    c. Severely impaired lung function as defined as 02 saturation that is 92% or less at rest on room air
    d. Uncontrolled diabetes as defined by fasting serum glucose >1.5 x ULN
    e. Systemic fungal, bacterial, viral, or other infection that is not controlled (defined as exhibiting ongoing signs/symptoms related to the infection and without improvement) despite appropriate antibiotics or other treatment
    f. Known active or symptomatic viral hepatitis or chronic liver disease. Uncontrolled adrenal insufficiency

14. Patients must not have history of other diseases, metabolic dysfunction, physical examination finding, or clinical laboratory finding giving reasonable suspicion of a disease or condition that contraindicates the use of ipilimumab or nivolumab or that might affect the interpretation of the results of the study or render the subject at high risk from treatment complications.

15. Patients should not receive immunization with attenuated live vaccines within one week (7 days) of study entry or during study period.
    a. Note: Seasonal influenza vaccines for injection are generally inactivated flu vaccines and are allowed; however intranasal influenza vaccines (e.g., Flu-Mist®) are live attenuated vaccines, and are not allowed.

16. Uncontrolled brain or leptomeningeal metastases, including patients who continue to require glucocorticoids for brain or leptomeningeal metastases.

17. Female patients who are pregnant or breast feeding, or adults of reproductive potential who are not using effective birth control methods as defined above. If barrier contraceptives are being used, these must be continued throughout the trial by both sexes. Hormonal contraceptives are not acceptable as a sole method of contraception.

18. Any patients who cannot be compliant with the appointments required in this protocol must not be enrolled in this study.

5.0 TREATMENT PLAN

5.1 Patient Enrollment

All patients or legally acceptable representatives must personally sign and date, and receive a copy of the informed consent form (ICF) before any study specific screening procedures are performed. Standard medical practice procedures (CT, MRI, physical exam, blood tests) performed within the specified screening period may be used for screening.

Patients will be registered by the responsible study nurse or research coordinator. All patients will

be registered in the approved Office of Research Administration (ORA) database at MD Anderson.

All patients who sign an informed consent will be identified by a unique patient number. This number will be used to identify the patient throughout the clinical study and must be used on all study documentation related to that patient. The patient identification number must remain constant throughout the entire clinical study. A patient is considered enrolled when study medication is administered on Day 1.

5.2 Data Collection

Data will be entered into MD Anderson institutionally approved and compliant database(s). The database(s) have secure portal that requires users to login with validated credentials, uses approved encryption protocols as defined by institutional information security standards. Systems have granular data access controls to ensure that the minimal amount of information required to complete a task is presented, can handle de-linking and de-identification of patient information to maintain patient confidentiality if necessary. The system(s) are 21 CFR 11 compliant. Standard data collection, storage procedures, and quality assurance procedures will be followed, to ensure integrity and auditability of all information entered.

All patients will be registered in the University of Texas M. D. Anderson Cancer Center Office of Research Administration database. Registration will occur following informed consent process and prior to initiation of investigational intervention(s). All eligibility criteria must be satisfied.

5.3 Study Design and Duration

This is a single-arm phase II trial to determine the efficacy of combined immune checkpoint therapy using nivolumab plus ipilimumab in patients with RMC. All patients will be required to provide a pre-treatment core biopsy with documented tumor involvement by pathologist review up to 6 weeks (42 days) prior to initiation of treatment on Day 1. Patients will not be required to undergo a second biopsy procedure prior to starting study therapy (screening) if fresh frozen tissue in liquid nitrogen is available on an IRB approved correlated trial up to 6 weeks (42 days) prior to initiation of treatment on Day 1. These will be reviewed by the pathologist for confirmation of cancer. Eligible patients with locally advanced or metastatic RMC will receive nivolumab 3 mg/kg IV + ipilimumab 1 mg/kg IV every 3 weeks for up to 4 cycles (minimum of 2 cycles), if they do not develop toxicity that necessitates discontinuation of therapy. Patients who already had cytoreductive nephrectomy will receive up to 4 cycles of the combination therapy (minimum of 2 cycles), followed by a core biopsy of the most accessible tumor site determined at the physician's discretion. Patients will then proceed to receive maintenance nivolumab at a dose of 480 mg IV every 4 weeks for up to 2 years, or until disease progression or unacceptable treatment-related toxicity. Patients who have not had cytoreductive nephrectomy will be treated with the combination therapy of nivolumab 3 mg/kg IV + ipilimumab 1mg/kg IV every 3 weeks for up to 4 cycles (minimum of 2 cycles) with evaluation by a surgeon to determine if they are good candidates for cytoreductive nephrectomy by the end of the cycle #4. Patients who are deemed to be appropriate candidates for cytoreductive nephrectomy will undergo this surgery, and then, at 4-6 weeks postoperatively, will start maintenance nivolumab 480 mg IV every 4 weeks for up to 2 years, or until disease progression or unacceptable treatment-related toxicity. Patients who are not

good candidates for cytoreductive nephrectomy, but still demonstrate persistent disease response to the therapy, will undergo a core biopsy of the most accessible tumor site determined at the physician's discretion, and then proceed to receive maintenance nivolumab at a dose of 480 mg IV every 4 weeks for up to 2 years, or until disease progression or unacceptable treatment-related toxicity.

Patients will proceed to the maintenance phase of nivolumab 480 mg IV q4 weeks, if they have received at least 2 cycles of the combination, and as long as they remain progression-free after the induction phase. Maintenance nivolumab will not be started in patients with grade 4 adverse events (AEs) necessitating discontinuation of nivolumab and ipilimumab (as defined in Section 8.1). Patients with grade 3 AEs necessitating discontinuation of nivolumab and ipilimumab (as defined in Section 8.1) will only start maintenance nivolumab 480 mg IV q4 weeks if there is resolution of AEs to Grade 1 or baseline

Adverse events will be recorded by Common Terminology Criteria for Adverse Events (CTCAE) v4.03 with information regarding relationship to the study drug. Treatment response will be assessed using the Response Evaluation Criteria in Solid Tumors (RECIST) 1.1 criteria. Patients who are alive and free of progression at the time of analysis will be censored on the date they were last assessed for disease status (last follow-up) before starting a subsequent therapy. A core biopsy of the most accessible tumor site, determined at the physician’s discretion, will be performed upon disease progression during the combination of nivolumab/ipilimumab or the maintenance nivolumab phase. Patients will be allowed to continue study therapy after initial investigator-assessed RECIST 1.1-defined progression if they are deemed by the investigator to be deriving clinical benefit and tolerating study drug(s), as described in Section 8.2.

Patients will receive baseline staging studies with CT or MRI and be assessed for treatment response (RECIST 1.1) by CT or MRI at weeks 6 (+/- 7 days), week 12 (+/- 7 days) and every 8 weeks thereafter, as detailed in Sections 9 and 10 of the protocol. Patients who undergo cytoreductive nephrectomy at any point will have post-surgical, baseline staging studies with CT or MRI. Subsequently, restaging will be carried out every 8 weeks unless, in the investigators’ opinion, follow-up intervals need to be shorter.

Figure 3. Trial Design Schema

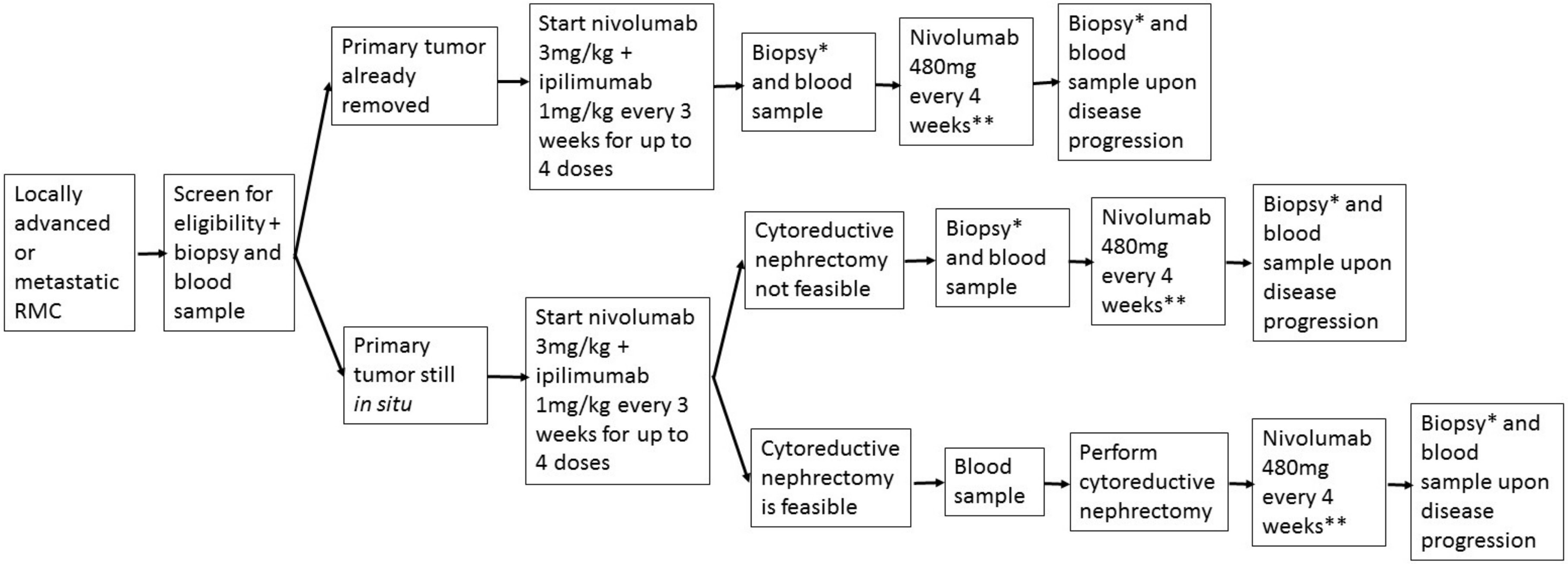

*All biopsies will be collected and analyzed under MD Anderson companion laboratory trial 2014-0938
**Continue maintenance nivolumab 480 mg IV every 4 weeks for up to 2 years, or if disease progression or unacceptable treatment-related toxicity. In patients who undergo cytoreductive nephrectomy, start maintenance nivolumab 4-6 weeks after surgery.

5.4 Study outcomes

The primary outcome is ORR, defined as the proportion of patients with a best response of CR or PR by RECIST 1.1 criteria recorded between Day 1 of the study and the date of objectively documented progression per RECIST 1.1 or the date of subsequent anti-cancer therapy, whichever occurs first. The goal of the trial is to significantly improve the ORR compared with the historical ORR of 29% achieved using conventional cytotoxic chemotherapies.

Secondary outcomes include:

- PFS, defined as the time from enrollment to progression (per RECIST 1.1criteria), subsequent anti-cancer therapy, or death due to any cause, whichever comes first. Patients who are free of these events will be censored on their last date of assessment.
- OS, defined as the time from the start of the study therapy to death due to any cause. Patients who are alive will be censored on their last date of contact.
- Time to an objective response, defined as the time from the start of the study therapy to initial documentation of objective response (PR or CR) by RECIST 1.1 criteria. Patients without response will be censored on their date of last response assessment.
- Duration of response, defined as the time from documentation of tumor response to the first observation of progressive disease (with subsequent confirmation), subsequent anti-cancer therapy, or to death due to any cause. Patients still in response at the time of analysis will be censored on their date of last response assessment.
- 12-week disease control rate, defined as the proportion of patients who are alive and demonstrate a CR, PR, or stable disease (SD) at Week 12 (Day 84) of the study.
- Toxicity as defined by CTCAE version 4.03 criteria.
- Safety will be assessed by quantifying the toxicities and grades experienced by subjects who received the trial therapy, including serious adverse events (SAEs). The attribution to drug, time-of-onset, duration of the event, its resolution, and any concomitant medications administered will be recorded. AEs will be analyzed including but not limited to all AEs, SAEs, fatal AEs, and laboratory changes.
- Assess modulation of CD4 and CD8 counts from the treatment in the tumor tissue and serum samples.

6.0 STUDY MEDICATIONS

6.1 Nivolumab

Nivolumab is a fully human, IgG4 (kappa) isotype, monoclonal antibody that binds PD-1. Nivolumab will be supplied in vials of 100 or 40 mg (10 mg/mL each) and packaged in an open-label fashion. Ten nivolumab vials (each 10 mL) will be packaged within a carton. The vials are not subject specific although there will be specific vial assignments by subject distributed by the Pharmacy in order to track drug usage and re-supply.

6.1.1 Dose Calculation of nivolumab

Total dose of nivolumab during the combination phase with ipilimumab should be calculated as in the following example (with possible 10% change):

Subject's actual body weight in kg x 3 mg = total dose in mg
Therefore, a subject weighing 70 kg who is to receive a dose of 3 mg/kg would be administered 70 mg of nivolumab (70 kg x 3 mg/kg = 210 mg). Dose adjustment is not allowed.

Maintenance nivolumab (started after completion of combination with ipilimumab) will be administered at a flat dose of 480 mg every 4 weeks. Dose adjustment is not allowed except for patients who are underweight. This is defined as weight loss more than 10% of 80 kg i.e. 72 kg or less. In these cases we will administer a 6 mg/kg dosing in the maintenance phase.

6.1.2 Preparation and Dispensing of Nivolumab

The product storage manager should ensure that the study drug is stored in accordance with the environmental conditions (temperature, light, and humidity) as determined by the Investigator Brochure. If concerns regarding the quality or appearance of the study drug arise, do not dispense the study drug and contact BMS immediately.

Investigational product documentation must be maintained that includes all processes required to ensure drug is accurately administered. This includes documentation of drug storage, administration and, as applicable, storage temperatures, reconstitution, and use of required processes (e.g. required diluents, administration sets).

Nivolumab vials must be stored at a temperature of 2°C to 8°C and should be protected from light. If stored in a glass front refrigerator, vials should be stored in the carton. Recommended safety measures for preparation and handling of nivolumab include laboratory coats and gloves.

For details on prepared drug storage and use time of nivolumab under room temperature/light and refrigeration, please refer to the Investigator Brochure section for "Recommended Storage and Use Conditions". Care must be taken to assure sterility of the prepared solution as the product does not contain any anti-microbial preservative or bacteriostatic agent. No incompatibilities between nivolumab and polyolefin bags have been observed.

Nivolumab is to be administered as a 60 +/- 15 minute IV infusion, using a volumetric pump with a 0.2/0.22 micron in-line filter at the protocol-specified dose. The drug can be diluted with 0.9% normal saline for delivery but the total drug concentration of the solution cannot be below 0.35 mg/ml. It is not to be administered as an IV push or bolus injection. At the end of the infusion, flush the line with a sufficient quantity of normal saline.

6.1.3 Administration of Nivolumab

Patients will receive nivolumab 3 mg/kg as a 60 +/- 15 minute IV infusion starting on Day 1 of the trial and every 3 weeks x4 doses, followed by maintenance nivolumab 480 mg flat dose as a 60 +/- 15 minute IV infusion every 4 weeks until disease progression or intolerability. Dosing calculations during combination with ipilimumab should be based on the body weight assessed at the start of each cycle as described above. All doses should be rounded as needed to reduce vial wastage. The screening body weight may be used for dosing of cycle 1. There will be no nivolumab dose

escalations or reductions allowed. Patients may be dosed no less than 19 days from previous dose during the combination phase with ipilimumab. During the maintenance phase, patients may be dosed with nivolumab no less than 12 days from the previous dose. If a subject cannot receive a following dose of the cycle within the designated time frame, it will be omitted and the next dose received will be considered Day 1 of the next cycle.

Nivolumab is administered as an IV infusion only.

Calculate **Total Nivolumab Dose** during the combination phase with ipilimumab as follows:
Patient body weight in kg x 3 mg= total dose in mg

Calculate **Total Infusion Volume** as follows:
(Total nivolumab dose in mg ÷ 10 mg/mL) + dilution volume = total infusion volume in mL

Calculate **Total Drug Concentration** as follows:
Total nivolumab dose in mg ÷ Total infusion volume nivolumab dose in ml = total infusion volume in mg/mL.
Total concentration cannot be below 0.35 mg/mL.

Calculate **Rate of Infusion** as follows:
Total infusion volume in mL ÷ 60 minutes = rate of infusion in mL/min.

Example:
A patient weighing 70 kg (157 lb) would be administered a total dose of **210** mg of nivolumab (70 kg x 3 mg/kg = 210 mg).
The total infusion volume would be **21** mL (210 mg ÷ 10 mg/mL = 21 mL) if no dilution volume is added.
The total drug concentration would be **10** mg/mL (210 mg ÷ 21 mL = 10 mg/mL).
The rate of infusion would be **0.70** mL/min in 60 minutes (21 mL ÷ 60 minutes = 0.70 mL/min).

6.1.4 Patient Monitoring During Infusion

Patients will be monitoring by standard of care vital signs during nivolumab infusion.

6.1.5 Treatment of nivolumab Related Infusion Reactions

Since nivolumab contains only human immunoglobulin protein sequences, it is unlikely to be immunogenic and induce infusion or hypersensitivity reactions. However, if such a reaction were to occur, it might manifest with fever, chills, rigors, headache, rash, pruritus, arthralgias, hypo- or hypertension, bronchospasm, or other symptoms. All Grade 3 or 4 infusion reactions should be reported within 24 hours to BMS and reported as an SAE if criteria are met. Infusion reactions should be graded according to NCI CTCAE (version 4.03) guidelines.

Treatment recommendations for nivolumab related infusion reactions are provided below and may be modified based on MD Anderson treatment standards and guidelines, as appropriate:

For Grade 1 symptoms (Mild reaction; infusion interruption not indicated; intervention not indicated):
Remain at bedside and monitor subject until recovery from symptoms. The following prophylactic premedications are recommended for future infusions: diphenhydramine 50 mg (or equivalent) and/or acetaminophen 325 to 1000 mg at least 30 minutes before additional nivolumab administrations.

For Grade 2 symptoms (Moderate reaction requires therapy or infusion interruption but responds promptly to symptomatic treatment [e.g. antihistamines, non-steroidal anti-inflammatory drugs, narcotics, corticosteroids, bronchodilators, IV fluids]; prophylactic medications indicated for ≤ 24 hours):
Stop the nivolumab infusion, begin an IV infusion of normal saline, and treat the subject with diphenhydramine 50 mg IV (or equivalent) and/or acetaminophen 325 to 1000 mg; remain at bedside and monitor subject until resolution of symptoms. Corticosteroid or bronchodilator therapy may also be administered as appropriate. If the infusion is interrupted, then restart the infusion at 50% of the original infusion rate when symptoms resolve; if no further complications ensue after 30 minutes, the rate may be increased to 100% of the original infusion rate. Monitor subject closely. If symptoms recur then no further nivolumab will be administered at that visit. Administer diphenhydramine 50 mg IV, and remain at bedside and monitor the subject until resolution of symptoms. The amount of study drug infused must be recorded on the case report form (CRF). The following prophylactic premedications are recommended for future infusions: diphenhydramine 50 mg (or equivalent) and/or acetaminophen 325 to 1000 mg should be administered at least 30 minutes before additional nivolumab administrations. If necessary, corticosteroids (recommended dose: up to 25 mg of IV hydrocortisone or equivalent) may be used.

For Grade 3 or Grade 4 symptoms [Severe reaction, Grade 3: prolonged (i.e. not rapidly responsive to symptomatic medication and/or brief interruption of infusion); recurrence of symptoms following initial improvement; hospitalization indicated for other clinical sequelae (e.g. renal impairment, pulmonary infiltrates), Grade 4: life-threatening; pressor or ventilatory support indicated]:
Immediately discontinue infusion of nivolumab. Begin an IV infusion of normal saline, and treat the subject as follows: Recommend bronchodilators, epinephrine 0.2 to 1 mg of a 1:1,000 solution for subcutaneous administration or 0.1 to 0.25 mg of a 1:10,000 solution injected slowly for IV administration, and/or diphenhydramine 50 mg IV with methylprednisolone 100 mg IV (or equivalent), as needed. Subject should be monitored until the investigator is comfortable that the symptoms will not recur. Nivolumab will be permanently discontinued. Institutional guidelines will be followed for the treatment of anaphylaxis. Remain at bedside and monitor subject until recovery from symptoms. In the case of late-occurring hypersensitivity symptoms (e.g. appearance of a localized or generalized pruritus within 1 week after treatment), symptomatic treatment may be given (e.g. oral antihistamine or corticosteroids).

6.2 Ipilimumab

Ipilimumab is a human immunoglobulin G (IgG1) κ anti-CTLA-4 monoclonal antibody (mAb). It is supplied in clear, colorless solution at a concentration of 5 mg/ml in a 40ml vial.

Ipilimumab (BMS-734016) Injection (5 mg/ml), must be stored refrigerated (2 - 8°C) with protection from light. In preparation of infusion, ipilimumab may be stored in IV infusion bags (PVC, non-PVC/non-DEHP) or glass infusion containers at room temperature or refrigerated (2°C - 8°C) for up

to 24 hours. Drug must be completely delivered to the subject within 24 hours of preparation. This includes any time in transit plus the total time for the infusion.

6.2.1 Dose Calculation of Ipilimumab

Total dose should be calculated as in the following example:
Subject's actual body weight in kg x 1 mg = total dose in mg

Therefore, a subject weighing 70 kg who is to receive a dose of 1 mg/kg would be administered 70 mg of ipilimumab (70 kg x 1 mg/kg = 70 mg). Dose adjustment is not allowed.

6.2.2 Administration of Ipilimumab

Each patient will receive ipilimumab at 1 mg/kg dose IV for up to 4 doses. Ipilimumab will be administered as a 90 +/- 15 minute IV infusion,[51] using a volumetric pump with a 0.2 to 1.2 micron in-line filter at the protocol-specified dose. The drug can be diluted with 0.9% normal saline or 5% Dextrose Injection to concentrations between 1 mg/mL and 4 mg/mL. It is not to be administered as an IV push or bolus injection. Care must be taken to assure sterility of the prepared solutions, since the drug product does not contain any antimicrobial preservatives or bacteriostatic agents.

Please refer to the ipilimumab Investigator Brochure for further details regarding preparation/administration.

6.2.3 Patient monitoring during Ipilimumab infusion

Patients will be monitoring by standard of care vital signs during nivolumab infusion.

6.2.4 Treatment of Ipilimumab infusion Reactions

Since ipilimumab contains only human protein sequences, it is less likely that any allergic reaction will be seen in patients. However, it is possible that infusion of ipilimumab will induce a cytokine release syndrome that could be evidenced by fever, chills, rigors, rash, pruritus, hypo- or hypertension, bronchospasm or other symptoms. No prophylactic pre-medication will be given unless indicated by previous experience in an individual patient.

Reactions should be treated based upon the following recommendations:
For mild symptoms (e.g., localized cutaneous reactions such as mild pruritus, flushing, rash):

- Decrease the rate of infusion until recovery from symptoms, remain at bedside and monitor patient.
- Complete the ipilimumab infusion at the initial planned rate.
- Diphenhydramine 50 mg IV will be given prior to subsequent doses for patient who experience infusion reactions of ≥ grade 1.
- Premedication with diphenhydramine may otherwise be given at the discretion of the Investigator for subsequent doses of ipilimumab.

For moderate symptoms (any symptom not listed above [mild symptoms] or below [severe symptoms] such as generalized pruritus, flushing, rash, dyspnea, hypotension with systolic BP >80 mmHg):

- Interrupt ipilimumab;

- Administer diphenhydramine 50 mg IV;
- Monitor patient closely until resolution of symptoms;
- Corticosteroids may abrogate any beneficial immunologic effect, but may be administered at the discretion of the treating physician;
- Resume ipilimumab infusion after recovery of symptoms;
- At the discretion of the treating physician, ipilimumab infusion may be resumed at *one half the initial infusion rate, then increased incrementally to the initial infusion rate.*
- If symptoms develop after resumption of the infusion, the infusion should be discontinued and no additional ipilimumab should be administered that day;
- The next dose of ipilimumab will be administered at its next scheduled time and may be given with pre-medication (diphenhydramine and acetaminophen) and careful monitoring, following the same treatment guidelines outlined above;
- At the discretion of the treating physician additional oral or IV antihistamine may be administered prior to dosing with ipilimumab.

For severe symptoms (e.g., any reaction such as bronchospasm, generalized urticaria, systolic blood pressure <80 mm Hg, or angioedema):

- Immediately discontinue infusion of ipilimumab, and disconnect infusion tubing from the subject;
- Consider bronchodilators, epinephrine 1 mg IV or subcutaneously, and/or diphenhydramine 50 mg IV, with Solu-Medrol 100 mg IV, as needed.
- Patients should be monitored until the Investigator is comfortable that the symptoms will not recur;
- No further ipilimumab will be administered;

In case of late-occurring hypersensitivity symptoms (e.g., appearance within one week after treatment of a localized or generalized pruritus), symptomatic treatment may be given (e.g., oral antihistamine, or corticosteroids).

6.3 Concomitant Therapies

The following considerations apply during the entire duration of the study:

- No other approved or investigational anticancer treatment will be permitted during the study period, including chemotherapy, biologic agents, hormone therapy or immunotherapy except for bisphosphonates. No other investigational drug may be used during treatment on this protocol, and concurrent participation in another therapeutic clinical trial is not allowed.
- No anticancer agents other than the study medications should be given to patients. If such agents are required for a patient then the patient must first be withdrawn from the study.
- Surgery or radiation therapy is allowed for palliation purposes or management of intercurrent illness which, in the judgment of the treating physician, is not related to disease progression. After the patient has received at least 2 infusions of the combination of nivolumab and ipilimumab then further therapy on protocol can be held for up to 4 weeks (28 days) to allow for recovery from surgical or other interventions.
- Growth factors (e.g.G-CSF, GM-CSF, erythropoietin, platelets growth factors etc.) are not to be administered prophylactically but may be prescribed by the investigator for rescue from severe hematologic events, if this is thought to be appropriate.

- No chronic treatment with systemic steroids or other immunosuppressive agents, with the exception of physiological corticosteroid replacement therapy for adrenal insufficiency. Topical or inhaled corticosteroids are allowed.
- Live vaccines should not be administered while a patient is on protocol.
- Patients who develop venous thromboembolism (pulmonary embolisms or deep venous thrombosis) may be treated as indicated by LMW heparin and stay on protocol at the discretion of the treating physician.
- The patient will provide a list of medications, including over the counter agents, taken prior to enrollment. The list will be update during clinic visits while on treatment. Concurrent medications related to co-morbidity (e.g. hypertension, diabetes, etc.) will be recorded in the miedcal record . The name, dose, date start and stop (as accurately as possible) along with indication of the medication will be collected.

6.4 Dose Modifications and Interruptions

Based upon toxicity profiles on past clinical trials, [31,43,44] the doses of nivolumab and ipilimumab proposed in this protocol are within the maximum tolerated doses. Therefore, dose reductions or modifications of nivolumab, ipilimumab will not be allowed.

Dosing interruptions > 6 weeks that occur for non-drug-related reasons may be allowed if approved by the Principal Investigator. Prior to re-initiating treatment in a subject with a dosing interruption lasting > 6 weeks, the UTMDACC IND Office must be notified. Tumor assessments should continue as per protocol even if dosing is interrupted.

As described above, after the patient has received at least 2 infusions of the combination of nivolumab with ipilimumab then further combination treatment can be held for up to 4 weeks (28 days) to allow for recovery from surgery, radiation therapy, or other interventions for palliation purposes or management of intercurrent illness which, in the judgment of the treating physician, is not related to disease progression.

Immune-related Adverse Events (irAEs) are defined as those occurring during immune checkpoint therapy, often necessitate immunosuppression and have no other alternate etiology. The nivolumab (http://packageinserts.bms.com/pi/pi_opdivo.pdf) and ipilimumab (http://packageinserts.bms.com/pi/pi_yervoy.pdf) package inserts provide detailed evaluation and management guidelines for the following types of ir-AEs: pneumonitis, colitis, hepatitis, endocrinopathies, nephritis/renal dysfunction, rash, and encephalitis. As a general principle, nivolumab and ipilimumab should be withheld or permanently discontinued in patients with moderate or severe irAEs. Depending on the nature of the irAE, it should be managed with corticosteroids and/or hormone-replacement therapy. The corticosteroids should be tapered down upon improvement of the irAE to Grade ≤1. Depending on the severity of the irAE, restarting of immune checkpoint therapy may be considered. Prior to re-initiating treatment in a subject with a dosing interruption lasting > 6 weeks, the Principal Investigator must be consulted. Tumor assessments should continue as per protocol even if dosing is interrupted. Patients experiencing any Grade 4 non-hematological toxicity should be taken off study.

Discontinuation criteria for nivolumab and nivolumab are described in section 8.1.

6.5 Nivolumab and Ipilimumab Combination When both nivolumab and ipilimumab are to be administered on the same day, separate infusion bags and filters must be used for each infusion.

Nivolumab is to be administered first. The nivolumab infusion must be promptly followed by a saline flush to clear the line of nivolumab before starting the ipilimumab infusion.

7.0 CORRELATIVE STUDIES/TUMOR TISSUE COLLECTION

All samples will be collected and analyzed per a separate IRB-approved protocol (Refer to appendix 1).

8.0 DISCONTINUATION OF THERAPY

Patients MUST be discontinued from study therapy AND withdrawn from the study for the following reasons:

- Withdrawal of informed consent (subject's decision to withdraw for any reason)
- Any clinical adverse event, laboratory abnormality or intercurrent illness which, in the opinion of the Investigator, indicates that continued treatment with study therapies is not in the best interest of the subject
- Termination of the study by MD Anderson Cancer Center
- Imprisonment or the compulsory detention for treatment of either a psychiatric or physical (e.g., infectious disease) illness.

Patients whose treatment is interrupted or permanently discontinued due to an AE or abnormal laboratory value suspected to be related to study drug must be followed at least weekly until the adverse event or abnormal laboratory resolves or returns to grade 1. This may be done by telephone correspondence. If a patient requires, due to treatment-related toxicity, a dose delay of > 28 days from the intended day of the next scheduled dose, then the patient must be discontinued from the study.

8.1 Discontinuation Criteria for Nivolumab and Ipilimumab

Nivolumab and ipilimumab administration should be discontinued for the following:

- Any Grade ≥ 2 drug-related uveitis, eye pain, or blurred vision that does not respond to topical therapy and does not improve to Grade 1 severity within the re-treatment period OR requires systemic treatment
- Any Grade 3 non-skin, drug-related adverse event lasting > 7 days, with the following exceptions for laboratory abnormalities, drug-related bronchospasm, hypersensitivity reactions, and infusion reactions:
  - Grade 3 drug-related laboratory abnormalities do not require treatment discontinuation except:
    - Grade 3 drug-related thrombocytopenia > 7 days or associated with bleeding requires discontinuation.
    - Any drug-related liver function test (LFT) abnormality that meets the following criteria require discontinuation:
      - Aspartate aminotransferase (AST) or alanine aminotransferase (ALT) > 5-10x upper limit of normal (ULN) for > 2 weeks
      - AST or ALT > 10x ULN
      - Total bilirubin > 5x ULN

  - Concurrent AST or ALT > 3x ULN and total bilirubin > 2x ULN
- Grade 3 drug-related bronchospasm, hypersensitivity reaction, or infusion reaction of any duration requires discontinuation

- Any Grade 4 drug-related adverse event or laboratory abnormality, except for the following events which do not require discontinuation:
  - Grade 4 neutropenia ≤ 7 days
  - Grade 4 lymphopenia or leukopenia
  - Isolated Grade 4 electrolyte imbalances/abnormalities that are not associated with clinical sequelae and are corrected with supplementation/appropriate management within 72 hours of their onset.
  - Grade 4 amylase or lipase abnormalities that are not associated with symptoms or clinical manifestations of pancreatitis. It is recommended to consult with the PI for Grade 4 amylase or lipase abnormalities.
- Any dosing interruption lasting > 6 weeks with the following exceptions:
  - Dosing interruptions to allow for prolonged steroid tapers to manage drug-related adverse events are allowed. Prior to re-initiating treatment in a subject with a dosing interruption lasting > 6 weeks, the Principal Investigator must be consulted. Tumor assessments should continue as per protocol even if dosing is interrupted.
  - Dosing interruptions > 6 weeks that occur for non-drug-related reasons may be allowed if approved by the Principal Investigator. Prior to re-initiating treatment in a subject with a dosing interruption lasting > 6 weeks, the UTMDACC IND Office must be notified. Tumor assessments should continue as per protocol even if dosing is interrupted.
- Any adverse event, laboratory abnormality, or intercurrent illness which, in the judgment of the Investigator, presents a substantial clinical risk to the subject with continued nivolumab dosing.
- Any Grade 3 drug-related laboratory abnormality, with the following exceptions for asymptomatic amylase or lipase, AST, ALT, or total bilirubin: Grade 3 amylase or lipase abnormalities that are not associated with symptoms or clinical manifestations of pancreatitis do not require a dose delay. It is recommended to consult with the PI for Grade 3 amylase or lipase abnormalities.
- Any Grade 3 colitis, neurologic toxicity, symptomatic pancreatitis, or pneumonitis.
- Any Grade ≥3 Stevens-Johnson Syndrome of Toxic Epidermal Necrolysis (TEN).
- Persistent Grade 2 or 3 adverse reactions lasing 12 weeks or longer
- Immune-mediated adverse reactions requiring 10mg per day or greater prednisone or equivalent for more than 12 weeks

8.2 Continued Treatment Beyond Progression of Disease

Accumulating evidence indicates a minority of patients treated with immunotherapy may derive clinical benefit from continued treatment despite initial evidence of progressive disease.[52] For this reason, patients will be permitted to continue study therapy beyond initial investigator-assessed RECIST-defined progression as long as they meet the 2 criteria listed below.

- Investigator-assessed clinical benefit, and
- Subject is tolerating study drug

The assessment of clinical benefit should take into account whether the subject is clinically deteriorating and unlikely to receive further benefit from continued treatment. In addition, patients without disease progression per the immune-related Response Evaluation Criteria In Solid Tumors (irRECIST)[53,54] , defined in Section 11.5, will be permitted to continue study therapy.

All decisions to continue treatment beyond initial progression post-surgery must be discussed with the MDACC IND Office and documented in the study records.

Patients should discontinue study therapy upon evidence of further progression, defined as an additional 10% or greater increase in tumor burden from time of initial progression (including all target lesions and new measurable lesions) within 6 weeks from documentation of initial progression.

New lesions are considered measurable at the time of initial progression if the longest diameter is at least 10 mm (except for pathological lymph nodes, which must have a short axis of at least 15 mm). Any new lesion considered non-measurable at the time of initial progression may become measurable and therefore included in the tumor burden measurement if the longest diameter increases to at least 10 mm (except for pathological lymph nodes, which must have an increase in short axis to at least 15 mm).

For statistical analyses that include the investigator-assessed progression date, patients who continue treatment beyond initial investigator-assessed RECIST 1.1-defined progression will be considered to have investigator-assessed progressive disease at the time of the initial progression event.

9.0 PRE-TREATMENT EVALUATION

9.1 Within 28 days of study entry

- Signed and dated informed consent.
- Physical Exam
- Updated evaluation of concurrent non-malignant diseases and recent medical therapy (within the thirty days prior to the evaluation).
- Imaging: CT scan of chest and abdomen/pelvis (MRI of abdomen/pelvis may be substituted).
- CT scan or MRI of the brain will be ordered if clinically indicated
- Plain films of bones/skeletal survey, and MRI of specific skeletal sites (such as spine, long bones, pelvic bones) will be ordered if clinically indicated
- Bone scan
- 12-lead ECG
- Serum hemoglobin electrophoresis
    - No need to repeat if previously done at any point during the patient's lifetime. However, records of the results need to be obtained if performed at an outside institution
- Collect fresh biopsy (up to 42 days prior), unless there is available frozen tissue in liquid nitrogen on an IRB approved correlated trial up to 6 weeks (42 days) prior to initiation of treatment on Day 1. These will be reviewed by the pathologist for confirmation of cancer.

9.2 Within 14 days (+/- 3 days) of study entry

- Confirmation of the eligibility of patients (see Section 4 for detailed Inclusion/Exclusion criteria)
- Demographics
- Baseline signs and symptoms
- Interim history
- Assessment of ECOG performance status, weight, temperature, resting systolic and diastolic blood pressure, heart rate, respiratory rate, oxygen (O_2) saturation by pulse oximetry
- Physical Examination
- Assessment of all concomitant medications and treatments taken
- Laboratory testing:
  - CBC with differential & platelets
    - Differentials include neutrophils, lymphocytes, monocytes, eosinophils and basophils
  - Chemistry panel including electrolytes (Na, K, Cl, CO_2), albumin, alkaline phosphatase, ALT, AST, calcium, LDH, total bilirubin, BUN, creatinine, phosphorus, and glucose.
  - INR/PTT
  - Hepatitis B and C screening tests (Hepatitis B surface antigen, Hepatitis B core antibody, Hepatitis C IgG)
    - If hepatitis C IgG is positive then active infection has to be confirmed by hepatitis C RNA testing.
  - T-SPOT or other interferon-gamma release assay to test for tuberculosis
  - Fasting lipid profile
    - Fasting blood sugar; for fasting blood sugar, patients should have nothing to eat or drink, except for water, for 8 hours leading up to the tests.
  - Serum amylase and lipase,
  - Serum free T4 and TSH.
  - Serum ACTH and Cortisol
  - Urinalysis: Gross examination including specific gravity, protein, glucose, and blood; microscopic examination including white blood cells / high power field (WBC/HPF), red blood cells / high power field (RBC/HPF) and any additional findings.
    - If urine protein ≥ 100 mg/dL then obtain random urine protein/creatinine ratio
  - Serum or urine pregnancy test for females of childbearing potential

9.3 Within 6 months (180 days) of study entry

- Doppler echocardiogram

Treatment can not start until at least 1 week after any minor surgical procedure, excluding placement of a vascular access device and core biopsies.

10.0 EVALUATION DURING TREATMENT

10.1 A cycle of treatment is defined as 3 weeks (+/- 3 days) during the combination phase of

nivolumab plus ipilimumab, and 4 weeks (+/- 3 days) during the maintenance phase of single-agent nivolumab. The following must be performed on Day 1 (+/- 3 days), of each course. If assessment and tests were completed within 7 days of Course 1 Day 1, procedures will not be repeated. Patients are required to come to MD Anderson at the beginning of every course.

- Interim history
- Assessment of ECOG performance status, weight, temperature, blood pressure, heart rate.
- Physical examination
- Assessment of all concomitant medications and treatments taken since the last assessment. Concurrent medications related to co-morbidity (e.g., hypertension, diabetes, etc.) will be recorded in the database. The name, dose, date start and stop (as accurately as possible) along with indication of the medication will be collected.
- Assessment of adverse events and tumor-related signs and symptoms
- Assessment of treatment related toxicities
- Laboratory testing:
  - CBC with differential & platelets
  - Chemistry panel including electrolytes (Na, K, Cl, CO_2), alkaline phosphatase, ALT, AST, calcium, total bilirubin, BUN, creatinine

10.2 The following labs will additionally be obtained at 6 weeks (42 days +/- 3 days), 12 weeks (84 days +/- 7 days), and every 8 weeks (56 days +/- 7 days) from the beginning of the therapy:

- Serum amylase and lipase
- Serum free T4 and TSH.
- Serum ACTH and Cortisol
- Urinalysis: Gross examination including specific gravity, protein, glucose, and blood; microscopic examination including white blood cells / high power field (WBC/HPF), red blood cells / high power field (RBC/HPF) and any additional findings.
  - If urine protein ≥ 100 mg/dL then obtain random urine protein/creatinine ratio

10.3 Imaging scans

(CT of chest and CT scan or MRI of abdomen/pelvis) will be performed to determine disease response at 6 weeks (42 days) (+/- 3 days) from Day 1 of the study, then at 12 weeks (84 days +/- 7 days), and then every 8 weeks (56 days +/- 7 days) thereafter, for as long as patients are receiving therapy on protocol A follow-up CT or MRI of the brain will only be ordered if clinically indicated. Confirmation of disease progression is recommended at a minimum of 4 weeks after the first progressive disease (PD) assessment, and especially in patients with a minimal increase in total measured tumor burden (as defined in section 11) over 20% during the flare time-window of the first 12 weeks of treatment, to account for expected delayed response. Patients who under cytoreductive nephrectomy at any point will have post-surgical, baseline staging studies with CT or MRI. Subsequently, restaging will be carried out every 8 weeks unless, in the investigators' opinion, follow-up intervals need to be shorter.

10.4 End of Treatment Evaluation:

Adverse events and tumor-related signs and symptoms will be assessed. All patients will be followed for irAEs on days 30 and 90 after the last dose of study treatment. For patients who develop toxicity related to study drug necessitating discontinuation of protocol therapy before restaging to assess tumor response, physicians treating these patients will make every effort to repeat the appropriate imaging studies if feasible and indicated to assess tumor response at the time the patients are taken off protocol treatment. If a possible delayed irAE is suspected, the patient will be requested to return to the Investigator's clinic or office for a physical examination and blood work, and other relevant laboratory assessments.
Assessment and tests were completed at EOT evaluation:

- Interim history
- Assessment of ECOG performance status, weight, temperature, resting systolic and diastolic blood pressure, heart rate, respiratory rate, oxygen (O_2) saturation by pulse oximetry
- Physical Examination
- Assessment of all concomitant medications and treatments taken since the last assessment

10.5 Long-term Follow-Up

Patients will be followed for survival every 3 months (90 days ± 1 month or 28 days) by record review or telephone correspondence.

11.0 STUDY CALENDAR

	**Pre-Study**	**Day 1 of each cycle (+/- 3 days)**	**6 weeks (42 days)(+/- 3 days) from day 1**	**12 weeks (84 days)(+/- 7 days) from day 1**	**After completion of combination phase**	**Every 8 weeks (56 days +/- 7 days) from day +1**	**End of treatment[t]**
Informed Consent	X[a]						
Confirmation of eligibility	X[b]						
Histologic diagnosis/confirmation	X[b]						
Medical History	X[a]						
Baseline Signs and Symptoms	X[b]						
Updated Evaluation of concurrent non-malignant diseases	X[c]						
Demographics	X[b]						
Interim History	X[b]	X					X
Physical Examination	X[a,b]	X					X
Imaging	X[f]		X[f,g]	X[f,g]		X[f,g]	X[f,g]
Plain films of bones/skeletal survey	X[a,h]						
Fresh Biopsy	X[d]						
Echocardiogram	X[e]						
Weight, temperature, blood pressure, heart rate and respiratory rate	X[b]	X					X
O_2 saturation	X[b]	X					X
CBC with differential & platelets	X[b,i]	X[i]	X[i]	X[i]		X[i]	
Chemistry Panel	X[b,j]	X[r]	X[j]	X[j]		X[j]	

	Pre-Study	Day 1 of each cycle (+/- 3 days)	6 weeks (42 days)(+/- 3 days) from day 1	12 weeks (84 days)(+/- 7 days) from day 1	After completion of combination phase	Every 8 weeks (56 days +/- 7 days) from day +1	End of treatment[t]
INR/PTT	X[b]						
Serum Free T4 + TSH	X[b]		X	X		X	
Serum ACTH and Cortisol	X[b]		X	X		X	
Urinalysis	X[b,k,l]		X[k,l]	X[k,l]		X[k,l]	
Pregnancy Test	X[b,m]						
Fasting Lipid Profile	X[b]						
Fasting blood sugar	X[b,n]						
Amylase and Lipase	X[b]		X	X		X	
Hepatitis B and C screening	X[b,o]						
Tuberculosis screening	X[b,q]						
Hemoglobin electrophoresis	X[a,p]						
12-Lead ECG	X[a]						
ECOG Performance Status	X[b]	X					X
Concomitant Medications	X[b]	X					X
Adverse Events		X					X[s]
Long Term Follow Up							X[u]

a. Within 28 days of study entry
b. Within 14 days (+/- 3 days) of study entry
c. Updated evaluation of concurrent non-malignant diseases and recent medical therapy (within the thirty days prior to the evaluation).
d. Fresh biopsy of the most accessible tumor site determined at the physician's discretion (up to 42 days prior)
e. During Pre-Study, must be within six months of study entry
f. CT scan of chest, CT scan or MRI of abdomen/pelvis at 6 weeks (42 days)(+/- 3 days) from study Day 1, then at 12 weeks (+/- 7 days) from study Day 1, and then every 8 weeks (56 days)(+/- 7 days) thereafter for as long as the patient is receiving therapy on protocol. Bone scan only at baseline (within 28 days of study entry), and can be repeated if clinically indicated; CT scan or MRI of the brain only if clinically indicated; any follow-up CT scan or MRI will be ordered if clinically indicated. Confirmation of disease progression is recommended at a minimum of 4 weeks after the first progressive disease (PD) assessment, and especially in patients with a minimal increase in total measured tumor burden (as defined in section 11) over 20% during the flare time-window of the first 12 weeks of treatment, to account for expected delayed response.

g. Patients who under cytoreductive nephrectomy at any point will have post-surgical, baseline staging studies with CT scan of chest, CT scan or MRI of abdomen/pelvis. Subsequently, restaging will be carried out every 8 weeks unless, in the investigators' opinion, follow-up intervals need to be shorter.
h. Plain films of bones/skeletal survey, and MRI of specific skeletal sites (such as spine, long bones, pelvic bones) will be ordered if clinically indicated
i. Differentials include neutrophils, lymphocytes, monocytes, eosinophils and basophils
j. Includes electrolytes (Na, K, Cl, Co2), albumin, alkaline phosphatase, ALT, AST, calcium, LDH, total bilirubin, BUN, creatinine, phosphorus, glucose
k. Gross examination including specific gravity, protein, glucose, and blood; microscopic examination including white blood cells / high power field (WBC/HPF), red blood cells / high power field (RBC/HPF) and any additional findings
l. If urine protein ≥ 100 mg/dL on urinalysis then obtain random urine protein/creatinine ratio
m. Only for women of childbearing potential. May be serum or urine
n. For fasting blood sugar, patients should have nothing to eat or drink, except for water, for 8 hours leading up to the tests.
o. Hepatitis B surface antigen, Hepatitis B core antibody, Hepatitis C IgG. If hepatitis C IgG is positive then active infection has to be confirmed by hepatitis C RNA testing
p. No need to repeat if previously done at any point during the patient's lifetime. However, records of the results need to be obtained if performed at an outside institution
q. T-SPOT or other interferon-gamma release assay to test for tuberculosis
r. Only includes electrolytes, alkaline phosphatase, ALT, AST, calcium, total bilirubin, BUN, and creatinine
s. All patients will be followed for irAEs on days 30 and 90 after finishing therapy.
t. Adverse events and tumor-related signs and symptoms will be assessed. For patients who develop toxicity related to study drug necessitating discontinuation of protocol therapy before restaging to assess tumor response, physicians treating these patients will make every effort to repeat the appropriate imaging studies if feasible and indicated to assess tumor response at the time the patients are taken off protocol treatment. If a possible delayed irAE is suspected, the patient will be requested to return to the Investigator's clinic or office for a physical examination and blood work, and other relevant laboratory assessments
u. Patients will be followed for survival every 3 months (90 days ± 1 month or 28 days) by record review or telephone correspondence. Patients who went off treatment without progression will also be followed for progression and date of starting next therapy, whichever comes first, on this same schedule

12.0 CRITERIA FOR RESPONSE OR PROGRESSION

12.1.1 Efficacy Assessments

Objective response rate (ORR) and Progression-free survival (PFS), each based on the RECIST 1.1 criteria, are two main efficacy assessments of this study.

Evaluation of response will follow the RECIST 1.1 criteria.[53,54] All tumor measurements must be recorded in centimeters.

- Target Lesions:

  All measurable lesions up to a maximum of 2 lesions per organ and 5 lesions in total, representative of all involved organs, should be identified as target lesions and recorded and measured at baseline. Target lesions should be selected on the basis of their size (those with the longest diameter) and their suitability for accurate repeated measurements (either by imaging techniques or clinically). A sum of the longest diameter for all target lesions will be calculated and reported as the baseline sum longest diameter. The baseline sum of longest diameters will be used as the reference by which the objective tumor response is characterized.

- Non-target Lesions:
  All other lesions (or sites of disease) up to 2 lesions per site and 5 lesions in total should be identified as non-target lesions and should also be recorded at baseline. Measurements of these lesions are not required, but the presence or absence of each should be noted throughout follow-up.

12.2 Evaluation of Target Lesions:

- Complete Response (CR):
  The disappearance of all target lesions.
- Partial Response (PR):
  At least a 30% decrease in the sum of the longest diameter of target lesions, taking as reference the baseline sum longest diameter.
- Progressive Disease:
  At least a 20% increase in the sum of the longest diameter of target lesions, taking as reference the smallest sum longest diameter recorded since the treatment started or the appearance of one or more new lesions.
- Stable Disease:
  Insufficient shrinkage to qualify for partial response, or insufficient increase to qualify for progressive disease, taking as reference the smallest sum longest diameter since the treatment started.

12. 3 Evaluation of Non-target Lesions:

- Complete Response:
  The disappearance of all non-target lesions.

- Incomplete Response/Stable Disease:
  The persistence of one or more non-target lesion(s)
- Progressive Disease:
  The appearance of one or more new lesions and/or unequivocal progression of existing non-target lesions.

12.4 Evaluation of Best Overall Response:

The best overall response is the best response recorded from the start of treatment until disease progression/recurrence (taking as reference for progressive disease the smallest measurements recorded since the treatment started).

Evaluation of Best Overall Response (RECIST)

Target Lesions	Non-target lesions	New lesions	Overall response
CR	CR	No	CR
CR	Incomplete response/SD	No	PR
PR	Non-PD	No	PR
SD	Non-PD	No	SD
PD	Any	Yes or no	PD
Any	PD	Yes or no	PD
Any	Any	Yes	PD

CR= complete response; PR= partial response; SD= stable disease; and PD= progressive disease

12. 5 irRECIST Terms:

- **Measurable Lesions:**
  Lesions that can be accurately measured in at least one dimension:
    - o ≥10 mm in the longest diameter by CT or MRI scan (or no less than double the slice thickness) for nonnodal lesions
    - o ≥15 mm in short axis for nodal lesions
    - o ≥10 mm caliper measurement by clinical exam
    - o ≥20 mm by chest X-ray

- **Non-Measurable Lesions:**
  All other lesions, including:
    - o Small lesions with longest diameter < 10 mm with conventional techniques, < 10 mm with spiral CT scan (or < 2 x the axial slice thickness)
    - o Pathological lymph nodes with ≥ 10 to <15 mm short axis
    - o Other types of lesions that are confidently felt to represent neoplastic tissue, but are difficult to measure in a reproducible manner, such as bone lesions, ascites, pleural/pericardial effusion, leptomeningeal metastases, cystic lesions, ill-defined abdominal masses, inflammatory breast disease, and lymphangitis cutis/pulmonis,

- **Measurable Disease:**
  The presence of at least one measurable lesion.

- **Target Lesions:**
  All measurable lesions up to a maximum of 2 lesions per organ and 5 lesions in total, representative of all involved organs, should be identified as target lesions and recorded and measured at baseline. Target lesions should be selected on the basis of their size (those with the longest diameter) and their suitability for accurate repeated measurements (either by imaging techniques or clinically).

- **Non-target Lesions:**
  All other lesions (or sites of disease) should be identified as non-target lesions and should also be recorded at baseline. There is no limit to the number of non-target lesions that can be recorded at baseline. Measurements of these lesions are not required, but the presence or absence of each should be noted throughout follow-up.

- **Measurable New Lesions:**
  Measurable new lesions are defined as all new lesions (up to a maximum of 2 lesions per organ, and 5 lesions in total, per timepoint) representative of all involved organs, meeting criteria as defined for baseline target lesion selection and meet the same minimum size requirements of 10 mm in long diameter and minimum 15 mm in short axis for new measurable lymph nodes. New measurable lesions are prioritized according to size, and the largest lesions are selected as new measured lesions.

- **Total Measured Tumor Burden (TMTB):**
  The TMTB is calculated as the sum of the longest diameter for all non-nodal target lesions, new non-nodal measurable lesions, and of the short axes of nodal target lesions and new nodal measurable lesions. The TMTB will be used as the reference from which to characterize the objective tumor response. In irRECIST, new measureable lesions (maximum 5, maximum 2 in one organ) are added to the TMTB at each follow-up and percent increase of TMTB over baseline will determine stable disease (SD) or progressive disease (PD).

12.5.1 Evaluation of Target Lesions per irRECIST:

- Complete Response (CR):
  Complete disappearance of all measurable and non-measurable lesions. Lymph nodes must decrease to < 10 mm in short axis

- Partial Response (PR):
  At least a 30% decrease in TMTB relative to baseline.
  .

- Progressive Disease (PD):
  At least a 20% increase, and minimum 5 mm absolute increase, in TMTB compared to the smallest TMTB recorded since the treatment started. Confirmation of progression is recommended at a minimum of 4 weeks after the first PD assessment, and especially in patients with a minimal TMTB %-increase over 20% during the flare time-window of the first 12 weeks of treatment, to account for expected delayed response.

- Stable Disease (SD):
  Insufficient shrinkage to qualify for PR (or CR), or insufficient increase to qualify for PD,

taking as reference the smallest TMTB recorded since the treatment started.

12.5.2 Evaluation of Non-Target Lesions:

- **Complete Response (CR):**
  Disappearance of all non-target lesions. All lymph nodes must be non-pathological in size (< 10 mm short axis).

- **Immune-related Non-CR/Non-PD**:
  Persistence of one or more non-target lesion(s) and/or maintenance of tumor marker level above the normal limits. This also includes non-target lesions that demonstrated unequivocal progression at the initial demonstration of PD but then stabilized or regressed on subsequent scans.

- **Immune-related Progressive Disease (irPD):**
  Unequivocal progression of existing non-target lesions on a disease assessment subsequent to initial demonstration of PD. Therefore, irPD by non-target lesions requires one of the following on a disease assessment >4 weeks after the initial demonstration of PD:
    - New unequivocal progression OR
    - Further unequivocal progression (if unequivocal progression present at initial PD)

13.0 CRITERIA FOR REMOVAL FROM PROTOCOL TREATMENT

- Progressive disease: Patients, who develop rapidly progressive disease (clinically or by RECIST 1.1) before the scheduled evaluations, may be taken off protocol treatment at the discretion of the investigator. Patients can continue to receive their assigned targeted agent, even if they develop progressive disease radiographically, as long as in the judgment of the treating physician, they are benefitting from their immune checkpoint therapy.
- Intercurrent illness that prevents continuation of treatment.
- Unacceptable adverse event(s), or delay of treatment for > 4 weeks due to treatment-related toxicity. Note: Patients who require emergency surgery (e.g. for appendectomy or because of trauma complications) or who require a procedure (e.g. Kyphoplasty/vertebroplasty) may remain on trial, even if administration of the immune checkpoint therapy is interrupted, as long as the immune checkpoint therapy is resumed within 4 weeks from date of interruption.
- Patient non-compliance with therapy.
- Decision of the patient to withdraw from the study
- Lost to follow-up
- Imprisonment or the compulsory detention for treatment of either a psychiatric or physical (e.g., infectious disease) illness.

The consequence of study withdrawal is that no new information will be collected from the withdrawn patient and added to the existing data or any database.

14.0 SAFETY ASSESSMENTS AND REPORTING REQUIREMENTS

14.1 Adverse Events (AEs)

All patients will be evaluated for safety. The safety parameters include all laboratory tests and hematological abnormalities, physical examination findings, and spontaneous reports of adverse events reported to the investigator by patients. An adverse event is any untoward or unfavorable medical occurrence in a human subject, including any abnormal sign (for example, abnormal physical exam or laboratory finding), symptom, or disease, temporally associated with the subject's participation in the research, whether or not considered related to the subject's participation in the research. All adverse events encountered after the patient has provided informed consent and until 90days after the last dose of study treatment t will be evaluated according to the NCI Common Toxicity Criteria (CTCAE) version 4.03. Prior treatment associated toxicities present at the time of informed consent but before study treatment initiation, will be recorded as baseline abnormalities and graded according to NCI CTCAE version 4.03 criteria. Adverse events will not be collected during the surgery or while the patient recovers from their surgery (4-6 weeks after surgery date) prior to initiating maintenance nivolumab.

Information about all adverse events, whether volunteered by the subject, discovered by investigator questioning, or detected through physical examination, laboratory test or other means, will be collected and reported as described in the data submission schedule.

The principal investigator (or physician designee) is responsible for verifying and providing source documentation for all adverse events and assigning the attribution for all adverse events for subjects enrolled.

Recommended Adverse Event Recording Guidelines

Attribution	**Grade 1**	**Grade 2**	**Grade 3**	**Grade 4**	**Grade 5**
Unrelated	Phase I	Phase I	Phase I<br>Phase II	Phase I<br>Phase II<br>Phase III	Phase I<br>Phase II<br>Phase III
Unlikely	Phase I	Phase I	Phase I<br>Phase II	Phase I<br>Phase II<br>Phase III	Phase I<br>Phase II<br>Phase III
Possible	Phase I<br>Phase II	Phase I<br>Phase II<br>Phase III	Phase I<br>Phase II<br>Phase III	Phase I<br>Phase II<br>Phase III	Phase I<br>Phase II<br>Phase III
Probable	Phase I<br>Phase II	Phase I<br>Phase II<br>Phase III	Phase I<br>Phase II<br>Phase III	Phase I<br>Phase II<br>Phase III	Phase I<br>Phase II<br>Phase III
Definitive	Phase I<br>Phase II	Phase I<br>Phase II<br>Phase III	Phase I<br>Phase II<br>Phase III	Phase I<br>Phase II<br>Phase III	Phase I<br>Phase II<br>Phase III

14.2 Serious Adverse Event Reporting (SAE)

An adverse event or suspected adverse event reaction is considered “serious” if, in the view of either the investigator or the IND Office, it results in any of the following outcomes:

- Death
- A life-threatening adverse drug experience – any adverse experience that places the patient, in the view of the initial reporter, at immediate risk of death from the adverse experience as it occurred. It does not include experience that, had it occurred in a more severe form, might have caused death.
- Inpatient hospitalized or prolongation of existing hospitalization.
- A persistent or significant incapacity or substantial disruption of the ability to conduct normal life functions.
- A congenital anomaly/birth defect.

Important medical events that may not result in death, be life-threatening, or require hospitalization may be considered a serious adverse drug experience when based upon appropriate medical judgment, they may jeopardize the patient or subject and may require medical or surgical_intervention to prevent one of the outcomes listed in this definition. Examples of such medical events include allergic bronchospasm requiring intensive treatment in an emergency room or at home, blood dyscrasias or convulsions that do not result in inpatient hospitalization, or the development of drug dependency or drug abuse (21 CFR 312.32).

- **Important medical events as defined above, may also be considered serious adverse events. Any important medical event can and should be reported as an SAE if deemed appropriate by the Principal Investigator or the IND Office.**
- All events occurring during the conduct of a protocol and meeting the definition of a SAE must be reported to the IRB in accordance with the timeframes and procedures outlined in “The University of Texas M. D. Anderson Cancer Center Institutional Review Board Policy for Investigators on Reporting Unanticipated Adverse Events for Drugs and Devices”. Unless stated otherwise in the protocol, all SAEs, expected or unexpected, must be reported to the IND Office, regardless of attribution (within 5 working days of knowledge of the event).
- **All life-threatening or fatal events**, that are unexpected, and related to the study drug, must have a written report submitted within 24 hours (next working day) of knowledge of the event to the Safety Project Manager in the IND Office.
- **Unless otherwise noted, the electronic SAE application (eSAE) will be utilized for safety reporting to the IND Office and MDACC IRB.**
- **Serious adverse events will be captured from the time of the first protocol-specific intervention, until 30 days after the last dose of drug, unless the participant withdraws consent. Serious adverse events must be followed until clinical recovery is complete and laboratory tests have returned to baseline, progression of the event has stabilized, or there has been acceptable resolution of the event.**
- **Additionally, any serious adverse events that occur after the 30 day time period that are related to the study treatment must be reported to the IND Office. This may include the development of a secondary malignancy.**

14.3 Reporting to FDA

- Serious adverse events will be forwarded to FDA by the IND MD Anderson (Safety Project Manager IND Office) according to 21 CFR 312.32.

It is the responsibility of the PI and the research team to ensure that serious adverse events are reported according to the Code of Federal Regulations, Good Clinical Practices, the protocol guidelines, the IND Office's guidelines, and Institutional Review Board policy.

14.4 Investigator Communication with Supporting Companies:

- SAEs will be reported on MedWatch Form 3500A, which can be accessed at: http://www.accessdata.fda.gov/scripts/medwatch/. The website will instruct you where to send the SAE forms.
- Following the subject's written consent to participate in the study, all SAEs, whether or not related to the BMS product associated with this study, must be collected, including those thought to be associated with protocol-specified procedures. SAEs must be recorded on the Solicited and Non-interventional Research AE/SAE Form and reported to BMS (or designee) within 24 hours/1 business day to comply with regulatory requirements. A form should be completed for any event where doubt exists regarding its status of seriousness. Although overdose and cancer are not always serious by regulatory definition, these events should be recorded on a form and reported to BMS within 24 hours/1 business day. **Investigators should report to the responsible regulatory authority as appropriate.**
- All SAEs must be reported by confirmed facsimile (fax) transmission or reported via electronic mail to:
    - SAE Email Address: Worldwide.Safety@BMS.com
    - SAE Facsimile Number: 1-609-818-3804
- If only limited information is initially available, follow-up reports may be required.
- For Comparator Drugs/Secondary Suspects (Concomitant Medications), all serious adverse experiences will be forwarded to the product manufacturer by the investigator.
- Any pregnancy that occurs during study participation should be reported. The pregnancy should be followed up to determine outcome, including spontaneous or voluntary termination, details of the birth, and the presence or absence of any birth defects, congenital abnormalities, or maternal and/or newborn complications. The investigator must immediately notify Worldwide.Safety@bms.com of this event via the Pregnancy Surveillance Form in accordance with SAE reporting procedures.

15.0 STATISTICAL CONSIDERATIONS / DATA ANALYSIS

15.1 Sample Size

Up to 30 patients will be enrolled on this trial. The selection of 30 patients is based on the feasibility of completing the trial within 6 years while retaining acceptable operating characteristics for the monitoring described below. One hypothesis test is planned comparing the historical response rate of 29% (13/45)[2] to the posterior distribution of this trial's response rate. This hypothesis will only be tested if the trial successfully accrues to 30 patients. Denote the probability of ORR in this trial by θ_R and the probability of ORR in the historical cohort by θ_H. If this trial has 12 or more responses, then $Pr(\theta_R>\theta_H \mid data)$ will be greater than 0.80, using posterior probabilities of $\theta_H \sim$ Beta(13,32) and $\theta_R \sim$ Beta(r+0.6, q+1.4) where r is the number of responders and q is the number of non-responders (q=30-r) and the prior for θ_R is beta(0.6, 1.4). Table 2 presents the probability of achieving at least 12 responses in 30 patients assuming the trial does not stop for toxicity. If the true ORR is 29% then we will have a 12% chance of falsely claiming that this combination is better than the historical ORR. If the true ORR is 46%, we will have an 80% chance of correctly claiming that this combination is better than the historical rate.

Table 2. The probability of concluding that this combination of Ipilimumab + Nivolumab has better ORR than historical patients, where better means $Pr(\theta_R>\theta_H \mid data)>0.80$ based on 1000 simulations.(Simulation code found in Appendix C)

True Response Rate	Probability of at least 12 responses
0.2	0.015
**0.29**	**0.117**
0.3	0.138
0.4	0.576
0.45	0.770
**0.46**	**0.801**
0.475	0.844
0.5	0.892

15.2 Trial Monitoring

Ongoing monitoring for safety and futility will be implemented based on the method of Thall (1995)[55] using cohorts of 10 patients each. Accrual will be held between cohorts until boundaries can be assessed for both toxicity and response. Calculations were performed in Multc Lean 2.1.

15.2.1 Safety monitoring

For trial monitoring and decisions about future trials, extreme toxicities (TOX) will be defined as any event requiring the patient to be removed from the trial according to section 8.1. A patient who voluntarily leaves the trial for any reason, including toxicity that does not meet the criteria in section 8.1 will not count as having a TOX. This will count as a TOX at any time during the study. For cohort monitoring, the most recent patients will be classified by their status at the time of cohort analysis, so the shortest follow-up time is expected to be 12 weeks. Denote the probability of TOX by θ_T. Our stopping rule is given by the following probability statement: $Pr(\theta_T > 0.30 \mid data) > 0.95$. That is, we will stop the trial if, at any time during the study, we determine that there

is more than an 95% chance that the TOX rate is more than 30%. Multc Lean version 2.1 was used to determine the stopping rules assuming a TOX rate for "standard therapy" of 0.30 and a prior θT ~ beta (0.6, 1.4) for the current study. The stopping boundaries for this toxicity rule are to terminate the trial if the number of patients with TOX compared to the number of patients having received treatment exceeds the limits in Table 3 below. The trial will stop at 30 patients, but if there are 14 or more TOX events by the end, then the combination will be too toxic for future trials at these doses in this patient population.

15.2.2 Futility monitoring

For trial monitoring, objective response rate (ORR) will be CR or PR by RECIST at 12 weeks. Denote the probability of ORR by θ_R. We assume $\theta_R \sim$ beta (0.5, 0.5). Our stopping rule is given by the following probability statement: $\Pr(\theta_R < 0.30 \mid \text{data}) > 0.90$. That is, we will stop entering patients if, at any time during the study, we determine that there is more than an 90% chance that the ORR is less than 30%, a constant rate that is the slightly above the expected response rate for these patients on other treatments. The stopping boundaries for this futility rule are to terminate enrollment on the trial if the number of overall responses compared to the number of patients enrolled who are evaluable does not meet the requirements of this table. Monitoring will be carried out once there are 10 patients who have been treated and evaluated. Patients who leave the study after receiving treatment but before the 12 week evaluation will be counted as non-responders and therefore evaluable for response. Patients who enroll but never receive treatment will be replaced.

15.3 Stopping rules and operating Characteristics

Table 3. Stopping Criteria for Excessive TOX or Insufficient Responses

Cohort Size	10	20	30*
Stop if there are this many patients (or fewer) with an objective response:	1	3	6
Stop if there are this many (or more) patients with TOX :	6	10	14

*Always stop with 30 patients, but if 6 or fewer patients respond or 14 or more have TOX, then this combination is not interesting for further investigation in these patients.

Table 4. The Operating Characteristics under Varying Toxicity and Response Rates

True Overall Toxicity Rate	True Overall Response Rate	Probability of Stopping Early	Probability of continuing after 10 patients	Median (25th %ile, 75th %ile)
0.10	0.10	0.90	0.26	10 (10, 20)
	0.20	0.51	0.62	20 (10, 30)
	0.30	0.19	0.85	30 (30, 30)
	0.40	0.05	0.95	30 (30, 30)
	0.50	0.01	0.99	30 (30, 30)
0.20	0.10	0.90	0.26	10 (10, 20)
	0.20	0.51	0.62	20 (10, 30)
	0.30	0.20	0.85	30 (30, 30)
	0.40	0.06	0.95	30 (30, 30)
	0.50	0.01	0.98	30 (30, 30)

**0.30**	0.10	0.91	0.25	10 (10, 20)
	0.20	0.55	0.59	20 (10, 30)
	**0.30**	**0.25**	**0.81**	**30 (20, 30)**
	0.40	0.12	0.91	30 (30, 30)
	0.50	0.09	0.94	30 (30, 30)
0.40	0.10	0.93	0.22	10 (10, 10)
	0.20	0.66	0.52	20 (10, 30)
	0.30	0.43	0.71	30 (10, 30)
	0.40	0.33	0.80	30 (20, 30)
	0.50	0.30	0.82	30 (20, 30)
0.50	0.10	0.96	0.16	10 (10, 10)
	0.20	0.82	0.39	10 (10, 20)
	0.30	0.70	0.53	20 (10, 30)
	0.40	0.65	0.59	20 (10, 30)
	0.50	0.63	0.62	20 (10, 30)

The Investigator is responsible for completing an efficacy/safety summary report, and submitting it to the IND Office Medical Affairs and Safety Group, for review and approval. This should be submitted after the first 10 evaluable patients per cohort complete 12 weeks of study treatment, and every 10 evaluable patients per cohort, thereafter. On every report submission, the information from previous reported patients will need to be updated.

A copy of the cohort summary should be placed in the Investigator's Regulatory Binder under "sponsor correspondence".

15.4 Analysis Plan:

Descriptive statistics will be presented. For discrete or categorical data including ORR and DCR, descriptive statistics will include tabulations of frequencies. For continuous data, summary statistics including *n*, mean, standard deviation, median, minimum and maximum will be computed. The estimate of the posterior ORR and its 95% credible interval will be estimated using the same prior as utilized in the futility monitoring rules. The posterior distribution for ORR of this trial will be compared to the historical data as cited in Section 15.1. The Kaplan-Meier method[56] will be utilized to display PFS, time to ORR, duration of response, and OS if more than 10 patients enroll. Patient adverse events and the number of patients required to be removed from the study will be tabulated by symptom grade and attribution to study drug. Continuous biomarker measures before and after treatment will be compared with paired t-tests, or non-parametric alternative as indicated. Association with ORR, CBR, or TOX events will be explored with logistic regression if there are at least 10 patients with and 10 patients without each event for the relevant measure. Association with time to event outcomes will be explored for any measure with at least 10 events.

<u>Toxicity</u>: Adverse effects that will be evaluated include, but are not limited to infections, renal toxicity, hepatic toxicity, and pulmonary toxicity. Methods of assessment will include monitoring blood counts, and performing laboratory tests as indicated by clinical signs and symptoms. Evidence of toxicity or adverse events will be recorded at all clinic visits. All observed adverse effects will be graded for all patients and the degree of association of each with therapy assessed.

16.0 DATA AND PROTOCOL MANAGEMENT

Protocol Compliance: All required interim and pretreatment data should be available, and the physician must assess tumor response and must provide a detailed description of toxicity, when appropriate. If dose modifications or treatment interruptions are necessary, the details must be carefully documented. Performance status must be documented at each toxicity assessment.

Data Capture: Data will be entered in the MD Anderson institutionally approved database(s). Registration data entry will occur prior to initiation of therapy. All eligibility criteria must be satisfied.

Accuracy of Data Collection: The MD Anderson Principal Investigator will be the final arbiter of response and toxicity, should a difference of opinion exist.

Monitoring: The trial will be monitored throughout the study with the assistance of the MD

Anderson IND office. The study will be performed in accordance with ethical principles and follow federal, institutional, and departmental policy and or regulation. Every effort will be made to maintain patient confidentiality in accordance with federal regulation and institutional policy.

Chain of custody of biological samples: The chain of custody will be maintained for all samples throughout to completion and close out of the trial. A record of storage location will be documented. Records will be available for monitoring and audit as requested.

17.0 REFERENCES

1. Davis CJ, Jr., Mostofi FK, Sesterhenn IA. Renal medullary carcinoma. The seventh sickle cell nephropathy. The American journal of surgical pathology 1995;19:1-11.
2. Shah AY, Karam JA, Malouf GG, et al. Management and Outcomes of Patients with Renal Medullary Carcinoma: A Multi-Center Collaborative Study BJU International 2016; accepted, in print.
3. Iacovelli R, Modica D, Palazzo A, Trenta P, Piesco G, Cortesi E. Clinical outcome and prognostic factors in renal medullary carcinoma: A pooled analysis from 18 years of medical literature. Canadian Urological Association journal = Journal de l'Association des urologues du Canada 2015;9:E172-7.
4. Shuch B, Amin A, Armstrong AJ, et al. Understanding pathologic variants of renal cell carcinoma: distilling therapeutic opportunities from biologic complexity. European urology 2015;67:85-97.
5. Amin MB, Smith SC, Agaimy A, et al. Collecting duct carcinoma versus renal medullary carcinoma: an appeal for nosologic and biological clarity. The American journal of surgical pathology 2014;38:871-4.
6. Siegel RL, Miller KD, Jemal A. Cancer statistics, 2016. CA: a cancer journal for clinicians 2016;66:7-30.
7. Wright JL, Risk MC, Hotaling J, Lin DW. Effect of collecting duct histology on renal cell cancer outcome. The Journal of urology 2009;182:2595-9.
8. Tokuda N, Naito S, Matsuzaki O, et al. Collecting duct (Bellini duct) renal cell carcinoma: a nationwide survey in Japan. The Journal of urology 2006;176:40-3; discussion 3.
9. Alvarez O, Rodriguez MM, Jordan L, Sarnaik S. Renal medullary carcinoma and sickle cell trait: A systematic review. Pediatric blood & cancer 2015;62:1694-9.
10. Dimashkieh H, Choe J, Mutema G. Renal medullary carcinoma: a report of 2 cases and review of the literature. Archives of pathology & laboratory medicine 2003;127:e135-8.
11. Marsh A, Golden C, Hoppe C, Quirolo K, Vichinsky E. Renal medullary carcinoma in an adolescent with sickle cell anemia. Pediatric blood & cancer 2014;61:567.
12. Ojodu J, Hulihan MM, Pope SN, Grant AM, Centers for Disease C, Prevention. Incidence of sickle cell trait--United States, 2010. MMWR Morbidity and mortality weekly report 2014;63:1155-8.
13. Grant AM, Parker CS, Jordan LB, et al. Public health implications of sickle cell trait: a report of the CDC meeting. American journal of preventive medicine 2011;41:S435-9.
14. Loukopoulos D. Haemoglobinopathies in Greece: prevention programme over the past 35 years. The Indian journal of medical research 2011;134:572-6.
15. Guler E, Garipardic M, Dalkiran T, Davutoglu M. Premarital screening test results for beta-thalassemia and sickle cell anemia trait in east Mediterranean region of Turkey. Pediatric hematology and oncology 2010;27:608-13.
16. Shrikhande AV, Arjunan A, Agarwal A, et al. Prevalence of the beta(S) gene among scheduled castes, scheduled tribes and other backward class groups in Central India. Hemoglobin 2014;38:230-5.
17. Salamah MM, Mallouh AA, Hamdan JA. Acute splenic sequestration crises in Saudi children with sickle cell disease. Annals of tropical paediatrics 1989;9:115-7.
18. Sickle-cell anaemia: report by the Secretariat. 59th World Health Assembly April 2, 2006.
19. Margol AS, Judkins AR. Pathology and diagnosis of SMARCB1-deficient tumors. Cancer genetics 2014;207:358-64.
20. Cheng JX, Tretiakova M, Gong C, Mandal S, Krausz T, Taxy JB. Renal medullary

carcinoma: rhabdoid features and the absence of INI1 expression as markers of aggressive behavior. Modern pathology : an official journal of the United States and Canadian Academy of Pathology, Inc 2008;21:647-52.
21. Rao P, Tannir NM, Tamboli P. Expression of OCT3/4 in renal medullary carcinoma represents a potential diagnostic pitfall. The American journal of surgical pathology 2012;36:583-8.
22. Shi Z, Zhuang Q, You R, Li Y, Li J, Cao D. Clinical and computed tomography imaging features of renal medullary carcinoma: A report of six cases. Oncology letters 2016;11:261-6.
23. Escudier B, Pluzanska A, Koralewski P, et al. Bevacizumab plus interferon alfa-2a for treatment of metastatic renal cell carcinoma: a randomised, double-blind phase III trial. Lancet 2007;370:2103-11.
24. Motzer RJ, Escudier B, McDermott DF, et al. Nivolumab versus Everolimus in Advanced Renal-Cell Carcinoma. The New England journal of medicine 2015;373:1803-13.
25. Escudier B, Bellmunt J, Negrier S, et al. Phase III trial of bevacizumab plus interferon alfa-2a in patients with metastatic renal cell carcinoma (AVOREN): final analysis of overall survival. J Clin Oncol 2010;28:2144-50.
26. Bracarda S, Bellmunt J, Melichar B, et al. Overall survival in patients with metastatic renal cell carcinoma initially treated with bevacizumab plus interferon-alpha2a and subsequent therapy with tyrosine kinase inhibitors: a retrospective analysis of the phase III AVOREN trial. BJU Int 2011;107:214-9.
27. Rini BI, Halabi S, Rosenberg JE, et al. Bevacizumab plus interferon alfa compared with interferon alfa monotherapy in patients with metastatic renal cell carcinoma: CALGB 90206. J Clin Oncol 2008;26:5422-8.
28. Rini BI, Halabi S, Rosenberg JE, et al. Phase III trial of bevacizumab plus interferon alfa versus interferon alfa monotherapy in patients with metastatic renal cell carcinoma: final results of CALGB 90206. J Clin Oncol 2010;28:2137-43.
29. Keir ME, Butte MJ, Freeman GJ, Sharpe AH. PD-1 and its ligands in tolerance and immunity. Annu Rev Immunol 2008;26:677-704.
30. Nishimura H, Nose M, Hiai H, Minato N, Honjo T. Development of lupus-like autoimmune diseases by disruption of the PD-1 gene encoding an ITIM motif-carrying immunoreceptor. Immunity 1999;11:141-51.
31. Larkin J, Chiarion-Sileni V, Gonzalez R, et al. Combined Nivolumab and Ipilimumab or Monotherapy in Untreated Melanoma. The New England journal of medicine 2015;373:23-34.
32. Iwai Y, Terawaki S, Honjo T. PD-1 blockade inhibits hematogenous spread of poorly immunogenic tumor cells by enhanced recruitment of effector T cells. Int Immunol 2005;17:133-44.
33. Nomi T, Sho M, Akahori T, et al. Clinical significance and therapeutic potential of the programmed death-1 ligand/programmed death-1 pathway in human pancreatic cancer. Clin Cancer Res 2007;13:2151-7.
34. Iwai Y, Ishida M, Tanaka Y, Okazaki T, Honjo T, Minato N. Involvement of PD-L1 on tumor cells in the escape from host immune system and tumor immunotherapy by PD-L1 blockade. Proceedings of the National Academy of Sciences of the United States of America 2002;99:12293-7.
35. Thompson RH, Gillett MD, Cheville JC, et al. Costimulatory molecule B7-H1 in primary and metastatic clear cell renal cell carcinoma. Cancer 2005;104:2084-91.
36. Topalian SL, Hodi FS, Brahmer JR, et al. Safety, activity, and immune correlates of anti-PD-1 antibody in cancer. The New England journal of medicine 2012;366:2443-54.
37. Hodi FS, O'Day SJ, McDermott DF, et al. Improved survival with ipilimumab in patients with metastatic melanoma. The New England journal of medicine 2010;363:711-23.

38. McDermott DF DC, Snzol M ea. Clinical activity and safety of anti-PD-1 (BMS-936558, MDX-1106) in patients with previously treated metastatic renal cell carcinoma (mRCC). J Clin Oncol 2012;30.
39. Ipilimumab IB. 2012 Aug-31. v15.
40. Weber JS, Kahler KC, Hauschild A. Management of immune-related adverse events and kinetics of response with ipilimumab. J Clin Oncol 2012;30:2691-7.
41. A Phase 2 Open-label Study of Single Agent Ipilimumab for the Treatment of IL-2 Refractory or IL-2 Ineligible Patients with Stage IV Renal Cancer; NCT00057889; Final Clinical Study Report 930025954 v1.0. (28-January-2008).
42. Yang JC, Hughes M, Kammula U, et al. Ipilimumab (anti-CTLA4 antibody) causes regression of metastatic renal cell cancer associated with enteritis and hypophysitis. Journal of immunotherapy 2007;30:825-30.
43. Wolchok JD, Kluger H, Callahan MK, et al. Nivolumab plus ipilimumab in advanced melanoma. The New England journal of medicine 2013;369:122-33.
44. Hellmann MD, Gettinger SN, Goldman JW, et al. CheckMate 012: Safety and efficacy of first-line (1L) nivolumab (nivo; N) and ipilimumab (ipi; I) in advanced (adv) NSCLC. J Clin Oncol 2016;34:(suppl; abstr 3001).
45. Hammers H, Plimack ER, Infante JR, et al. Updated results from a phase I study of nivolumab (Nivo) in combination with ipilimumab (Ipi) in metastatic renal cell carcinoma (mRCC): The CheckMate 016 study. Annals of Oncology 2016;27:1062P-P.
46. Hakimi AA, Koi PT, Milhoua PM, et al. Renal medullary carcinoma: the Bronx experience. Urology 2007;70:878-82.
47. Maroja Silvino MC, Venchiarutti Moniz CM, Munhoz Piotto GH, Siqueira S, Galapo Kann A, Dzik C. Renal medullary carcinoma response to chemotherapy: a referral center experience in Brazil. Rare tumors 2013;5:e44.
48. Beckermann KA, Jolly PC, Kim JU, et al. Clinical and immunologic correlates of response to PD-1 blockade in a patient with metastatic renal medullary carcinoma. Journal for ImmunoTherapy of Cancer 2017;5:1.
49. Gao J, Shi LZ, Zhao H, et al. Loss of IFN-gamma Pathway Genes in Tumor Cells as a Mechanism of Resistance to Anti-CTLA-4 Therapy. Cell 2016;167:397-404 e9.
50. Zhao X, Ivaturi V, Gopalakrishnan M, et al. A model-based exposure-response (E-R) assessment of a nivolumab (NIVO) 4-weekly (Q4W) dosing schedule across multiple tumor types. 2017 AACR Annual Meeting. Washington, DC 2017.
51. Martin-Algarra S, Haanen JB, Horak C, et al. Safety of reduced infusion times for nivolumab plus ipilimumab (N + I) and nivolumab alone (N) in advanced melanoma. Annals of Oncology 2016;27:1125P-P.
52. Wolchok J, Hoos A, O'Day S, et al. Guidelines for the evaluation of immune therapy activity in solid tumors: immune-related response criteria. Clin Cancer Res 2009;15:7412-19.
53. Bohnsack O, Hoos A, Ludajic K. Adaptation of the immune related response criteria: irRECIST. Annals of Oncology 2014;25(suppl_4):iv361-iv72.
54. Henze J, Maintz D, Persigehl T. RECIST 1.1, irRECIST 1.1, and mRECIST: How to Do. Current Radiology Reports 2016;4:48.
55. Thall PF, Simon RM, Estey EH. Bayesian sequential monitoring designs for single-arm clinical trials with multiple outcomes. Statistics in medicine 1995;14:357-79.
56. Kaplan EL, Meier P. Nonparametric estimator from incomplete observations. . J Am Stat Assoc 1958;53:457-81.

Appendix 1: Tests to Be Performed on MD Anderson Companion Trial 2014-0938

	Pre-Study	Day 1 of each cycle (+/- 3 days)	6 weeks (42 days)(+/- 3 days) from day 1	12 weeks (84 days)(+/- 7 days) from day1	After completion of combination phase	End of treatment
**Procedures**						
Blood for correlative studies [a, f, g, h]	X[b, i]		X	X	X	X
Tissue Sample [a, e, h, j]					X[c]	X[d]

a. Variations, alterations, and deletions will not be considered deviations from the protocol
b. Within 14 days (+/- 3 days) of study entry
c. After completion of the combination phase (nivolumab + ipilimumab) of therapy: collect fresh biopsy to assess treatment response to combination therapy. Fresh biopsy is not necessary in patients who will undergo cytoreductive nephrectomy after completion of the combination phase of therapy.
d. In patients taken off therapy due to disease progression, collect fresh biopsy to assess causes of disease progression.
e. All biopsies will be performed provided there is technically biopsiable tumor, and it is safe for patient to undergo biopsy. Tumor tissue will be obtained pre-treatment (baseline) up to 6 weeks (42 days) prior to initiation of treatment on Day 1, after completion of the combination therapy (nivolumab/ipilimumab) phase, and upon disease progression.
f. Blood samples (serum and peripheral blood mononuclear cells) for Immune Testing and Correlative Studies
g. Up to one hundred (150) ml of blood will be drawn at the visits outlined. These samples will be labeled with confidential identification numbers and provided to the Immunotherapy platform for immunological assessments. A detailed laboratory manual highlighting the blood collection and processing procedures will be prepared. All blood collection will be compliant with institutional safety standards and will not exceed the maximum blood draw per venipuncture policy.
h. In addition, another MD Anderson IRB-approved protocol, separate from the aforementioned immunotherapy analyses, will be used to investigate in the blood and tumor tissues, by protein assay methods, a panel of cytokines, chemokines, and other relevant angiogenic factors, biological receptors, and ligands.
i. The patient will not be required to undergo a second biopsy procedure prior to starting study therapy (screening) if an archival tumor tissue block or fresh frozen tissue in liquid nitrogen from up to 60 days prior to initiation of treatment is available. These will be reviewed by the pathologist for confirmation of cancer.
j. Fresh biopsy of the most accessible tumor site determined at the physician's discretion (up to 42 days prior)
