# Supplementary material for: Nivolumab plus ipilimumab induce hyper-progression in renal medullary carcinoma: results of a phase II trial and preclinical evidence
Source: Nat Commun. 2025 Nov 25;16:10474. doi: 10.1038/s41467-025-65462-z (PMC12647744; doi:10.1038/s41467-025-65462-z)
Supplement: Supplementary file 2 — Reporting Summary [file 41467_2025_65462_MOESM2_ESM.pdf]

nature portfolio

Corresponding author(s): Pavlos Msaouel, Giannicola Genovese, Linghua Wang, Jianjun Gao

Last updated by author(s): Sep 8, 2025

Reporting Summary

Nature Portfolio wishes to improve the reproducibility of the work that we publish. This form provides structure for consistency and transparency in reporting. For further information on Nature Portfolio policies, see our Editorial Policies and the Editorial Policy Checklist.

Statistics

For all statistical analyses, confirm that the following items are present in the figure legend, table legend, main text, or Methods section.

n/a	Confirmed	
☐	☒	The exact sample size (*n*) for each experimental group/condition, given as a discrete number and unit of measurement
☐	☒	A statement on whether measurements were taken from distinct samples or whether the same sample was measured repeatedly
☐	☒	The statistical test(s) used AND whether they are one- or two-sided<br>*Only common tests should be described solely by name; describe more complex techniques in the Methods section.*
☒	☐	A description of all covariates tested
☒	☐	A description of any assumptions or corrections, such as tests of normality and adjustment for multiple comparisons
☐	☒	A full description of the statistical parameters including central tendency (e.g. means) or other basic estimates (e.g. regression coefficient) AND variation (e.g. standard deviation) or associated estimates of uncertainty (e.g. confidence intervals)
☐	☒	For null hypothesis testing, the test statistic (e.g. *F*, *t*, *r*) with confidence intervals, effect sizes, degrees of freedom and *P* value noted<br>*Give P values as exact values whenever suitable.*
☐	☒	For Bayesian analysis, information on the choice of priors and Markov chain Monte Carlo settings
☒	☐	For hierarchical and complex designs, identification of the appropriate level for tests and full reporting of outcomes
☒	☐	Estimates of effect sizes (e.g. Cohen's *d*, Pearson's *r*), indicating how they were calculated

Our web collection on statistics for biologists contains articles on many of the points above.

Software and code

Policy information about availability of computer code

Data collection	Prometheus informatics system
Data analysis	SAS Version 9.4, Stata Version 17, GraphPad Prism Version 10

For manuscripts utilizing custom algorithms or software that are central to the research but not yet described in published literature, software must be made available to editors and reviewers. We strongly encourage code deposition in a community repository (e.g. GitHub). See the Nature Portfolio guidelines for submitting code & software for further information.

Data

Policy information about availability of data

All manuscripts must include a data availability statement. This statement should provide the following information, where applicable:

- Accession codes, unique identifiers, or web links for publicly available datasets
- A description of any restrictions on data availability
- For clinical datasets or third party data, please ensure that the statement adheres to our policy

The trial protocol is available with this submission as a Supplementary Note in the Supplementary Information file. All processed sequencing data (scRNA-seq and bulk RNA-seq) generated in this study have been deposited in the NCBI Gene Expression Omnibus (GEO) under accession code GSE256326 (https://www.ncbi.nlm.nih.gov/geo/query/acc.cgi?acc=GSE256326). Raw sequencing data are protected and are not available due to data privacy laws. Source data are provided with this paper. Requests to access data should be forwarded to the corresponding authors at PMsaouel@mdanderson.org and/or ggenovese@mdanderson.org and/or jgao1@mdanderson.org and/or lwang22@mdanderson.org. All requests for data and materials will be promptly reviewed to

verify whether the request is subject to any intellectual property or confidentiality obligations and responded to within 2-4 weeks. Any data and materials that can be shared will be released via a Material Transfer Agreement. The remaining data are available within the Article, Supplementary Information or Source Data file. Codes for analysis of scRNA-seq and bulk RNA-seq data, as well as for generating Figure 2 and Supplementary Figures S2–S8 and S15–S18 are available at GitHub (https://github.com/XinmiaoYan/RMC_hyperprogression) with the version used in this manuscript linked to Zenodo (https://doi.org/10.5281/zenodo.16995867)

Research involving human participants, their data, or biological material

Policy information about studies with human participants or human data. See also policy information about sex, gender (identity/presentation), and sexual orientation and race, ethnicity and racism.

Reporting on sex and gender	Findings apply to both sexes. Sex was not considered in study design; both males and females were eligible for study. Sex was determined based on self-reporting.<br>For the enrolled population on trial protocol (N=10)<br>Sex, n (%)<br>Male, 7 (70)<br>Female, 3 (30)<br>See manuscript for details.
Reporting on race, ethnicity, or other socially relevant groupings	Findings apply to all ethnicities. Ethnicity was not considered in study design; all ethnicities were eligible for study. Ethnicity was determined based on self-reporting.<br>For the enrolled population on trial protocol (N=10)<br>Ethnicity, n (%)<br>White, 1 (10)<br>Black, 8 (80)<br>Hispanic or Latino 1 (10)<br>See manuscript for details.
Population characteristics	Eligible patients were ≥18 years old with locally advanced or metastatic RMC confirmed to be negative for SMARCB1 by Clinical Laboratory Improvement Amendments (CLIA) certified immunohistochemistry (IHC) using purified mouse anti-BAF47 Clone 25/BAF47 (BD Biosciences). Sickle cell status was determined by hemoglobin electrophoresis for all patients enrolled with a diagnosis of RMC. Patients with RMC without sickle hemoglobinopathies were also eligible. Patients had an Eastern Cooperative Oncology Group (ECOG) performance status score of 0, 1, or 2, as well as measurable disease by the Response Evaluation Criteria in Solid Tumors (RECIST version 1.1), and could either naïve for any previous systemic treatment or have had any number of prior systemic therapies. However, patients must not have received prior anticancer therapy with anti-PD1, anti-PD-L1, or anti-CTLA-4 immune checkpoint inhibitors. While any clinical trial may be subject to selection biases, several factors mitigate this concern in the present study. RMC is an extremely rare and uniformly lethal malignancy, with limited therapeutic options. As such, patients and treating physicians are highly motivated to participate in investigational studies, reducing the likelihood of self-selection based on favorable prognosis. Importantly, the enrolled cohort was demographically and clinically representative of established RMC populations, including the predominance of young Black male patients with sickle cell trait and primary tumors arising from the right kidney. Therefore, while the small sample size inherent to this rare disease setting is a limitation, we do not anticipate systematic bias that would meaningfully alter the generalizability of our findings.
Recruitment	Patients were recruited on this investigator-initiated clinical trial (NCT03274258) medical oncologists who treat renal medullary carcinoma at MD Anderson. The total number of patients enrolled was 10. All patients provided written informed consent to participate based on the principles of the Declaration of Helsinki.
Ethics oversight	This investigator-initiated clinical trial (NCT03274258) was approved by the Institutional Review Board (IRB) of MD Anderson (protocol 2017-0201). Patient samples for correlative studies were approved by the same IRB and MD Anderson protocol.

Note that full information on the approval of the study protocol must also be provided in the manuscript.

Field-specific reporting

Please select the one below that is the best fit for your research. If you are not sure, read the appropriate sections before making your selection.

[x] Life sciences [ ] Behavioural & social sciences [ ] Ecological, evolutionary & environmental sciences

For a reference copy of the document with all sections, see nature.com/documents/nr-reporting-summary-flat.pdf

Life sciences study design

All studies must disclose on these points even when the disclosure is negative.

Sample size	The trial was originally planned to enroll up to 30 patients based on the feasibility of completing the trial within 3 years while retaining acceptable operating characteristics for the monitoring described. One hypothesis test was planned to compare the historical response rate of 29% (13/45) to the posterior distribution of this trial's response rate. This hypothesis was only planned to be tested if the trial successfully accrued to 30 patients. Denote the probability of objective response rate (ORR) in this trial by θR and the probability of ORR in the historical cohort by θH. If this trial had 12 or more responses, then Pr(θR>θH | data) would be greater than 0.80, using posterior probabilities of θH~ Beta(13,32) and θR~ Beta(r+0.6, q+1.4) where r is the number of responders and q is the number of non-responders (q=30-r) and the prior for θR is beta(0.6, 1.4).

Data exclusions	All enrolled patients who started treatment on trial were included in the analysis. Correlative data was included based on tissue/blood sample availability and passing quality control metrics.
Replication	For the clinical study, no efforts to verify reproducibility were attempted. See sample size section for rationale on target patient enrollment. For correlative studies, efforts were made to identify trends across patients in the study. All preclinical experiments were performed with at least two or more replicates. Key experiments were performed in independent biological replicates, using both technical repeats and orthogonal validation approaches (e.g., in vitro mechanistic assays and co-clinical in vivo studies). All main experimental findings were successfully replicated across these independent datasets and models. No major findings failed replication.
Randomization	Allocation was not random as this was a single arm study.
Blinding	Blinding was not applicable to the clinical trial as it was a single arm study. All preclinical studies were planned and performed to ensure that each experiment contains all groups and appropriate controls. Investigators were not blinded to group allocation during data collection and/or analysis.

Reporting for specific materials, systems and methods

We require information from authors about some types of materials, experimental systems and methods used in many studies. Here, indicate whether each material, system or method listed is relevant to your study. If you are not sure if a list item applies to your research, read the appropriate section before selecting a response.

Materials & experimental systems

n/a	Involved in the study	
☐	☒	Antibodies
☐	☒	Eukaryotic cell lines
☒	☐	Palaeontology and archaeology
☐	☒	Animals and other organisms
☐	☒	Clinical data
☒	☐	Dual use research of concern
☒	☐	Plants

Methods

n/a	Involved in the study	
☒	☐	ChIP-seq
☐	☒	Flow cytometry
☒	☐	MRI-based neuroimaging

Antibodies

Antibodies used	Supplementary Tables S14-S17 list all antibodies used in this study for immunohistochemistry, Western blot protein analysis, multiplex immunofluorescence for automated staining, and multiplex immunofluorescence for manual staining
Validation	All antibodies used in this study were validated by their manufacturer with validation details available at the manufacturers' websites. For each antibody, the catalogue number and clone information are provided in the Methods section and can be used to access the manufacturer's validation data.

Eukaryotic cell lines

Policy information about cell lines and Sex and Gender in Research

Cell line source(s)	The cell lines used in this study (MSRT1 and DOHH2) were generated at The University of Texas MD Anderson Cancer Center
Authentication	Authentication was confirmed by STR typing
Mycoplasma contamination	All cell lines tested negative for mycoplasma contamination
Commonly misidentified lines (See ICLAC register)	N/A

Animals and other research organisms

Policy information about studies involving animals; ARRIVE guidelines recommended for reporting animal research, and Sex and Gender in Research

Laboratory animals	6–8-week-old CB-17 SCID mice (Taconic), C57BL/6 mice, Townes sickle cell trait mouse crossed with the Rosa26-Cas9 knock-in mouse strain that had been kept on a C57BL/6J pure background, Sprague Dawley (SD) rats, male beagle dogs, male cynomolgus monkeys.<br><br>Mouse pharmacokinetic studies of IACS-16898: male C57BL/6 mice (9 for IV and 9 for PO), 6-8 weeks, weighing 20−30 g were used for studies. Food and water were available to all of the animals ad libitum.<br><br>Rat pharmacokinetic studies of IACS-16898: male rats (SD strain, 3 for IV and 3 for PO), 6-8 weeks, weighing 200−300 g were used for studies. The animals were fasted overnight and fed 4 h postdose. Water was available ad libitum for all of the animals.

	Dog pharmacokinetic studies of IACS-16898: Male Beagle dogs (3 for IV and 3 for PO), 3-36 months, weighing 7−10 kg were used for studies. The animals were fasted overnight and fed 4 h postdose.<br><br>Monkey pharmacokinetic studies of IACS-16898: Male Cynomolgus monkeys (3 for IV and 3 for PO), 3-5 years, weighing 3−5 kg were used for studies. The animals were fasted overnight and fed 4 h postdose.
Wild animals	N/A
Reporting on sex	Experiments were performed in male and female animals as reported in the manuscript
Field-collected samples	N/A
Ethics oversight	MD Anderson IACUC protocols 00000884-RN04 and 00001158

Note that full information on the approval of the study protocol must also be provided in the manuscript.

Clinical data

Policy information about clinical studies

All manuscripts should comply with the ICMJE guidelines for publication of clinical research and a completed CONSORT checklist must be included with all submissions.

Clinical trial registration	NCT03274258
Study protocol	Available with the manuscript as a Supplementary Note
Data collection	A total of 10 patients with renal medullary carcinoma were enrolled on the clinical trial between June 2018 and December 2020
Outcomes	The primary objective was to determine the objective response rate (ORR) of patients with locally advanced or metastatic renal medullary carcinoma treated with combination of nivolumab plus ipilimumab. ORR is defined as the proportion of patients with a best response of complete response (CR) or partial response (PR) by the RECIST 1.1 criteria recorded between Day 1 of the study and the date of objectively documented progression per RECIST 1.1 or the date of subsequent anti-cancer therapy, whichever occured first. The goal was to significantly improve the ORR compared with the historical ORR of 29% achieved using conventional cytotoxic chemotherapies. Secondary objectives included determining the efficacy and safety of the combination of nivolumab plus ipilimumab in patients with RMC with efficacy measured by overall survival (OS), progression-free survival (PFS), time to ORR, duration of response, and the disease control rate (DCR).

Plants

Seed stocks	N/A
Novel plant genotypes	N/A
Authentication	N/A

Flow Cytometry

Plots

Confirm that:

- [x] The axis labels state the marker and fluorochrome used (e.g. CD4-FITC).
- [x] The axis scales are clearly visible. Include numbers along axes only for bottom left plot of group (a 'group' is an analysis of identical markers).
- [ ] All plots are contour plots with outliers or pseudocolor plots.
- [x] A numerical value for number of cells or percentage (with statistics) is provided.

Methodology

Sample preparation	Samples were peripheral blood mononuclear cells (PBMCs) from patients enrolled on the clinical trial. Prior to staining, cryopreserved PBMCs were thawed in a water bath at 37°C and washed twice in thawing media (RPMI 1640 and 10% inactivated FBS) containing 1:10,000 Pierce Universal Nuclease (Thermo Scientific, Waltham, MA, USA). The cells were assessed for post-thaw counts and viability using an automated cell counter (Nexcelom Bioscience, Lawrence, MA, USA). 2 × 106 cells from each sample were aliquoted for CyTOF staining. For viability staining, cells were stained with Cell-ID cisplatin 198Pt (Standard BioTools) at a final concentration of 5 µM for 5 min at RT. Prior to antibody staining, samples were incubated with Human TruStain FcX (Biolegend, San Diego, CA, USA) for 10 min at RT for Fc-receptor blocking. To eliminate sample-

	specific staining variation, all samples were barcoded and then stained antibodies, processed and acquired as one multiplexed sample. Samples were then barcoded using a 20-plex CD45 Live-cell barcoding kit (Standard BioTools) for 30 min at RT. Each sample was washed twice with Maxpar Cell Staining Buffer after incubation with different barcodes, and all samples were combined into one tube. Next, cells were stained for 30 minutes at RT with the cocktail of surface anitbodies. After the surface stain incubation, samples were washed twice using Maxpar Cell Staining Buffer, then fixed and permeabilized with Foxp3/Transcription Factor Staining Buffer Set (eBioscience) and intracellular staining was performed for 45 minutes at RT. The staining was followed by washing twice in 1X Perm Wash and fixed with 1.6% paraformaldehyde (Thermo Fisher) for 10 min at RT. Afterwards, the intercalation solution was prepared by adding Cell-ID Intercalator-Ir into Maxpar Fix & Perm Buffer (Standard BioTools) to a final concentration of 41.6 nM (a 3000× dilution of the 125 µM stock solution. After second fixation, the cells were resuspended with the intercalation solution and incubated overnight at 4°C. Immediately prior to data acquisition, samples were then washed with Maxpar Cell Staining Buffer and then with subsequent washes in Maxpar Water (Standard BioTools) to remove buffer salts. Next, the cells were resuspended at a concentration of 1 × 106 cells/mL in Maxpar Water containing a 1:10 dilution of EQ Four Element Calibration Beads (Standard BioTools) and filtered through a 35 µm nylon mesh filter cap (Corning, Falcon).
Instrument	The samples were acquired on a Helios Mass Cytometer equipped with a HT sample injector at an acquisition rate of < 500 events/s.
Software	Mass cytometry data were normalized, concatenated, and de-barcoded using CyTOF Software v.7.0 (Standard BioTools, South San Francisco, CA, USA). FCS files were manually processed in FlowJo v10 (TreeStar, Ashland, OR, USA) to exclude Ce140+ beads, Gaussian ion cloud fusion events, debris, Pt198+ dead cells, doublets (Supplementary Figure S6) and exported into new FCS files which was used for downstream analysis.
Cell population abundance	Dead cells were stained using AQUA live/dead dye (Invitrogen) and excluded from the analysis.
Gating strategy	We used a combination of traditional manual gating validation (outlined in Supplementary Figure S7) and unbiased approaches to analyze our datasets

☒ Tick this box to confirm that a figure exemplifying the gating strategy is provided in the Supplementary Information.
